# Astrocyte-mediated switch in spike timing-dependent plasticity during hippocampal development

**DOI:** 10.1038/s41467-020-18024-4

**Published:** 2020-09-01

**Authors:** Rafael Falcón-Moya, Mikel Pérez-Rodríguez, José Prius-Mengual, Yuniesky Andrade-Talavera, Luis E. Arroyo-García, Rocío Pérez-Artés, Pedro Mateos-Aparicio, Sónia Guerra-Gomes, João Filipe Oliveira, Gonzalo Flores, Antonio Rodríguez-Moreno

**Affiliations:** 1 Laboratory of Cellular Neuroscience and Plasticity, Department of Physiology, Anatomy and Cell Biology, Universidad Pablo de Olavide, ES-41013 Seville, Spain; 2 Instituto de Fisiología, Benemérita Universidad Autónoma de Puebla, Puebla, México; 3 Life and Health Sciences Research Institute (ICVS), School of Medicine, University of Minho, 4710-057 Braga, Portugal; 4 ICVS/3Bs - PT Government Associate Laboratory, Braga/Guimarães, Portugal; 5 IPCA-EST-2Ai, Polytechnic Institute of Cávado and Ave, Applied Artificial Intelligence Laboratory, Campus of IPCA, Barcelos, Portugal

**Keywords:** Neuroscience, Synaptic plasticity, Spike-timing-dependent plasticity

## Abstract

Presynaptic spike timing-dependent long-term depression (t-LTD) at hippocampal CA3-CA1 synapses is evident until the 3^rd^ postnatal week in mice, disappearing during the 4^th^ week. At more mature stages, we found that the protocol that induced t-LTD induced t-LTP. We characterized this form of t-LTP and the mechanisms involved in its induction, as well as that driving this switch from t-LTD to t-LTP. We found that this t-LTP is expressed presynaptically at CA3-CA1 synapses, as witnessed by coefficient of variation, number of failures, paired-pulse ratio and miniature responses analysis. Additionally, this form of presynaptic t-LTP does not require NMDARs but the activation of mGluRs and the entry of Ca^2+^ into the postsynaptic neuron through L-type voltage-dependent Ca^2+^ channels and the release of Ca^2+^ from intracellular stores. Nitric oxide is also required as a messenger from the postsynaptic neuron. Crucially, the release of adenosine and glutamate by astrocytes is required for t-LTP induction and for the switch from t-LTD to t-LTP. Thus, we have discovered a developmental switch of synaptic transmission from t-LTD to t-LTP at hippocampal CA3-CA1 synapses in which astrocytes play a central role and revealed a form of presynaptic LTP and the rules for its induction.

## Abstract (web summary)

Presynaptic spike timing-dependent long-term depression at hippocampal CA3-CA1 synapses is evident until the third postnatal week in mice. The authors show that maturation beyond four weeks is associated with a switch to long-term potentiation in which astrocytes play a central role.

## Introduction

The mammalian brain has the ability to change in response to experience, a property termed plasticity^1^. Plasticity involves the re-organization of cortical maps during development, and is fundamental for learning and memory^2,3^. Throughout development, activity sensory-dependent plastic changes occur during permissive and critical periods of plasticity, with environmental influences subsequently shaping brain circuits further, reordering and refining neural connections into the definitive adult circuits^4^. The closing of such permissive windows is associated with the loss of plasticity at particular synapses, producing specific functional effects^4,5^. Long-term potentiation (LTP) and long-term depression (LTD) of synaptic transmission are the two best-known forms of plasticity.

Spike timing-dependent plasticity (STDP) is a Hebbian form of long-term synaptic plasticity detected in all species examined to date, from insects to humans. This process is a strong candidate to underlie circuit remodeling during development, as well as that in subsequent learning and memory^6^. In STDP, the order and relative millisecond timing of pre- and postsynaptic action potentials (APs, spikes) determines the direction and magnitude of synaptic changes. Thus, timing-dependent LTP (t-LTP) occurs when a presynaptic spike is followed by a postsynaptic spike, whereas timing-dependent LTD (t-LTD) is induced when this order is inverted, although exceptions exist^6,7^.

A presynaptic form of t-LTD that requires the activation of presynaptic NMDA receptors (preNMDARs) has been described in the hippocampus and in the visual and somatosensory cortices^7–11^. These forms of t-LTD are expressed presynaptically and they disappear during the 3rd–5th weeks of postnatal development^7–12^. In the mouse hippocampus, t-LTD is lost by the fourth week of postnatal development (P22–30)^13^ and the mechanism underlying this loss was recently defined^12^. However, what happens later in development, after the loss of this t-LTD has not yet been explored.

To better understand the mechanisms that underlie the changes in plasticity during development, here we explore what occurs after P30 at hippocampal CA3–CA1 synapses. Surprisingly, we find that a post-before-pre protocol that induces t-LTD at P8–21 fails to induce plasticity at P22–30, yet it induces t-LTP at P35–42. Characterizing this form of t-LTP, the result of a switch from presynaptic t-LTD, coefficient of variation, number of failures, paired-pulse ratio (PPR) and miniature responses analyses demonstrate its presynaptic nature. Furthermore, this form of presynaptic t-LTP does not require NMDARs but requires mGluR5 activation. We also find that this presynaptic t-LTP requires the flux of calcium through postsynaptic L-type calcium channels, as well as calcium release from intracellular stores. Moreover, this form of t-LTP does not require eCB signaling or CB_1_Rs, yet it does require nitric oxide (NO) as a retrograde messenger. We also find that astrocytes are involved in the induction of this form of t-LTP and that they fulfill a central role in creating the conditions for this form of preLTP to be induced. The switch from presynaptic t-LTD to presynaptic t-LTP occurs owing to the increased inhibition of presynaptic release associated with maturation and it is mediated through the activation of presynaptic type 1 adenosine receptors (A_1_Rs) by adenosine from astrocytes, which considerably dampens the probability of glutamate release. In addition, astrocytes seem to not only release adenosine but also glutamate, presumably to activate mGluRs, to induce t-LTP. Thus, we discover here a developmental switch from presynaptic depression to presynaptic potentiation of synaptic transmission with hippocampal maturation, and uncover a form of presynaptic t-LTP and the mechanism by which it is induced.

## Results

### A switch from t-LTD to t-LTP occurs at P35–42

**Fig. 1:**
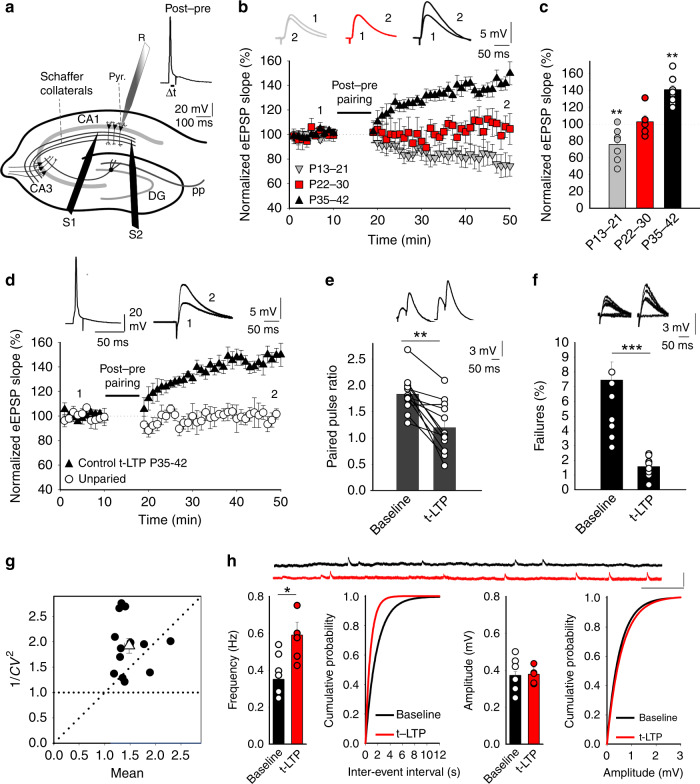
Presynaptic t-LTD at P13–21 in the hippocampus switches to t-LTP at P35–42. **a** Left, scheme showing the general experimental set-up: R, recording electrode; S1 and S2, stimulating electrodes; right, pairing protocol utilized (Δt, time between the peak of the spike and EPSP onset). **b** A post-pre single-spike pairing protocol induces t-LTD at P13–21 (gray triangles, *n* = 7). This t-LTD is evident during the third week of development but it disappears during the fourth week (red squares, *n* = 6) and switches to t-LTP during the fifth week (black triangles, *n* = 13). The EPSP slopes monitored are shown. Traces show the EPSP before (1) and 30 min after (2) pairing. **c** Summary of the results. **p < 0.01, two-sided Student’s *t* test. **d** A post-pre protocol induces t-LTP at P35–42. The EPSP slopes monitored in paired (black triangles, *n* = 13) and unpaired pathways (white circles, *n* = 13) are shown. The traces show the EPSP before (1) and 30 min after (2) applying the induction protocol in the paired pathway, only the paired pathway showed t-LTP. **e**–**h** t-LTP induced by a post-pre protocol at P35–42 is expressed presynaptically. PPR decreases after t-LTP: sample traces at baseline and 30 min after induction of t-LTP, *n* = 13, **p < 0.01, two-sided Student’s *t* test. For detailed PPR values see Supplementary Data 1 **e**. The number of failures decreases after t-LTP induction **f**. Sample traces at baseline and 30 min after induction of t-LTP (*n* = 10, ***p < 0.001, two-sided Student’s *t* test). **g** Normalized plot of CV^−2^ versus mean EPSP slope yields data points mainly above the diagonal after induction of t-LTP (*n* = 13). **h** Miniature EPSP (mEPSPs) monitored during the baseline and after t-LTP induction in the presence of TTX (500 nm). Histograms and cumulative graphs show that after t-LTP induction, the frequency of mEPSPs increases, whereas the amplitude of mEPSP remains constant (*n* = 6). Scale bars: 1 mV, 1 s. *p < 0.05, two-sided Student’s *t* test. The error bars represent the S.E.M. Source data are provided as a Source Data file.

It was recently shown that t-LTD is induced at CA3–CA1 hippocampal synapses at P13–21, whereas no plasticity is induced at P22–30^12^. We confirmed this in slices prepared from the mouse hippocampus at P13–21, monitoring the excitatory postsynaptic potentials (EPSPs) evoked by extracellular stimulation of Schaffer collaterals in the stratum radiatum (StR) by whole-cell recording of CA1 pyramidal cells (PCs) (Fig. 1a, b). When 100 pairings of single EPSPs and single postsynaptic spikes at 0.2 Hz were applied, t-LTD was clearly induced in current–clamp mode. Thus, a post-before-pre pairing protocol (post-pre protocol), with a postsynaptic spike arising ~18 ms before the presynaptic stimulation, inducing robust t-LTD (75 ± 7%, *n* = 7), whereas an unpaired control pathway had no such effect (101 ± 6%, *n* = 7: Fig. 1b, c). No t-LTD was observed at P22–30 (paired pathway 102 ± 6%, *n* = 6, unpaired pathway 103 ± 6%, *n* = 6: Fig. 1b, c), yet when the same protocol was applied at P35–42 a robust t-LTP was surprisingly observed (141 ± 5%, *n* = 13 vs 100 ± 4%, *n* = 13 in the unpaired pathway, Fig. 1b–d). From P31–34, no plasticity or t-LTP was observed in ~50% of the cases (48%: no plastic change, 52%: t-LTP), indicating this is a transition interval from no-LTD (no plasticity) to t-LTP). For this reason, we include here only values from P35–42 when t-LTP is observed practically in 100% of the cases. These results indicate that there is a switch from t-LTD to t-LTP at CA3–CA1 synapses during development.

### Presynaptic expression of t-LTP

To determine the locus of expression of this form of hippocampal t-LTP, we combined several approaches. First, we analyzed the PPRs at baseline and 30 min after the pairing protocol was applied, identifying a significant decrease in the PPR after t-LTP indicative of a presynaptic change (from 1.83 ± 0.10 at baseline to 1.22 ± 0.13; *n* = 13, Fig. 1e). Second, we observed failures in synaptic transmission in several experiments (*n* = 10) and when we analyzed whether the number of failures change after t-LTP, a consistent decrease in the number of failures was observed (from 7.5 ± 1.2% at baseline to 1.6 ± 0.2% after t-LTP, *n* = 10), again suggesting a presynaptic locus for this process (Fig. 1f). Third, we estimated the noise-subtracted CV of the synaptic responses before and after t-LTP induction. A plot of CV^−2^ versus the change in the mean evoked EPSP slope (M) before and after t-LTP yielded points mainly above the diagonal line consistent with a presynaptic modification of release parameters^14–16^, Fig. 1g). Finally, we recorded and analyzed miniature responses (mEPSP) in the presence of 500 nm tetrodotoxin (TTX), before and after the induction of t-LTP by adding TTX during baseline, washing out TTX and performing the t-LTP experiment and finally adding TTX again. In this experimental condition we found t-LTP similar to previous experiments (146 ± 12%, *n* = 6 vs 103 ± 5%, *n* = 6 in the unpaired pathway). In this experiment, we found that after t-LTP induction, the frequency of mEPSP increased (baseline 0.36 ± 0.05 Hz, *n* = 6; after t-LTP induction 0.59 ± 0.05 Hz, *n* = 6, Fig. 1h) with no effect on mEPSP amplitude (baseline 0.370 ± 0.003 mV, *n* = 6; after *t* LTP induction 0.380 ± 0.02 mV, *n* = 6, Fig. 1h. These results again suggesting a presynaptic locus for t-LTP expression with no change in the postsynaptic parameter Q.

Together, these results are consistent with an increase in the probability of neurotransmitter release in the paired pathway and indicative of a presynaptic locus for this form of t-LTP.

### The switch occurs across a range of spike timings

**Fig. 2:**
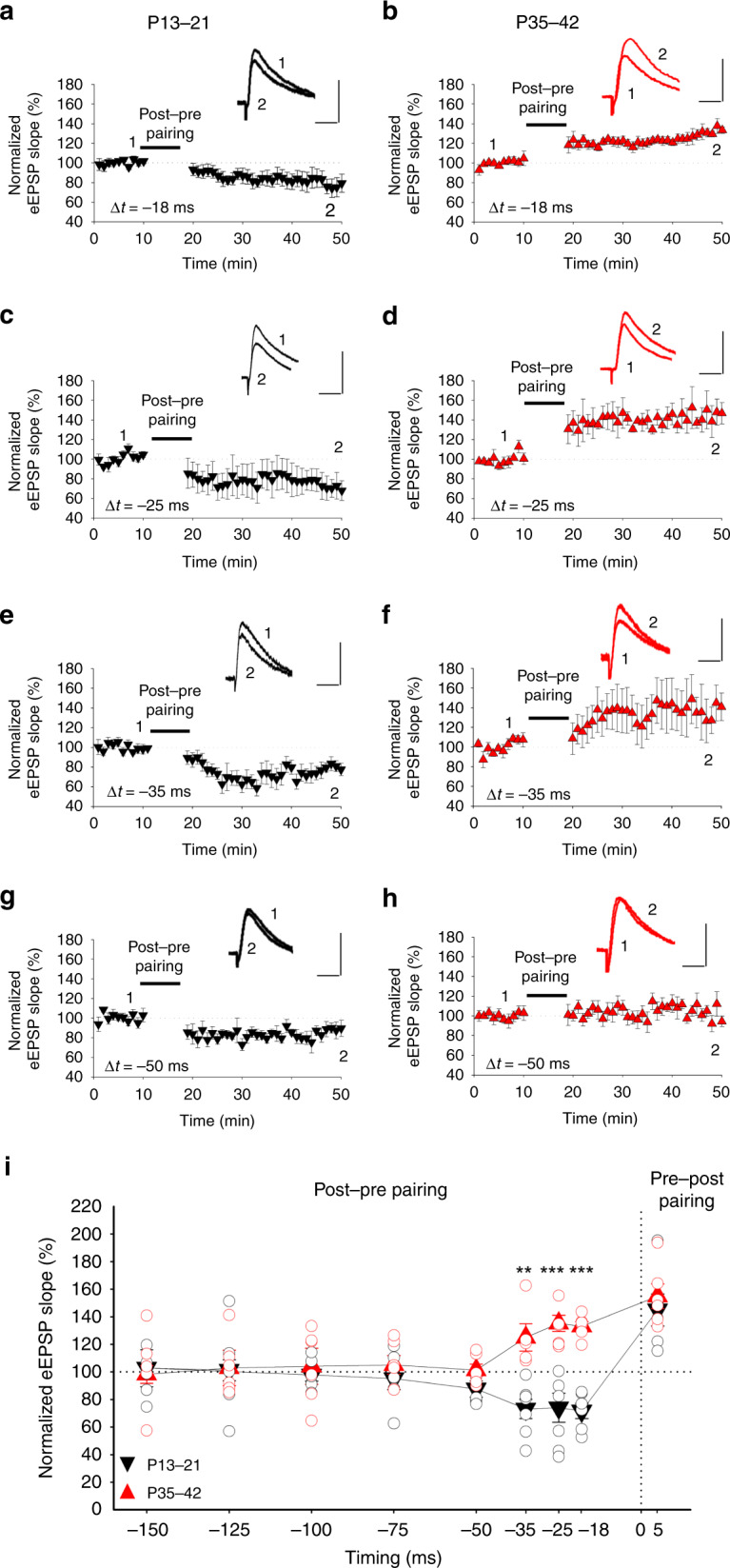
Time window for STDP at P13–21 and P35–42. A post‐before‐pre pairing protocol at P13–21 (black) induces t‐LTD for Δt = −18 ms (*n* = 6) **a**, −25 ms (*n* = 6) **c** and −35 ms (*n* = 7) **e** but not for −50 ms (*n* = 6) or a more negative Δt. **g** At P35–42 (red), the same protocol induces t-LTP for Δt = −18 ms (*n* = 6) **b**, −25 ms (*n* = 6) **d** and −35 ms (*n* = 5) **f** but not for −50 ms (*n* = 6) or a more negative Δt. **h** Δt: time interval between EPSP onset and peak of postsynaptic spike. The EPSP slopes monitored before and after the pairing protocol are shown. Traces show eEPSPs before (1) and 30 min after (2) the pairing protocol. Scale bars: 5 mV, 50 ms. **i** Summary plot showing the time window for STDP at P13–21 (black triangles) and P35–42 (red triangles). **p < 0.01; ***p < 0.001, two-sided Student’s *t* test. The error bars represent the S.E.M. Source data are provided as a Source Data file.

Next, we wanted to determine whether the observed switch occurred just at the time interval between spikes studied (−18 ms) or whether it happens across a range of spike timings. Thus, to better characterize the time windows for this switch, we performed experiments using different timings between presynaptic and postsynaptic activity as a protocol to induce a t-LTD switch to t-LTP, ranging from −150 to +5 ms (Fig. 2a–i). At +5 ms (pre-post protocol), a clear t-LTP was observed at both P13–21 and P35–42 (145 ± 12%, *n* = 6 at P13–21 and 155 ± 9%, *n* = 6 at P35–42, Fig.2i). At −150, −125, −100, or −75 ms, when a post-pre protocol was applied, no t-LTD was observed at P13–21 (103 ± 13%, *n* = 6, 101 ± 8%, *n* = 6, 98 ± 5%, *n* = 6, 95 ± 8%, *n* = 6, respectively, Fig. 2i) or P35–42 (98 ± 6%, *n* = 6, 103 ± 13%, *n* = 6, 104 ± 11%, *n* = 6, 105 ± 7%, *n* = 6, respectively, Fig. 2i). At −50 ms, a small magnitude t-LTD was observed (88 ± 6%, *n* = 6, Fig. 2g) at P13–21, that did not switch to t-LTP when the same experiment was performed at P35–42 (101 ± 4%, *n* = 6, Fig. 2h). At −35 and −25 ms, a strong t-LTD was observed at P13–21 (73 ± 7%, *n* = 6; 72 ± 9%, *n* = 6; respectively, Fig. 2c, e). When the experiments were repeated using these timings but at P35–42, a switch to t-LTP was observed in both cases (125 ± 9%, *n* = 6; 131 ± 6%, *n* = 6, Fig. 2d, f) as occurred at −18 ms (P13–21: 72 ± 6%, *n* = 6; P35–42: 133 ± 3%, *n* = 6, Fig. 2a, b). These results indicate that t-LTD is observed only at P13–21 and that the observed switch from t-LTD to t-LTP is common to different time intervals (−18 to −35 ms), and that the t-LTP is not the result of a timing shift occurring with development (Fig. 2). In Fig. 2i, a STDP plasticity window for this presynaptic form of LTP is shown.

### Presynaptic t-LTP requires mGluRs but not NMDARs

**Fig. 3:**
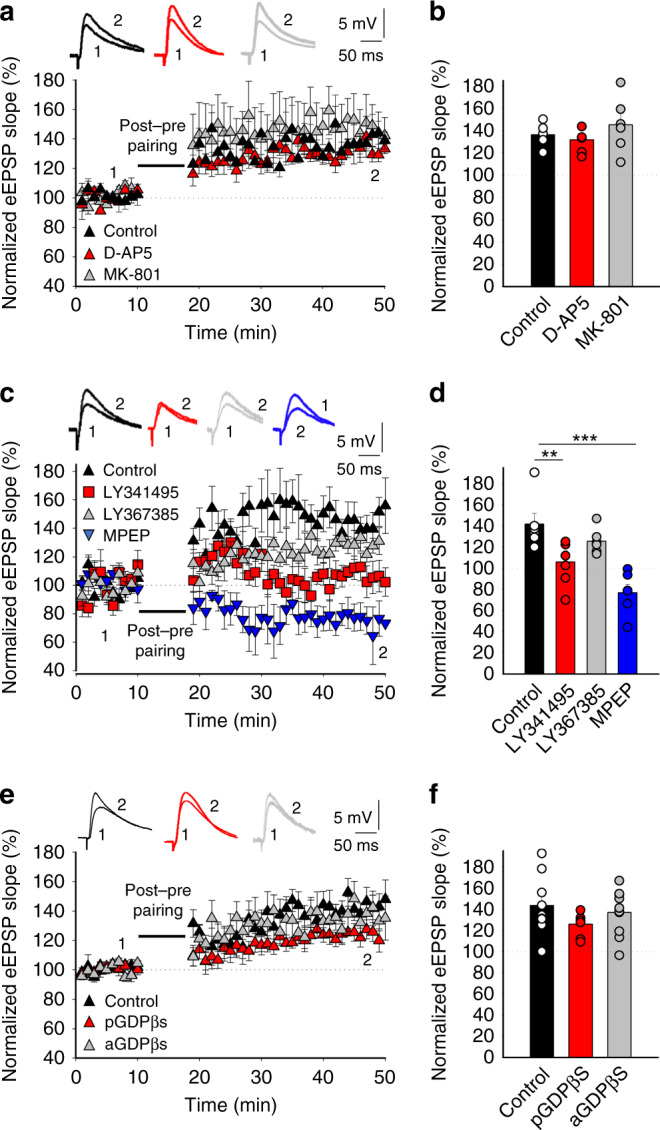
Presynaptic t-LTP requires metabotropic glutamate receptors but not NMDARs. **a** Addition of D-AP5 (50 µm) to the superfusion fluid did not prevent t-LTP induction. The EPSP slope is shown in D-AP5-treated (red triangles, *n* = 6) and untreated cells (black triangles, *n* = 6). Bath-applied MK-801 (500 µm–1 mm) did not block the induction of t-LTP (gray triangles, *n* = 6). The traces show the EPSP before (1) and 30 min after (2) pairing. **b** Summary of the results. **c** The t-LTP requires mGluR5. The EPSP slopes monitored in control slices (black triangles, *n* = 9) and in slices treated with the mGluR antagonist LY341495 (100 µm, red squares, *n* = 6), the group I mGluR antagonist LY367385 (100 µm, gray triangles, *n* = 6) or the mGluR5 antagonist MPEP (500 µm, blue triangles, *n* = 6) are shown following post-before-pre pairing. The traces show the EPSP before (1) and 30 min after (2) pairing. **d** Summary of the results. **e** The t-LTP requires activation of presynaptic mGluR5s. Time course of t-LTP induction in control conditions (black triangles, *n* = 9) and with the postsynaptic neuron (red triangles, *n* = 6) or astrocytes (gray triangles, *n* = 10) loaded with GDPβS (1 mm). Inset: Traces show the EPSP before (1) and 30 min after pairing (2) in control slices or when the postsynaptic neuron or astrocytes are loaded with GDPβS. **f** Summary of the results. **p < 0.01, ***p < 0.001, one-way ANOVA + Holm–Sidak. Error bars represent the S.E.M. Source data are provided as a Source Data file.

The t-LTD detected in juveniles (P13–21) requires non-postsynaptic, probably presynaptic NMDARs^12,13,17^. By contrast, the presynaptic form of t-LTP that appears at P35–42 was not affected when D-AP5 (50 µm) or MK-801 (0.5–1 mm) were present in the bath (132 ± 4%, *n* = 6 and, 145 ± 10%; *n* = 6, respectively, vs interleaved controls, 136 ± 4%; *n* = 6, Fig. 3a, b). Hence, the t-LTP induced by a post-pre protocol at P35–42 does not require pre- or postsynaptic NMDARs. As the t-LTP identified did not require NMDARs, we explored its dependency on other glutamate receptors. As mGluRs have been implicated in plasticity and LTP in different regions and at distinct synapses^18^, we tested whether this form of presynaptic t-LTP at CA3–CA1 synapses also required mGluRs. Significantly, t-LTP was completely blocked in the presence of the broad-spectrum mGluR antagonist LY341495 (100 µm, 104 ± 8%; *n* = 6: Fig. 3c, d). Moreover, t-LTP was not prevented by treating the slices with the mGluR1 antagonist LY367385 (100 µm, 125 ± 5%, *n* = 6), yet it was prevented by the specific mGluR5 antagonist MPEP (20–40 µm, 77 ± 8%, *n* = 6, relative to interleaved slices for the three experimental conditions, pooled together, 142 ± 9%, *n* = 9, Fig. 3c, d), indicating that t-LTP requires mGluR5. To determine whether the mGluRs involved in t-LTP induction are postsynaptic, we repeated the experiments with the postsynaptic neuron loaded with GDPβS to prevent G-protein-mediated signaling. However, t-LTP induction was surprisingly not affected by this treatment (125 ± 5%, *n* = 6, versus interleaved control slices with no GDPβS loaded into the postsynaptic cells, 143 ± 9%, *n* = 9: Fig. 3e, f). When these experiments were repeated with GDPβS loaded into astrocytes, again t-LTP was not affected (137 ± 7%, *n* = 10: Fig. 3e, f), yet the t-LTD at P13–21 was prevented when postsynaptically loaded with GDPβS (73 ± 5%, *n* = 5 in control experiments vs 98 ± 6%, *n* = 6 in postsynaptically GDPβS-treated slices), as reported previously, indicating the compound is working in these experiments^13^. The fact that blocking postsynaptic and astrocytic G-protein-dependent signaling did not affect t-LTP induction, whereas mGluR antagonists in the bath altered this phenomenon, suggests that the mGlu5R necessary for t-LTP induction with a post-pre protocol are most probably located at presynaptic neurons.

It is interesting to note that this form of t-LTP is not the same, nor is it related to the t-LTP described previously and obtained through a pre-post protocol at the same synapses in slices from young animals^13,19^. Indeed, this t-LTP was found to still be present at P35–42 (183 ± 13%, *n* = 12) and to still be dependent on postsynaptic NMDA receptor activation as it was blocked by D-AP5 (100 ± 3%, *n* = 6) and by MK-801 (0.5–1 mm) either in the bath (101 ± 6%, *n* = 6) or loaded into the postsynaptic cell (95 ± 5%, *n* = 6). In addition, by measuring changes in the PPR, we found that this form of t-LTP was expressed postsynaptically (PPR 1.7 ± 0.09 at baseline and 1.4 ± 0.13%, 30 min after t-LTP, *n* = 12, Supplementary Fig. 1). Thus, the presynaptic form of t-LTP we found here coincides temporally with the “more classic” form of postsynaptic t-LTP dependent on NMDAR activation that was described previously at these synapses in slices from young animals. Hence, the two forms of spike t-LTP (pre- and postsynaptic) are both operative at the same time at hippocampal CA3–CA1 synapses.

### t-LTP requires postsynaptic Ca^2+^

**Fig. 4:**
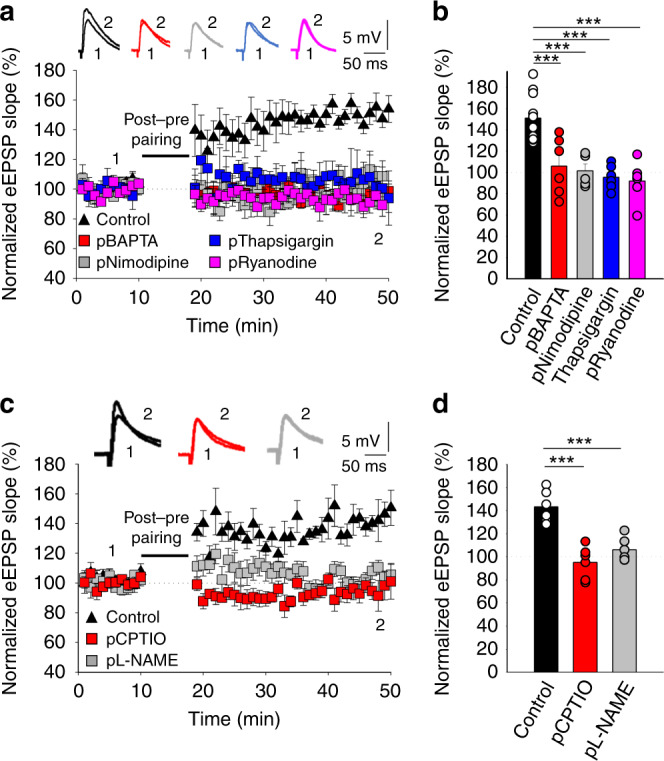
t-LTP requires postsynaptic calcium and NO. **a** t-LTP is prevented by loading BAPTA (20 mm) into the postsynaptic recording pipette. Nimodipine (10 µm), thapsigargin (10 µm) and ryanodine (100 µm) loading into the postsynaptic neuron via the patch pipette prevented t-LTP induction. The EPSP slopes shown were monitored in control slices (black triangles, *n* = 11) and in slices treated with BAPTA (red squares, *n* = 6), nimodipine (gray squares, *n* = 6), thapsigargin (blue squares, *n* = 6) and ryanodine (pink squares, *n* = 6). The traces show the EPSP before (1) and 30 min after (2) pairing. **b** Summary of the results. **c** Time course of the effect of post-before-pre pairing in control conditions (black triangles, *n* = 6) and with cPTIO (100 μm, red squares, *n* = 6) and L-NAME (100 µm, gray squares, *n* = 6) loaded into the postsynaptic neuron. Insets: the traces show the EPSPs before (1) and 30 min after pairing (2). **d** Summary of the results. ***p < 0.001, one-way ANOVA + Holm–Sidak. The error bars represent the S.E.M. Source data are provided as a Source Data file.

Although t-LTP seems not to depend on NMDARs, both t-LTP and t-LTD appear to require postsynaptic Ca^2+^ at neocortical^20,21^ and hippocampal synapses^13^. Therefore, we assessed whether this form of presynaptic t-LTP that emerges at hippocampal CA3–CA1 synapses requires postsynaptic Ca^2+^ by loading the Ca^2+^ chelator BAPTA into the postsynaptic cell via the patch pipette. The inclusion of BAPTA (20 mm) in the recording pipette prevented t-LTP (106 ± 11 %, *n* = 6, versus interleaved controls, 151 ± 6 %, *n* = 11: Fig. 4a, b), indicating that it required postsynaptic Ca^2+^. Since t-LTP requires postsynaptic Ca^2+^ but NMDARs are not the source of this Ca^2+^, we examined how this postsynaptic Ca^2+^ is generated and what is its role in the induction of t-LTP. As L-type Ca^2+^ channels have been implicated previously in plasticity^13^, we evaluated whether they were involved in t-LTP by performing the pairing protocol after bath application of the L-type Ca^2+^ channel blocker, nimodipine (10 µm). The induction of t-LTP was fully blocked in the presence of bath-applied nimodipine (98 ± 11 %, *n* = 6), as it was when nimodipine was loaded into the postsynaptic neuron (101 ± 5 %, *n* = 6, Fig. 4a, b), indicating that like the presynaptic t-LTD, presynaptic t-LTP requires calcium flux through L-type calcium channels into the postsynaptic neuron. In addition, the release of Ca^2+^ from intracellular stores has been described to be required for some forms of t-LTD^13^ and t-LTP at cortical and hippocampal synapses^13,20,21^. Indeed, when we assessed this possibility in the t-LTP induced by a post-pre protocol at P35–42, t-LTP was prevented when the post-pre protocol was applied after loading the postsynaptic neuron with thapsigargin (10 µm) that depletes intracellular Ca^2+^ stores (95 ± 4 %, *n* = 6 versus interleaved controls, 151 ± 6%, *n* = 11; Fig. 4a, b). Furthermore, the inclusion in the pipette of ryanodine (100 µm), a blocker of ryanodine receptors and Ca^2+^-induced Ca^2+^ release from internal stores, prevented the induction of t-LTP (92 ± 8%, *n* = 6, Fig. 4a, b). Hence, Ca^2+^ release from intracellular stores is required for t-LTP.

### t-LTP involves NO from the postsynaptic neuron

Endocannabinoids are synthesized and released by postsynaptic cells in response to depolarization, elevated Ca^2+^ and/or mGluR signaling, and some synapses require eCB signaling and CB_1_R activation for plasticity^22,23^. In fact, it was recently demonstrated that CB_1_R activity is necessary to induce t-LTD at CA3–CA1 synapses at P13–21^13^. To investigate the involvement of cannabinoid signaling in t-LTP, we loaded the postsynaptic neurons with tetrahydrolipstatin (THL, 5 µm), an inhibitor of the eCB synthesizing enzyme diacylglycerol lipase, yet the induction of t-LTP was not affected in these conditions (138 ± 14%, *n* = 6, Supplementary Fig. 2, THL is working in these experiments as in its presence, t-LTD at P13–21 was prevented, control t-LTD: 71 ± 8%, *n* = 5, in THL: 102 ± 8%, *n* = 6, as previously reported in ref. ^13^). When we checked for any direct effect of the eCB 2-AG on t-LTP induction, application of 2-AG did not recover the t-LTP lost after loading the postsynaptic neuron with BAPTA (95 ± 7%, *n* = 7 vs interleaved control slices, 145 ± 8%, *n* = 12, Supplementary Fig. 2). In addition, t-LTP was not affected when induced in the presence of the CB_1_R antagonist AM251 (3 µm), evidence that CB_1_Rs did not participate directly in hippocampal presynaptic t-LTP (154 ± 6%, *n* = 6, versus interleaved slices, 145 ± 8%, *n* = 12, controls pooled together for the THL, p-BAPTA + 2-AG, and AM251 experiments, Supplementary Fig. 2). Thus, unlike presynaptic t-LTD at P13–21, the induction of presynaptic t-LTP at P35–42 does not require CB_1_R activation.

In our experiments, a messenger seemed to be released by the postsynaptic neuron to mediate presynaptic expression of t-LTP and since eCBs appear not to be involved, we set out to identify this substance. One retrograde signal that has been implicated in presynaptic LTP is NO^24–26^ and there is evidence that calcium influx through L-type calcium channels could participate in NO synthesis and/or its release from postsynaptic neurons^24,27^. Significantly, the induction of t-LTP was prevented when the NO scavenger cPTIO (100 µm) had been included in the bath solution or loaded into the postsynaptic neuron via the patch pipette (bath: 102 ± 7%, *n* = 6, postsynaptic neuron: 95 ± 6%, *n* = 6, Fig. 4c, d). Furthermore, presynaptic t-LTP was also prevented when a NO synthase inhibitor, L-NAME (100 µm), was present in the bath 105 ± 9%, *n* = 6) or when was loaded into the postsynaptic neuron, (106 ± 4%, *n* = 6 vs 143 ± 5 %, *n* = 8, in interleaved controls, pooled together for all experimental conditions: Fig. 4c, d), further indicating that t-LTP induction requires NO from the postsynaptic neuron. L-NAME had no effect on t-LTD when it was added to the bath at P13–21 (54 ± 11%, *n* = 6 vs 75 ± 8%, *n* = 6, in interleaved controls, Supplementary Fig. 3). Together, these results indicate that NO produced and released by the postsynaptic neuron is required for t-LTP.

### Presynaptic t-LTP involves astrocyte signalling

**Fig. 5:**
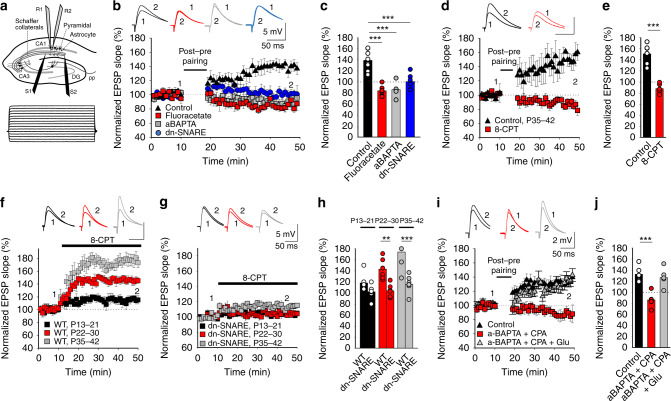
Astrocytes and astroglial adenosine and glutamate involvement in t-LTP. Astrocytes are required for t-LTP. **a** Scheme showing the general experimental set-up: R1 and R2, recording electrodes; S1 and S2, stimulating electrodes; and voltage responses of an astrocyte shown in current clamp. **b** Time course of t-LTP induction in control conditions (black triangles, *n* = 8), and of the loss of t-LTP in the presence of fluoroacetate (10 mm, red squares, *n* = 6), in BAPTA-treated astrocytes (20 mm, gray squares, *n* = 6) and in dnSNARE mutant mice (blue circles, *n* = 6). The traces show the EPSP before (1) and 30 min after pairing (2). **c** Summary of the results. ***p < 0.001, One-way ANOVA + Holm–Sidak. **d** Presynaptic A_1_R-mediated inhibition driven by adenosine released by astrocytes increases with maturation. Time course of t-LTP induction in control conditions (black triangles, *n* = 6), and of the loss of t-LTP in 8-CPT-treated slices (2 µm, red squares, *n* = 6). The traces show the EPSP before (1) and 30 min after pairing (2). Scale bars: 5 mV, 50 ms. **e** Summary of the results. ***p < 0.001, two-sided Student’s *t* test. 8-CPT gradually increases the evoked EPSP slope at P13–21 (black squares, *n* = 7), P22–30 (red squares, *n* = 6) and P35-42 (gray squares, *n* = 6) in WT **f** but not in dn-SNARE mice (P13–21: black squares, *n* = 6; P22–30: red squares, *n* = 6; P35–42: gray squares, *n* = 7) **g**. **h** Summary of the results. **p < 0.01; ***p < 0.001, two-sided Student’s *t* test. **i** The release of glutamate and adenosine by astrocytes is necessary for presynaptic t-LTP induction. Time course of the effect of post-before-pre pairing in control conditions (black triangles, *n* = 6), and in slices with BAPTA loaded into astrocytes and exposed to CPA (50 nm, red squares, *n* = 6), or slices with BAPTA loaded astrocytes exposed to CPA and glutamate puffs (100 µm, gray triangles). Insets: The traces show the EPSPs before (1) and 30 min after pairing (2). **j** Summary of the results. ***p < 0.001. One-way ANOVA + Holm–Sidak. The error bars represent the S.E.M. Source data are provided as a Source Data file.

Astrocytes are implicated in t-LTD at the synapses studied here^13^, participating in the closing of the windows of plasticity with maturation^12^. Thus, we assessed whether astrocyte activation is also necessary to induce the presynaptic form of t-LTP that appears after P35. Three different approaches were used on slices from P35–42 mice (Fig. 5a–c), first preincubating the slices for 1 h with the gliotoxin fluoroactetate (10 mm), which completely abolished t-LTP (84 ± 4%, *n* = 6, Fig. 5b, c). Next, individual astrocytes were loaded with 20 mm of the Ca^2+^ chelator BAPTA through a patch pipette to inhibit vesicle and Ca^2+^-dependent gliotransmitter release from these astrocytes^28^. The recording of CA1 pyramidal neurons demonstrated how BAPTA loading impaired the induction of t-LTP in proximal CA1 PCs at a distance of 50–100 μm (86 ± 5%, *n* = 6, Fig. 5a–c). Finally, we assessed this phenomenon in P35–42 dnSNARE mutant mice in which there is no functional vesicular gliotransmitter release^29–31^. In contrast to the typical t-LTP observed at P35-42 CA3–CA1 synapses in WT mice (139 ± 6%, *n* = 8, pooled), t-LTP could not be induced at this age in these dnSNARE mice (101 ± 6%, *n* = 6, Fig. 5b, c). Together, these results clearly indicate that astrocytes are required for t-LTP induction and the switch from t-LTD to t-LTP.

### t-LTP involves enhanced inhibition of presynaptic release

Inhibition appears to be crucial for the windows of plasticity at different synapses^4,5^. Both GABAergic and adenosine receptor-mediated inhibition have  been linked to the closure of plasticity windows^5,12,19,32–35^, and both GABAergic and adenosine receptor-mediated inhibition seem to become enhanced with maturation^12,34,36,37^. Here, we studied how GABAergic-dependent inhibition affected the switch from t-LTD to t-LTP by evaluating the effect of the GABA_A_ receptor antagonist bicuculline (20 µm) and the GABA_B_ receptor antagonist SHC-50911 (50 µm) on t-LTP induction at P35-42. In the presence of these antagonists t-LTP was still evident (bicuculline 185 ± 12%, *n* = 7; SHC-50911 150 ± 12%, *n* = 7; interleaved slices 173 ± 18%; *n* = 6, Supplementary Fig. 4), indicating that enhanced GABAergic inhibition was not responsible for the switch from t-LTD to t-LTP at CA3–CA1 synapses during the fifth week of development.

As we recently demonstrated that the activation of presynaptic A_1_Rs is responsible for the loss of t-LTD at P22–30^12^, we assessed whether 8-CPT, an antagonist of A_1_R, affected t-LTP. This t-LTP was fully impaired in the presence of this compound (88 ± 4%, *n* = 6, vs 151 ± 7%, *n* = 6 in interleaved control slices, Fig. 5d, e). At P13–21, 8-CPT did not affect t-LTD (77 ± 3%, *n* = 6 vs  73 ± 2%, *n* = 6 in interleaved control slices), and still recovered lost t-LTD at P22–30 (76 ± 5%, *n* = 7 vs 112 ± 4%, *n* = 6 in interleaved control slices, Supplementary Fig. 5). When the EPSP slope was measured in the presence of this A_1_R antagonist, the effect of 8-CPT was stronger at P22–30 than at P13–21. If extracellular adenosine levels continue to increase as development proceeds, a stronger effect on presynaptic A_1_Rs would be expected at P35-42. Indeed, the EPSP slope increased more in the presence of 8-CPT at this later age than at P13–21 or P22–30 (P13–21: 118 ± 6%, *n* = 7; P22–30: 143 ± 5%, *n* = 6); and P35-42 (174 ± 11%, *n* = 6, Fig. 5f–h). These results indicate that there is an increase in presynaptic A_1_R-mediated inhibition with maturation. In the presence of 8-CPT, a decrease in the number of failures in transmission was observed at P13–21 (15 ± 5% baseline, 10 ± 3%, in the presence of 8-CPT, *n* = 6, *p* < 0.05) and P22–30 (18 ± 4% baseline, 5 ± 3%, in the presence of 8-CPT, *n* = 6, *p* < 0.01) and at P35–42 (20 ± 4% baseline, 1 ± 1% in the presence of 8-CPT, *n* = 6, *p* < 0.001), suggesting a presynaptic action of adenosine on A_1_Rs. We also studied the effect of 8-CPT in dnSNARE mice to determine whether the ATP/adenosine that activates A_1_Rs originates from astrocytes and we found that 8-CPT had practically no effect on the EPSP slope in dnSNARE mice at any of the ages studied (102 ± 5%, *n* = 6; 104 ± 5%, *n* = 6; 116 ± 6%, *n* = 7 at P13–21; P22–30 and P35–42, respectively, Fig. 5f–h). Indeed, 8-CPT did not affect eEPSP amplitude at P35–42 when astrocytes were loaded with BAPTA (103 ± 8%, *n* = 6).

### A_1_R activation at P13–21 converts t-LTD into t-LTP

If higher extracellular adenosine concentration during development more strongly activates presynaptic A_1_R at CA3–CA1 hippocampal synapses dampening glutamate release and mediating a switch from t-LTD to t-LTP at P35-42, it could be possible to convert t-LTD in t-LTP earlier in the development by enhancing A_1_R activation (e.g., at P13–21 when t-LTD is robust or at P22–30 when t-LTD is lost). We have previously demonstrated that CPA 20–30 nm is not able to convert t-LTD into t-LTP but that it is able to prevent t-LTD induction at P13–21^12^. To determine whether increasing the concentration of CPA converts t-LTD into t-LTP, we increased CPA concentration to 50 nm; in this experimental condition, CPA is able to produce a switch from t-LTD (at P13–21) or from no-LTD (at P22–30) to t-LTP (from 76 ± 3%, *n* = 7, to 151 ± 12%, *n* = 7 at P13–21, and from 102 ± 2%, *n* = 6, to 131 ± 7%, *n* = 6, at P22–30, Supplementary Fig. 6). To further confirm a presynaptic locus for adenosine on A_1_R we performed experiments studying the effect of CPA 50 nm on mEPSP frequency. In this experimental condition, a clear decrease in the frequency of mEPSP (0.55 ± 0.03 Hz during baseline; 0.22 ± 0.02 Hz in the presence of CPA, *n* = 6) was observed with not effect of mEPSP amplitude (0.33 ± 0.03 mV during baseline; 0.38 ± 0.03 mV in the presence of CPA, Supplementary Fig. 6), indicating that the effects of CPA are indeed presynaptic and thus the activation of presynaptic A_1_R dampens glutamate release that increases with development. These results are in agreement with a primary involvement of A_1_R in the observed switch from t-LTD to t-LTP with maturation.

These results indicate that the adenosine-activating A_1_R is released by astrocytes. As such, adenosine originating from astrocytes activates presynaptic A_1_Rs and depresses glutamate release, an effect that increases with maturation.

### t-LTP requires adenosine and glutamate from astrocytes

It might be expected that if ATP/adenosine was the only gliotransmitter astrocytes release to mediate the switch from t-LTD to t-LTP, when astrocytes were loaded with BAPTA, the A_1_R agonist CPA should be able to recover t-LTP at P35-42 (when no t-LTP would otherwise be observed). However, t-LTP was not recovered by CPA in these conditions (86 ± 5%, *n* = 6 vs 132 ± 5%, *n* = 6 in control slices, Fig. 5i, J), suggesting that another gliotransmitter might also be involved. As presynaptic mGluRs might also participate in the induction of this form of LTP, we tested whether glutamate from astrocytes may be also required to induce presynaptic t-LTP at P35-42 by applying glutamate puffs. When we tested this in slices with BAPTA loaded astrocytes maintained in the presence of CPA, the puffs of glutamate applied recovered t-LTP (127 ± 6%, *n* = 6, Fig. 5i, j). Hence, the gliotransmitters ATP/adenosine and glutamate are necessary for the induction of presynaptic t-LTP.

This glutamate presumably activates presynaptic mGluRs and our data suggest that as development proceeds, the probability of glutamate release decreases to the extent that glutamate from astrocytes is needed to activate presynaptic mGluRs so that they may participate in presynaptic t-LTP. We tested whether the probability of glutamate release decreases at CA3–CA1 synapses with maturation by measuring mEPSP frequencies at P13–21 and P35–42 (in the presence of TTX, 500 nm). A clear decrease in the frequency of mEPSPs was evident at P35–42 (frequency: 0.36 ± 0.04 Hz, amplitude: 0.40 ± 0.05 mV, *n* = 6) compared with P13–21 (frequency: 0.55 ± 0.03 Hz, amplitude: 0.33 ± 0.02 mV, *n* = 6, Supplementary Fig. 7), yet with no effect on the amplitude. This phenomenon was prevented by treating the P35–42 slices with 8-CPT (frequency 0.50 ± 0.03 Hz, amplitude 0.34 ± 0.02 mV, *n* = 6: Supplementary Fig. 7).

A similar result was found when evoked responses were studied during development. We studied PPR of evoked responses from P13–21 to P35–42 mice and observed an increase in PPR at P35–42 when compared with P13–21 (PPR: 1.8 ± 0.07 at P35-42, *n* = 19 and 1.4 ± 0.06, *n* = 11 at P13–21), an increase that was prevented in the presence of 8-CPT (1.27 ± 0.2, *n* = 5, Supplementary Fig. 7). Hence, with maturation there appears to be a decrease in glutamate release probability at CA3–CA1 synapses owing to the adenosine (released by astrocytes)-mediated activation of presynaptic A_1_Rs.

**Fig. 6:**
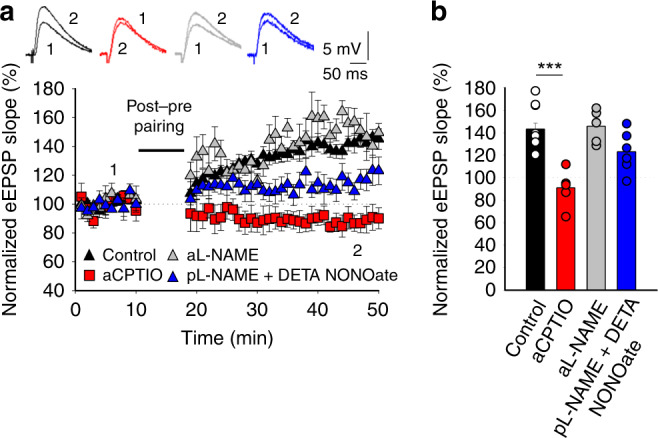
NO is necessary for astrocytic release of gliotransmitter(s) and t-LTP induction. **a** t-LTP is prevented by loading cPTIO (100 µm) into the astrocyte via the recording pipette but not by loading L-NAME (100 µm). When L-NAME is loaded into the postsynaptic neuron and a NO donor (DETA NONOate, 5 mm) added in forms of puffs over astrocytes, t-LTP is recovered. The EPSP slopes monitored in paired control slices (black triangles, *n* = 11) are shown and in slices treated with acPTIO (red squares, *n* = 6), aL-NAME (gray triangles, *n* = 6) and pL-NAME + DETA NONOate (blue triangles, *n* = 6) are shown. The traces show the EPSP before (1) and 30 min after (2) pairing. **b** Summary of the results. ***p < 0.001, one-way ANOVA + Holm–Sidak. The error bars represent the S.E.M. Source data are provided as a Source Data file.

**Fig. 7:**
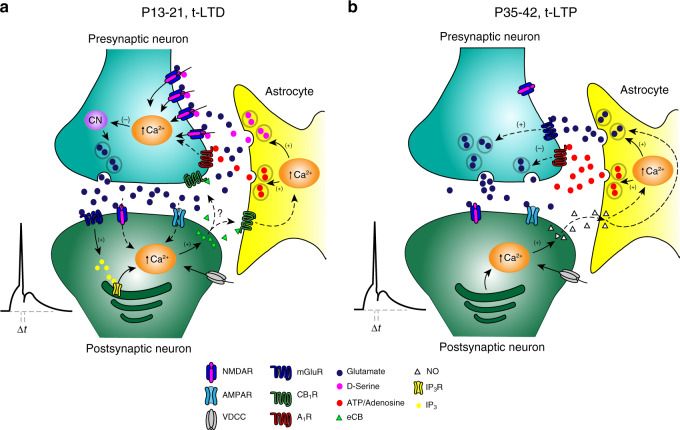
Model showing the switch from t-LTD to t-LTP occurring with maturation at CA3–CA1 synapses of the hippocampus. **a** At P13–21, a known presynaptic form of t-LTD is induced by a post-pre single-spike pairing protocol. In this presynaptically expressed form of t-LTD, postsynaptic action potentials activate voltage-dependent Ca^2+^ channels (VDCCs) and the presynaptically released glutamate activates postsynaptic mGluR, activating PLC and provoking Ca^2+^ release from internal stores and DAG production, which serves a precursor for endocannabinoids (eCBs) synthesis. For t-LTD, eCB signal is necessary to activate CB_1_ receptors to facilitate d-serine release from astrocytes. Together with the glutamate released from presynaptic neurons, this d-serine is known to activate preNMDAR on Schaffer collateral boutons, leading to an increase in presynaptic Ca^2+^, calcineurin activation and synaptic depression. Modified from ref. ^12,13^. **b** In the present study, it has been found that at P35-32, t-LTD is not observed and instead the same protocol (a post-pre protocol) induces presynaptic t-LTP. At this developmental stage, the probability of release has considerably decreased owing to an increase in adenosine release from astrocytes compared with P13–21. Also different to t-LTD induced by the same protocol at P13–21, eCB signaling and NMDAR are not required for presynaptic t-LTP. For the induction of t-LTP, postsynaptic action potentials activates voltage-dependent Ca^2+^ channels (VDCCs) causing calcium release from internal stores, inducing NO synthesis. The NO signal leads to the activation of astrocytes to release glutamate and/or adenosine to activate presynaptic mGluR5 and A_1_R respectively on Schaffer collateral boutons. A_1_R activation considerably reduces neurotransmitter probability release whereas mGluR5 activation leads to a long-lasting increase in glutamate release and synaptic potentiation.

Finally, to define the signal that might stimulate astrocytes to release gliotransmitters and mediate this form of LTP, we evaluated the role of NO, the release of which from the postsynaptic neuron is necessary for t-LTP. NO has previously been shown to increase the calcium that enters and stimulates astrocytes^38^, raising the possibility that NO could activate or interact with astrocytes to release ATP/adenosine and/or glutamate in our conditions. Interestingly, t-LTP induction was prevented by loading the NO scavenger c-PTIO into astrocytes (a-cPTIO 91 ± 6%, *n* = 6) but not when they were loaded with L-NAME (a-L-NAME 146 ± 5%, *n* = 6, vs non-treated control slices 143 ± 5%, *n* = 11, Fig. 6a, b). In addition, when t-LTP induction was prevented by loading the postsynaptic neuron with L-NAME, puffs of the NO donor DETA NONOate (5 mm) on astrocytes recovered t-LTP (123 ± 7%, *n* = 6, Fig. 6a, b). Hence, NO from the postsynaptic neuron might enter the astrocyte to stimulate or modulate the release of gliotransmitters (Fig. 7).

## Discussion

In this study, we show that presynaptic t-LTD switches to presynaptic t-LTP at hippocampal CA3–CA1 synapses across a wide range of spike timings as young mice mature and demonstrate this form of t-LTP is expressed presynaptically and requires the activation of presynaptic mGluR5 but not NMDARs. In addition, this presynaptic t-LTP requires the flux of calcium through postsynaptic L-type calcium channels, as well as calcium release from postsynaptic intracellular stores. Furthermore, this form of presynaptic t-LTP requires postsynaptic NO release as a retrograde signal to astrocytes and astroglial signaling to release ATP/adenosine to activate presynaptic A_1_R and glutamate to activate mGluR (Fig. 7). The number and frequency of spikes necessary to induce STDP in the hippocampus has been controversial and the differences found have been explained by the size of the initial EPSP, the age of animals, the level of inhibition, etc^39,40^. In our results, it is clear that pairing EPSP of 3–5 mV size 100 times with single postsynaptic spikes at 0.2 Hz induces robust plasticity. Although there are number of uncertainties associated with quantal analysis in the CNS^14–16^, in the present work, however, we presented evidence from four different approaches to determine the locus of this form of t-LTP, all consistent with presynaptic changes: failure rate, PPRs, CV and mEPSP frequency, and amplitude analysis. Hence, this form of presynaptically expressed LTP appears during development through a switch from t-LTD, unlike any other form of presynaptic t-LTP (preLTP) described to date, such that our findings expand the repertoire of presynaptic LTPs^24,41,42^. Studying the timing-dependency of this form of t-LTP across a range of spike timings we observed that the switch from t-LTD to t-LTP occurred at different time intervals, from −18 to −35 ms, where there was robust t-LTD at P13–21, indicating this switch happens with maturation and is not owing to a timing interval broadening or shifting along the time axis. Interestingly, in adult human brain slices the STDP curve shows t-LTP at positive and wide negative timing intervals (0, −80 ms), what is in agreement with our results, and suggest that the synaptic learning rules may be conserved (at least in part) across species^43^. Interestingly, we found that the switch from t-LTD to t-LTP is present by using different STDP protocols as a short burst of two spikes at 100 Hz anti-causally paired with a single postsynaptic action potential or when single presynaptic stimulation was anti-causally paired with two postsynaptic actions potentials at 100 Hz (Supplementary Fig. 8). An important question that remains to be approached is whether this switch to t-LTP is related only to STDP or is observed using other protocols that induce LTD at young stages.

Unlike t-LTD, t-LTP appears not to require NMDARs. Rather, it requires the activation of mGluR5 that appear to reside presynaptically. At these synapses, presynaptic mGluRs have been described to bi-directionally modulate glutamate release^44^ and to participate in plasticity^45^. Moreover, glial cells are also thought to have mGluRs that possibly influence plasticity^46,47^, although the data obtained here indicate that astrocyte mGluRs are not involved in this particular form of t-LTP. Nevertheless, our findings make it clear that there is a maturation-associated shift from the involvement of NMDARs to mGluRs in hippocampal plasticity. Forms of preLTP that are dependent on preNMDARs have been described in the hippocampus^41^ and at entorhinal cortex to DG synapses^48–51^ and preLTP forms independent of NMDARs have also been described at hippocampal MF-CA3 synapses^52^, in the cerebellum^53^, thalamus^54^, subiculum^55^, amygdala^56^, and neocortex^57^. The requirement of mGluRs for some forms of preLTP has previously been defined in the hippocampus^45,47^, although these preLTPs were not induced using STDP protocols. At hippocampal CA3–CA1 synapses, a preLTP that is independent of NMDAR has been described^24^, this form of LTP share features with the presynaptic t-LTP we describe here, yet it does not arise through a switch from LTD and it is observed in younger animals when the preLTP we describe is not observed. Importantly, the previously described preLTP does not apparently require glutamate^24^, whereas the pre t-LTP described here clearly depends on glutamate-activating mGluRs. Interestingly, we found that the postsynaptic t-LTP identified previously at the same synapses in young animals using a standard protocol for postsynaptic t-LTP, i.e. a pre-post protocol with 5–10 ms timing^13,19^; is still present at P35–42 and it continues to be dependent on postsynaptic ionotropic NMDAR activation, as well as retaining postsynaptic expression. Importantly, these results indicate that two different forms of t-LTP (one presynaptic and another one postsynaptic) coincide temporally. Similar to other forms of LTP, t-LTP requires a rise in Ca^2+^ in the postsynaptic cell^25^ and resembling the postsynaptic NMDAR-independent forms of LTP described previously^24–26^, t-LTP requires the release of Ca^2+^ from intracellular stores. These features of hippocampal t-LTP are also common to other forms of preLTP^23,38^. Although in our results postsynaptic internal stores are not recruited by mGluRs, mGluR independent forms to recruit calcium from intracellular stores have been demonstrated, as by the activation of voltage-gated Ca^2+^ channels that couple to intracellular Ca^2+^ release by Ca^2+^-induced Ca^2+^ release^58,59^. Despite using three different approaches, we failed to obtain evidence for the participation of the cannabinoid signaling system in this form of LTP and found NO as the retrograde signal produced by the postsynaptic neuron via Ca^2+^ increase, as in other types of preLTP^24–26^.

Through three different approaches, we demonstrated the involvement of astrocytes in presynaptic t-LTP and while astrocytes have been shown to participate in synaptic potentiation^47,49,50^, here they have been proposed to participate in a switch from preLTD to preLTP. Here, we found that enhanced inhibition of presynaptic release mediated by adenosine-activating presynaptic A_1_R and not by GABA receptors is responsible for the switch from t-LTD to t-LTP during the fifth week of development. Interestingly, we found that A_1_R activation at P13–21 converts t-LTD into t-LTP, confirming the important role of adenosine and presynaptic A_1_R in the switch of plasticity observed. As in our results, an increase in the concentration of extracellular adenosine and/or in the activation of A_1_Rs with maturation has been described previously^34,36,37^. While identifying the source of adenosine is complex as it may be released directly from neurons^60–62^ or glial cells^29^ or through glial gap junction hemi-channels^63^ or other mechanisms, our data suggest that adenosine is of astrocytic origin and hence, we propose that presynaptic A_1_R activation augments during development, at least in part owing to the increase in the amount of extracellular adenosine released by astrocytes. This A_1_R activation in turn inhibits glutamate release, reducing the ambient glutamate in association, producing the switch to t-LTP in association with a rise in mGluR activity. Thus, it seems that at P13–21, when neurotransmitter release probability is high, NMDARs are present and activated to mediate a depression of glutamate release, whereas when the probability of release is low and the number of NMDARs has decreased (at P35–42), mGluR receptors are better positioned to respond to the low available extracellular glutamate concentration. This may be possible as metabotropic receptors are well known to amplify weak signals, in this case low extracellular levels of glutamate to mediate a potentiation of glutamate release. In fact, mGluR5 are well coupled to PLC and may affect the exocytotic machinery via PKC^64^ or Munc13 activation, which has been shown to potentiate glutamate release and is directly involved in docking and priming of neurotransmitter vesicles as has been shown for mGluR7^65^. Whether NMDARs are better coupled to mechanisms that decrease than those that increase glutamate release needs to be determined. For the moment the exact molecular mechanisms underlying t-LTP remain unknown. Thus, the increase in adenosine from astrocytes appears to alter glutamate release, synaptic efficacy, and t-LTP. However, although there is a clear requirement for astrocytes to provide adenosine, other sources cannot be ruled out^66^. Whether the increase in extracellular adenosine as the hippocampus matures is due to an increase in the number of astrocytes or to enhanced release, or whether other components that participate in the induction of this form of t-LTP change with maturation merits further study. Hence, it may be possible to control plasticity by manipulating the availability of adenosine^67^, which would make this an interesting target to improve health and learning and memory.

The specific release probability seems to influence the manifestation of LTP, with a higher probability of glutamate release favoring LTD and a lower probability favoring LTP^24^. Thus, a change in the release probability may be the direct presynaptic mechanism responsible for the effects observed. Indeed, we observe a decrease in the frequency of mEPSP and in evoked responses slopes with maturation that depends on A_1_R activation. Accordingly, our data are consistent with reports that when there is a high glutamate release probability, synapses are likely to show preLTD, whereas synapses with a low probability of release are more prone to show preLTP^24,68–72^. The decrease in release probability may contribute to the stabilization of hippocampal circuits. Indeed, a decrease in the probability of release at glutamatergic synapses with development has been demonstrated in the developing neocortex^73,74^, calyx of Held^75^, striatum^76^, and at MF-CA3^77^ and CA3–CA1^78^ synapses in the hippocampus. As such, this phenomenon would appear to represent an essential step in the maturation of glutamatergic synapses. The decrease in the probability of glutamate release found may not occur at all synapses and it may occur in a heterogeneous manner, possibly only at synapses with a high probability of release as indicated previously^78^. Although more work is necessary to understand how this developmental change in release probability occurs, it could be explained by changes in calcium influx and in the expression of presynaptic calcium channels reducing the vesicle release probability^78^.

Adenosine appears not to be the only gliotransmitter necessary for the induction of t-LTP, but rather, ATP/adenosine and glutamate appear to be released together to mediate t-LTP induction. Indeed, individual astrocytes may release both adenosine and glutamate^79^ and by releasing these two gliotransmitters, astrocytes may control hippocampal basal synaptic activity^80^ and tonically depress neurotransmission^29^, probably depressing some synapses and potentiating others^79^. In addition, the possibility exist that glutamate from the presynaptic neuron activates presynaptic mGluR5 too but that this amount of glutamate or mGluR5 activation is not enough to mediate t-LTP and, together with glutamate released from astrocytes are sufficient for t-LTP. Thus, we believe our data have revealed important components of the mechanism underlying the switch to t-LTP in the window of plasticity, opening the way to the pharmacological manipulation of plasticity and of t-LTP, which is likely to be relevant to understand brain function during development. An interesting issue that remains to be properly explained is whether astrocytes release ATP/adenosine and glutamate tonically as maturation proceeds or whether the release of these factors is controlled by direct stimulation, and what is the true role of the postsynaptic neuron in the induction of t-LTP. Although more research will be needed to properly address this question, surprisingly, NO synthesized by the postsynaptic neuron seems to be released and enter the astrocyte, potentially stimulating gliotransmitter release via an increase in calcium flux into the astrocyte^38^. Whether NO potentiates the release of one or both gliotransmitters remains to be determined.

What might be the physiological relevance of this switch in plasticity from presynaptic depression to presynaptic potentiation? The true influence of STDP in the hippocampus remains unclear and further studies will be necessary to determine the specific developmental role of t-LTD and t-LTP in these circuits. t-LTD may be involved in refining synapses and indeed, is thought to play an important role in developmental plasticity, potentially weakening excitatory synapses that are underused or behaviorally irrelevant^81,82^. The form of t-LTP described here is only evident from the fifth week of development, indicating its relevance from early adulthood onwards when it probably influences learning and memory. Presynaptic plasticity may also involve structural changes and may alter the short-term dynamics of neurotransmitter release, contributing to circuit computations, the modification of the excitatory/inhibitory balance and sensory adaptation^42^. Why some synapses, as we observe at CA3–CA1 synapses, show pre- or postsynaptic plasticity has yet to be determined, although this may reflect different computational requirements. As indicated above, presynaptic t-LTP may contribute to circuit computation by changing short-term dynamics and it may shift synapses between low-pass and high-pass filtering modes, thereby changing the computational properties of the synapse^48,83,84^. At somatosensory cortex, L4-L2/3 and L2/3-L2/3 synapses, STDP shows different requirements, indicating that the pre- or post-synaptic expression of plasticity is fundamental for the proper brain circuits functioning and that it is possible they are differently regulated^85,86^. In addition, as predicted by some models^83^, presynaptically expressed t-LTP may increase the trial to trial reliability, and with the t-LTP postsynaptically expressed may induce a larger change in signal-to-noise ratio than postsynaptic changes alone as described in auditory cortex^87^. Furthermore, multiple expression sites may be favorable to the system as it may have more possibilities for plasticity when one is disrupted. Finally, modulators may affect differently the two different forms of LTP, making possible to associate particular behaviors, with a particular locus of expression. Thus, both pre- and postsynaptic mechanisms might contribute to the weight dependence of plasticity^83^. The co-existence of multiple forms of plasticity (pre- and postsynaptic) may also reflect the hierarchical processing of information, potentially allowing memory to be ordered according to its salience, as suggested in the amygdala^88^.

There is almost no information regarding the possible behavioral influence of presynaptic LTP, an emerging field in which data have only been provided for MF-CA3 synapses (where preLTP is implicated in learning and memory^89^) and amygdala synapses (where preLTP is implicated in fear memory formation^90^). At CA3–CA1 synapses, the behavioral role of presynaptic t-LTP remains basically to be determined, however, a recent report has suggested that at CA3–CA1 synapses presynaptic changes within the synaptic engram may be associated with context-dependent fear conditioning, suggesting that preLTP might be associated with learning and memory in vivo^91^. Morphological changes are known to occur during critical periods of plasticity and in adulthood^92,93^, yet whether this form of presynaptic plasticity induces structural plasticity is not clear at present and will require further study.

## Methods

### Mice

All animal procedures were carried out in accordance with the European Union Directive 2010/63/EU regarding the protection of animals used for scientific purposes and were approved by the Ethics Committee of the Universidad Pablo de Olavide and the Ethics Committee of the Andalusian Government. C57BL/6 mice were obtained from Harlan Laboratories (Spain) and postnatal day (P) 13–42 mice of either sex were used. Animals were kept on a continuous 12 h light/dark cycle, at temperatures between 18 and 24 °C at 40–60% humidity, and with full availability of food and water. In some experiments, dominant-negative (dn) SNARE mice^29,30^ of same age intervals were used. These mice were not fed with doxycycline to allow for transgene expression. In these mice, human glial fibrillary acidic protein (hGFAP) promoter mediates the expression of tetracycline trans-activator (tTA) specifically in astrocytes, which will in turn activate the tetO operator driving the expression of the cytosolic fraction of VAMP2/synaptobrevin II and the enhanced green fluorescence protein. Expression of dnSNARE transgenes interferes with the formation of the SNARE complex, resulting on the blockade of exocytosis and impairment of vesicular release in astrocytes^31^.

### Slice preparation

Hippocampal slices were prepared as described previously^12,13^. In brief, mice were anesthetized with isoflurane (2%) and decapitated, and the whole brain containing the two hippocampi was removed and placed in an ice-cold solution containing (in mm): NaCl, 126; KCl, 3; NaH_2_PO_4_, 1.25; MgSO_4_, 2; CaCl_2_, 2; NaHCO_3_, 26; and glucose, 10 (pH 7.2, 300 mOsm L^−1^). Transverse hippocampal microtome slices were obtained (350-μm thick, Leica VT1000S) and maintained oxygenated (95% O_2_/5% CO_2_) in the same solution for at least 1 h before use. Experiments were carried out at 30–34 °C. During the experiments; the slices were superfused continuously with the solution indicated above.

### Electrophysiological recordings

Whole-cell patch clamp recordings of pyramidal cells located in the CA1 field of the hippocampus were obtained under visual guidance by infrared differential interference contrast microscopy. The neurons were verified as PCs through their characteristic voltage response to a current step protocol in current–clamp configuration with a patch clamp amplifier (Multiclamp 700B), acquiring the data with pCLAMP 10.2 software (Molecular Devices). Patch electrodes were pulled from borosilicate glass tubes and had a resistance of 4–7 MΩ when filled with (in mm): potassium gluconate, 110; HEPES, 40; NaCl, 4; ATP-Mg, 4; and GTP, 0.3 (pH 7.2–7.3, 290 mOsm L^−1^). Only cells with a stable resting membrane potential below −55 mV were assessed and the cell recordings were excluded from the analysis if the series resistance changed by >15%. During the experiments, the changes in Vm (1–3 mV) were corrected by imposing continuous current (10–30 pA) to maintain the membrane potential constant. All recordings were low-pass filtered at 3 kHz and acquired at 10 kHz. For plasticity experiments, EPSPs were evoked alternately in two input pathways, test and control, each at 0.2 Hz. Stimulating electrodes were situated at 200–400 µm from cell soma. The EPSPs were induced by two monopolar stimulation electrodes placed in the StR using brief current pulses (200 μs, 0.1–0.2 mA). Stimulation was adjusted to obtain an EPSP peak amplitude of ~3–5 mV in control conditions and pathway independence was assured by the lack of cross-facilitation when the pathways were stimulated alternately at 50 ms intervals. Plasticity was assessed through the changes in the EPSP slope, measured in its rising phase as a linear fit between time points corresponding to 25–30% and 70–75% of the peak amplitude under control conditions. Miniature responses were recorded in the presence of 500 nm TTX.

### Plasticity protocols

After establishing a stable basal EPSP over 10 min, the test input was paired 100 times with a single postsynaptic spike. The single postsynaptic spike was evoked by a brief somatic current pulse (5 ms, 0.1–0.5 pA) and the control pathway was unstimulated during the pairing period. To induce t-LTP, the postsynaptic AP was evoked 18 ms before the onset of the EPSP. EPSP slopes were monitored for at least 30 min after the pairing protocol and the presynaptic stimulation frequency remained constant throughout the experiment. In some experiments we used a pre-post protocol (5–10 ms timing) to induce a postsynaptic form of t-LTP. Where appropriate, “Glutamate Puffs” were applied using a Picospritzer (Parker Hannifin). Glutamate was dissolved in the external solution and puffed through a micropipette over a BAPTA loaded astrocyte at a pressure of 10 psi and for 50–200 ms, which did not affect patch-clamping. For each experiment, 50–100 glutamate puffs were applied at 0.2 Hz after the recording neuron at baseline, 18 ms before the onset of the EPSP. EPSP slopes were monitored for 30 min after the protocol. In some experiments, different timings were used to construct a STDP window. Experiments were repeated a minimum of six times.

### Pharmacology

Pharmacological agents were purchased from: Sigma Aldrich - BAPTA, d-serine, TTX, sodium fluoracetate, CPA, and all the salts used to prepare the internal and external solutions; Tocris Bioscience - (+)-MK-801 maleate, d-AP5, 8-CPT, cPTIO, l-glutamic acid, LY367385, LY341495, MPEP, 2-AG, AM251, L-NAME, DETA NONOate, Nimodipine, Thapsigargin, THL, GDPβS, Calphostin C, Bicuculline, and SCH50911. These compounds were dissolved in water except 8-CPT, 2-AG, AM251, THL, nimodipine, and thapsigargin that were dissolved in dimethyl sulphoxide.

### Data analysis

The data were analyzed with the Clampfit 10.2 software (Molecular Devices) and the last 5 min of recording were used to estimate the changes in synaptic efficacy relative to the baseline. For the PPR experiments, two EPSPs were evoked for 30 s at the baseline frequency at the beginning of the baseline recording, 40 ms apart, and again 30 min after the end of the pairing protocol. The PPR was expressed as the slope of the second EPSP divided by the slope of the first EPSP. Coefficient of variation (CV) analysis was done on EPSP slopes^10^. Graphs were made using Sigmaplot 11.0.

### Statistical analysis

Shaphiro–Wilk normality and equal variance tests, with a confidence interval of 95% were performed before the statistical comparisons. For comparisons between two groups paired or unpaired Student’s test were used as appropriate. For Multiple comparisons with the same control, one-way analysis of variance followed by Holm–Sidak post hoc test was used. The data are expressed as the mean ± S.E.M. and *p* values <0.05 were considered significant. *p < 0.05, **p < 0.01, ***p < 0.001. *P* values are included in Supplementary Data 2.

### Reporting summary

Further information on research design is available in the Nature Research Reporting Summary linked to this article.

## Supplementary information

Supplementary Information

Peer Review File

Supplementary Data 1

Supplementary Data 2

Reporting Summary

## Data Availability

The data that support the findings of this study are available from the corresponding author upon reasonable request. Source data are provided with this paper.

## Source data

Source Data
