# Supplementary material for: Astrocyte-mediated switch in spike timing-dependent plasticity during hippocampal development
Source: Nat Commun. 2020 Sep 1;11:4388. doi: 10.1038/s41467-020-18024-4 (PMC7463247; doi:10.1038/s41467-020-18024-4)
Supplement: Supplementary file 1 — Supplementary Information [file 41467_2020_18024_MOESM1_ESM.pdf]

Astrocyte-mediated switch in spike timing-dependent plasticity during hippocampal development

Rafael Falcón Moya et al.,

Supplementary Figures

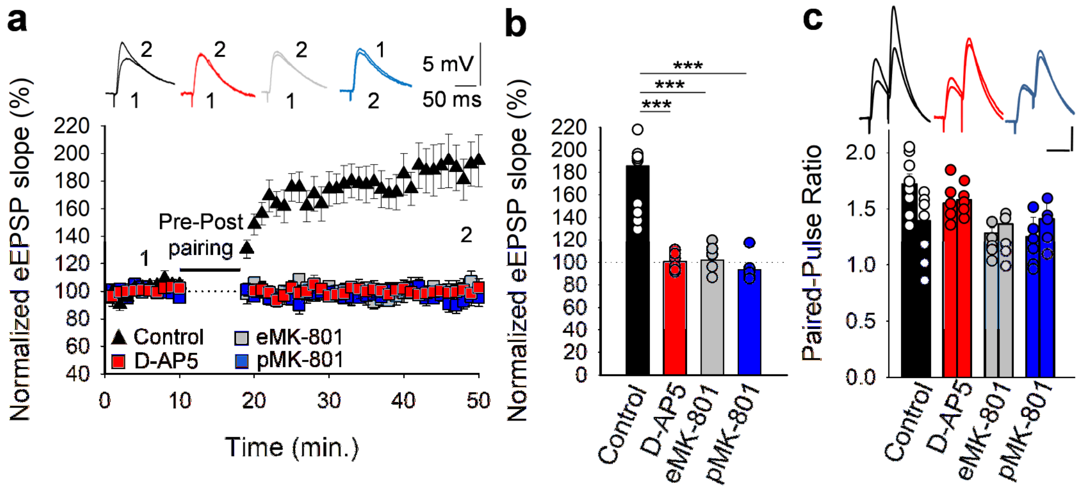

Supplementary Figure 1. Pre-Post postsynaptic t-LTP requiring NMDA receptor activation is present at P35-42 in the CA1 region of the hippocampus. (a) Pre-before-post, pairing protocol induced t-LTP in slices from P35-42 mice. The EPSP slopes monitored in the control condition (black triangles, n = 12) and in the presence of bath applied D-AP5 (50 µM, red squares, n = 6) and MK-801 (500 µM-1 mM, grey squares, n = 6) and with MK-801 loaded into the postsynaptic neuron (1 mM, blue squares, n = 6) are shown. Traces show the EPSP before (1) and 30 minutes after (2) pairing. **(b)** Summary of the results. **(c)** The paired-pulse ratio remained constant before and after t-LTP. Inset: Examples of paired-pulse facilitation traces during baseline and 30 minutes after induction of t-LTP in control slices (black, n = 12) and in slices treated with D-AP5 (red, n = 6) or MK-801 (blue, n = 6). Scale bars: 2 mV, 100 ms *** $p < 0.001$, one way ANOVA + Holm-Sidak. The error bars represent the S.E.M. For detailed PPR values see Supplementary Table 1.

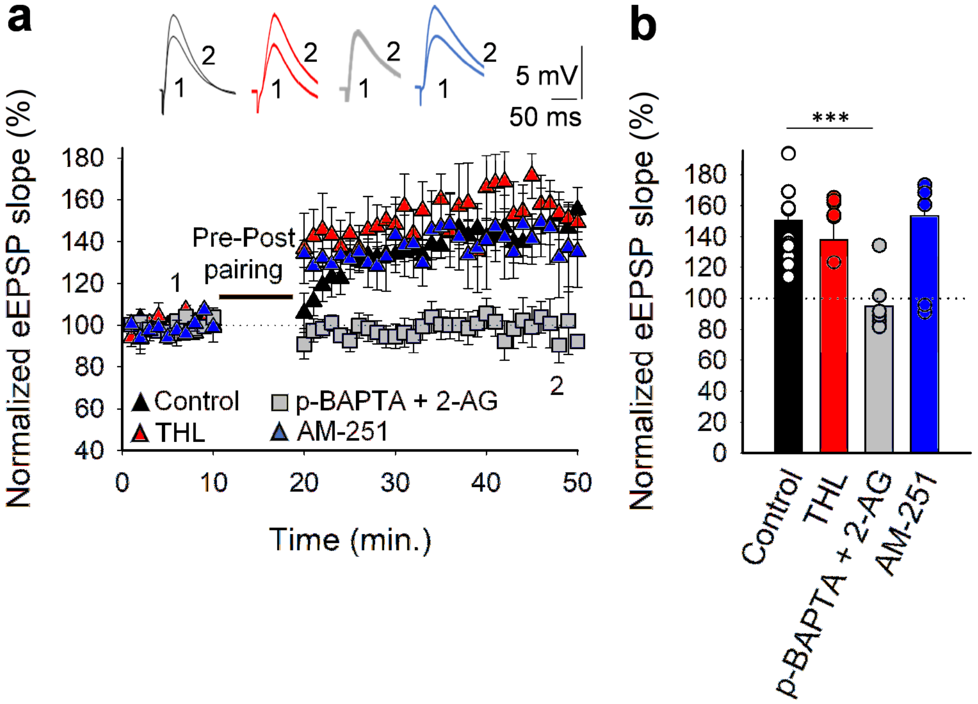

Supplementary Figure 2. Post-pre t-LTP does not require endocannabinoids nor CB_1 receptors. (a) The EPSP slopes monitored in the control condition (black triangles, n = 12), in the presence of THL (5 µM, red triangles, n = 6), of 2-AG (5 µM, with BAPTA loaded into the postsynaptic cell, grey squares, n = 10) and in the presence of AM-251 (3 µM, blue triangles, n = 6) are shown. Traces show the EPSP before (1) and 30 minutes after (2) pairing for each condition. **(b)** Summary of the results. *** $p < 0.001$, one way ANOVA + Holm-Sidak. The error bars represent the S.E.M.

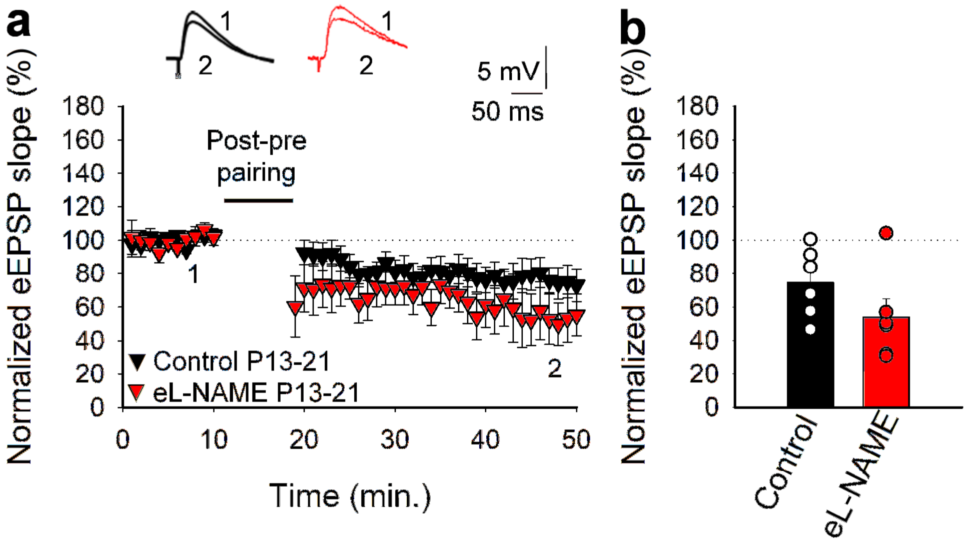

Supplementary Figure 3. Nitric oxide (NO) is not necessary for t-LTD at P13-21. **(a)** The EPSP slopes monitored in the control condition (black triangles, n = 6) and in the presence of L-NAME (100 μM, red triangles, n = 6) are shown. Traces show the EPSP before (1) and 30 minutes after (2) pairing for each condition. **(b)** Summary of the results. The error bars represent the S.E.M.

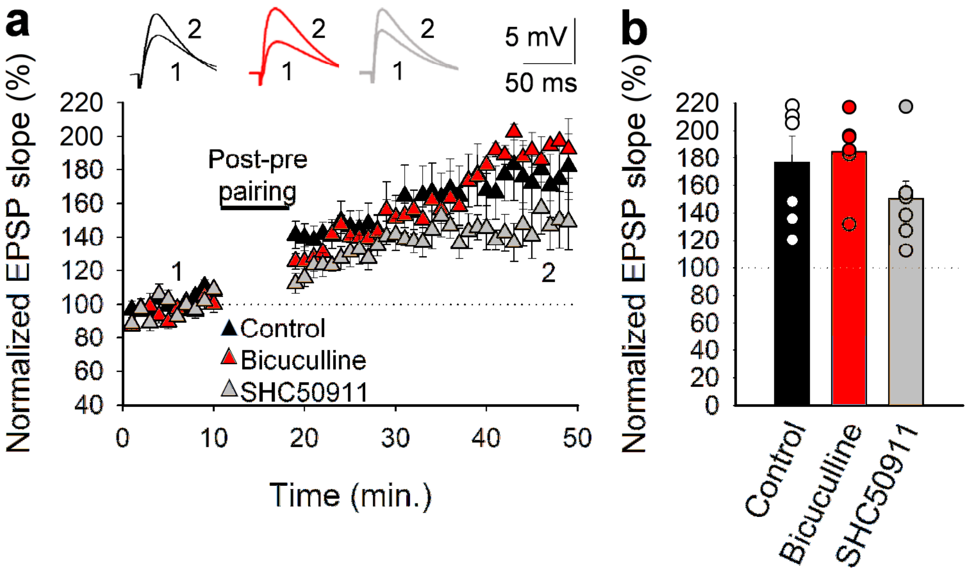

Supplementary Figure 4. The developmental switch from t-LTD to t-LTP does not involve GABAergic inhibition. (a) The switch to t-LTP with maturation is not due to the activation of $GABA_A$ or $GABA_B$ receptors, as t-LTP was not affected in the presence of bicuculline (20 µM, red triangles, n = 7) or SHC50911 (50 µM, grey triangles, n = 7) when compared to control t-LTP (black triangles, n = 6). The insets show the EPSP before (1) and 30 min after (2) pairing. **(b)** Summary of the results where the error bars represent the S.E.M.

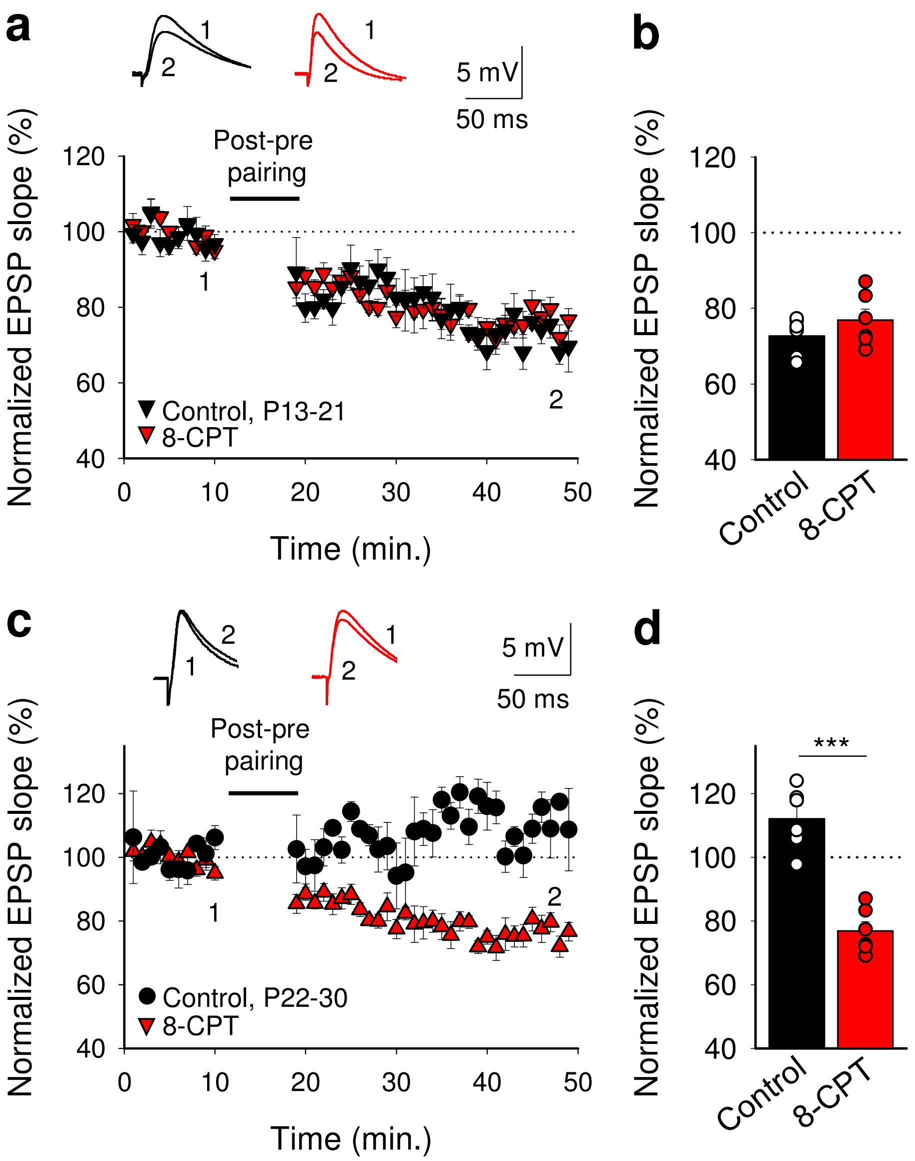

Supplementary Figure 5. A_1R are not necessary for t-LTD at P13-21. (a) The EPSP slopes monitored in the control condition (black triangles, n = 6), in the presence of 8-CPT (2 µM, red triangles, n = 6) are shown at P13-21. Traces show the EPSP before (1) and 30 minutes after (2) pairing for each condition. **(b)** Summary of the results. **(c, d)** A_1R are involved in the loss of t-LTD at P22-30. **(c)** The EPSP slopes monitored in the control condition (black circles, n = 6), in the presence of 8-CPT (red triangles, n = 6) are shown at P22-30. Traces show the EPSP before (1) and 30 minutes after (2) pairing for each condition. **(d)** Summary of the results. ***p < 0.001, two-sided Students´s t-test. The error bars represent the S.E.M.

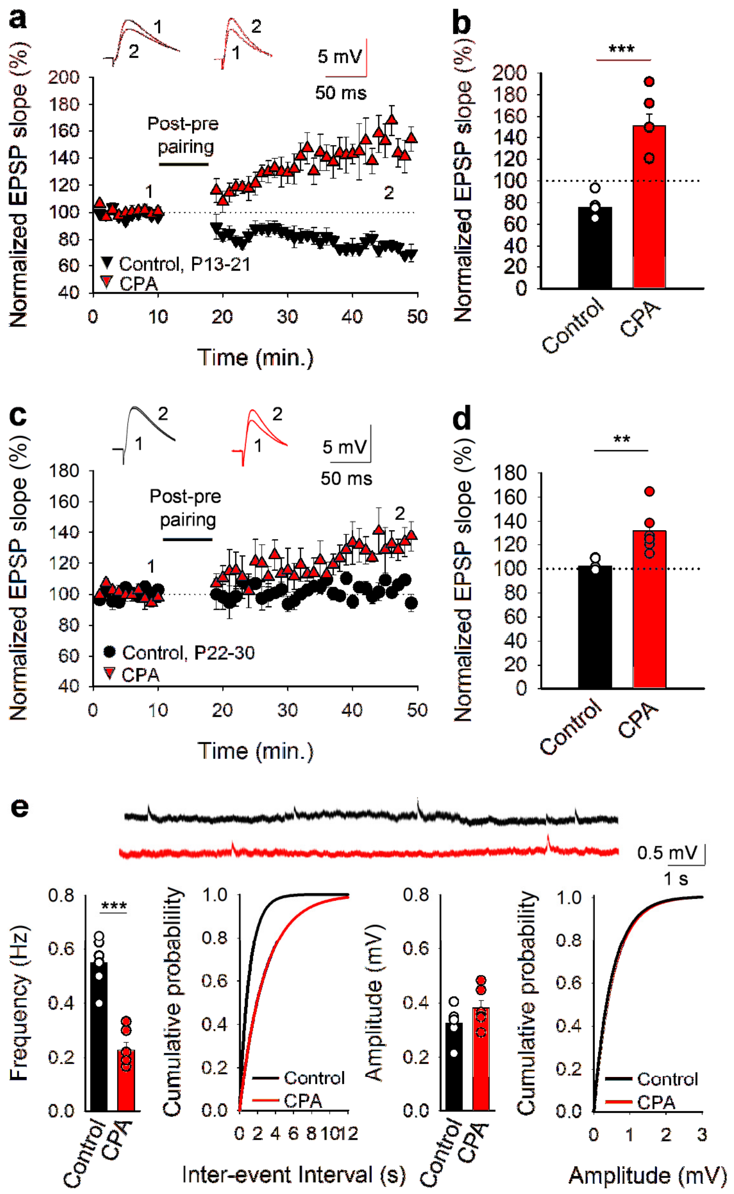

Supplementary Figure 6. An increase in presynaptic A_1R mediated-inhibition converts t-LTD at P13-21 and no t-LTD at P22-30 in t-LTP. (a) When A_1Rs are activated by 50 nM CPA at P13-21, a post-pre protocol induced t-LTP. The EPSP slopes monitored in the control condition (black triangles, n = 7) and in the presence of bath applied CPA (red triangles, n = 7) are shown. Traces show the EPSP before (1) and 30 minutes after (2) pairing. **(b)** Summary of the results. *** $p < 0.001$, two-sided Student's *t*-test **(c)** When A_1Rs are activated by 50 nM CPA at P22-30, a post-pre protocol induced t-LTP. The EPSP slopes monitored in the control condition (black circles, n = 6), in the presence of CPA (red triangles, n = 6) are shown. Traces show the EPSP before (1) and 30 minutes after (2) pairing for each condition. **(d)** Summary of the results. ** $p < 0.01$, two-sided Student's *t*-test. **(e)** Miniature EPSP during the baseline (black, n = 6) and after exposing neurons from slices from P13-21 animals to CPA (red, n = 6) in the presence of TTX (500 nM) are shown. Histograms and cumulative graphs show that, at P13-21, CPA decreases the mEPSP frequency but it does not affect the mEPSP amplitude indicating a presynaptic action of CPA. *** $p < 0.001$, two-sided Student's *t*-test. The error bars represent the S.E.M.

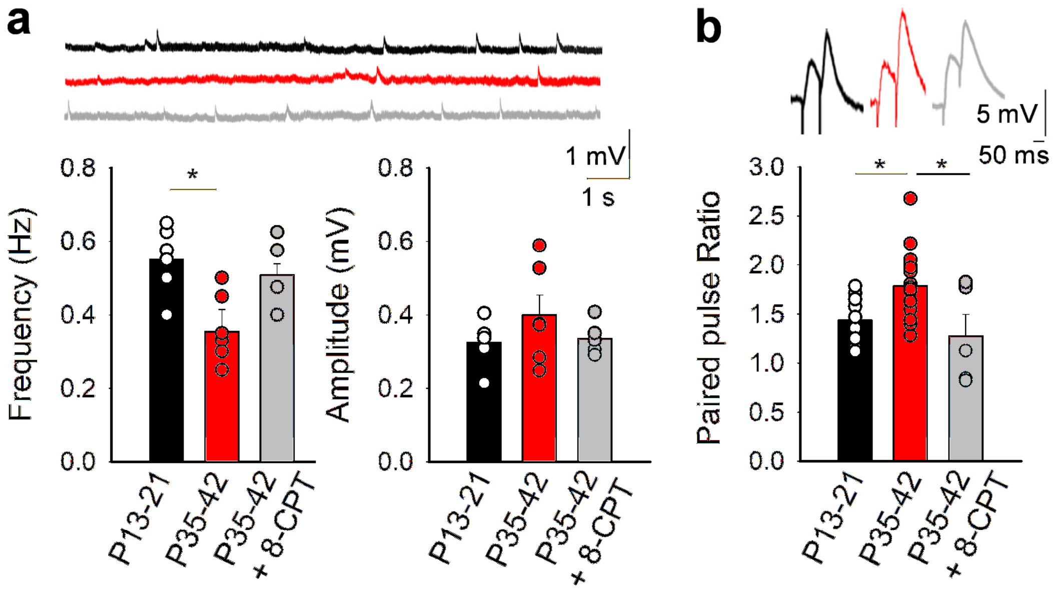

Supplementary Figure 7. Excitatory spontaneous and evoked activity decreases with maturation through a mechanism that involves adenosine A_1R activation. (a) mEPSP frequency decreases with maturation, an effect that is prevented by the antagonism of A_1Rs with 8-CPT. Traces show the mEPSP at P13-21 (black, n = 6) and P35-42 in control slices (red, n = 6), and at P35-42 in slices treated with 8-CPT (grey, n = 6). The frequency of the mEPSPs clearly diminishes with maturation but not when the slices were treated with 8-CPT. No changes in the amplitude were observed as development proceeds. * $p < 0.05$, two-sided Student's *t*-test. **(b)** Paired-pulse ratio of evoked responses increases with maturation, an effect that is prevented by the antagonism of A_1R with 8-CPT. Traces show evoked eEPSP (paired responses at 40 ms interval) at P13-21 (black, n = 11) and at P35-42 in control slices (red, n = 19), and at P35-42 slices treated with 8-CPT (n = 5). * $p < 0.05$, two-sided Student's *t*-test. The error bars represent the S.E.M. For detailed PPR values see Supplementary Table 1.

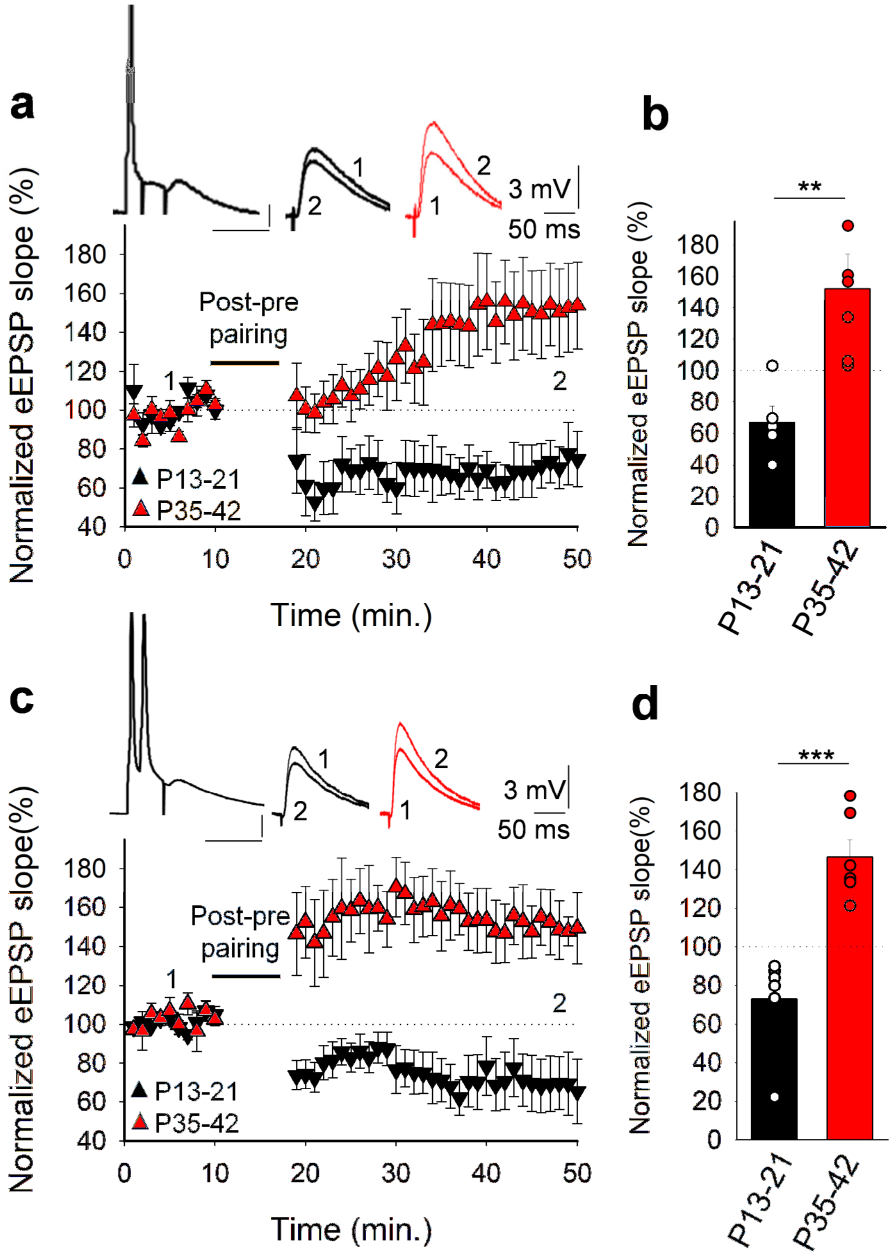

Supplementary Figure 8. A switch from t-LTD to t-LTP is present using different STDP protocols. (a, b) Pairing (100 times, 0.2 Hz) a single postsynaptic spike with a burst of two eEPSP at 100 Hz induces a switch from t-LTD (at P13-21) to t-LTP (at P35-42). **(a)** The EPSP slopes monitored at P13-21 (black triangles, 67 ± 10%, n = 6), and at P35-42 (red triangles, 152 ± 17%, n = 6) are shown. Traces show the EPSP before (1) and 30 minutes after (2) pairing. **(b)** Summary of the results. ** $p < 0.01$, two-sided Student's *t*-test.(**c, d)** Pairing (100 times) 2 postsynaptic spikes at 100 Hz with single EPSP induces a switch from t-LTD (at P13-21) to t-LTP (at P35-42). **(c)** The EPSP slopes monitored at P13-21 (black triangles, 72 ± 10% n = 6), and at P35-42 (red triangles, 146 ± 9% n = 6) are shown. Traces show the EPSP before (1) and 30 minutes after (2) pairing. **(d)** Summary of the results. ** $p < 0.01$, *** $p < 0.001$, two-sided Student's *t*-test. The error bars represent the S.E.M.
