# Supplementary material for: Astrocyte-mediated switch in spike timing-dependent plasticity during hippocampal development
Source: Nat Commun. 2020 Sep 1;11:4388. doi: 10.1038/s41467-020-18024-4 (PMC7463247; doi:10.1038/s41467-020-18024-4)
Supplement: Supplementary file 5 — Reporting Summary [file 41467_2020_18024_MOESM5_ESM.pdf]

nature research

Corresponding author(s): Antonio Rodríguez-Moreno

Last updated by author(s): Jul 28, 2020

Reporting Summary

Nature Research wishes to improve the reproducibility of the work that we publish. This form provides structure for consistency and transparency in reporting. For further information on Nature Research policies, see our Editorial Policies and the Editorial Policy Checklist.

Statistics

For all statistical analyses, confirm that the following items are present in the figure legend, table legend, main text, or Methods section.

n/a	Confirmed	
☐	☒	The exact sample size (*n*) for each experimental group/condition, given as a discrete number and unit of measurement
☐	☒	A statement on whether measurements were taken from distinct samples or whether the same sample was measured repeatedly
☐	☒	The statistical test(s) used AND whether they are one- or two-sided<br>*Only common tests should be described solely by name; describe more complex techniques in the Methods section.*
☒	☐	A description of all covariates tested
☐	☒	A description of any assumptions or corrections, such as tests of normality and adjustment for multiple comparisons
☐	☒	A full description of the statistical parameters including central tendency (e.g. means) or other basic estimates (e.g. regression coefficient) AND variation (e.g. standard deviation) or associated estimates of uncertainty (e.g. confidence intervals)
☐	☒	For null hypothesis testing, the test statistic (e.g. *F*, *t*, *r*) with confidence intervals, effect sizes, degrees of freedom and *P* value noted<br>*Give P values as exact values whenever suitable.*
☒	☐	For Bayesian analysis, information on the choice of priors and Markov chain Monte Carlo settings
☒	☐	For hierarchical and complex designs, identification of the appropriate level for tests and full reporting of outcomes
☒	☐	Estimates of effect sizes (e.g. Cohen's *d*, Pearson's *r*), indicating how they were calculated

Our web collection on statistics for biologists contains articles on many of the points above.

Software and code

Policy information about availability of computer code

Data collection	pClamp 10.2, Clampex 10.2 (Molecular Devices)
Data analysis	pClamp 10.2, Clampfit 10.2 (Molecular Devices). Data collected with these software was further analyzed and plotted using SigmaPlot 11.0

For manuscripts utilizing custom algorithms or software that are central to the research but not yet described in published literature, software must be made available to editors and reviewers. We strongly encourage code deposition in a community repository (e.g. GitHub). See the Nature Research guidelines for submitting code & software for further information.

Data

Policy information about availability of data

All manuscripts must include a data availability statement. This statement should provide the following information, where applicable:

- Accession codes, unique identifiers, or web links for publicly available datasets
- A list of figures that have associated raw data
- A description of any restrictions on data availability

Experimental data supporting the findings of this study are available upon reasonable request from the corresponding author

Field-specific reporting

Please select the one below that is the best fit for your research. If you are not sure, read the appropriate sections before making your selection.

☒ Life sciences ☐ Behavioural & social sciences ☐ Ecological, evolutionary & environmental sciences

For a reference copy of the document with all sections, see nature.com/documents/nr-reporting-summary-flat.pdf

Life sciences study design

All studies must disclose on these points even when the disclosure is negative.

Sample size	The samples sizes used are similar to those generally used in the field what are the results of the use power analysis on preliminary results using alpha= 0.05 and beta = 0.2
Data exclusions	No data were excluded from analysis
Replication	The exact number of replications for each experiment is detailed in text and figures. All attempts of replication were sucessfull.
Randomization	Animals were allocated randomly.
Blinding	Analysis was not performed blind to the condition of the experiments, but the same and consistent criteria were applied for data adquisition and analysis. Experimenters were blinded to experimental conditions when applicable (in genotype of animals during electrophysiological experiments)

Reporting for specific materials, systems and methods

We require information from authors about some types of materials, experimental systems and methods used in many studies. Here, indicate whether each material, system or method listed is relevant to your study. If you are not sure if a list item applies to your research, read the appropriate section before selecting a response.

Materials & experimental systems

n/a	Involved in the study	
☒	☐	Antibodies
☒	☐	Eukaryotic cell lines
☒	☐	Palaeontology and archaeology
☐	☒	Animals and other organisms
☒	☐	Human research participants
☒	☐	Clinical data
☒	☐	Dual use research of concern

Methods

n/a	Involved in the study	
☒	☐	ChIP-seq
☒	☐	Flow cytometry
☒	☐	MRI-based neuroimaging

Animals and other organisms

Policy information about studies involving animals; ARRIVE guidelines recommended for reporting animal research

Laboratory animals	Mus Musculus, C57Bl/6J, males and females were used, age: P13-P42, some experiments performed in dnSNARE mutants. Anaimals were kept at 18-24 °C at 40–60% humidity, and with full availability of food and water.
Wild animals	This study does not involve wild animals
Field-collected samples	No fiel-collected samples were used in this study
Ethics oversight	All protocols and procedures were performed according to the European Union Directive 2010/63/EU regarding the use of animals for scientific research. Experiments were approved by the Ethics Committee of University Pablo de Olavide and by the Ethics Commitee of the Andalusian Government.

Note that full information on the approval of the study protocol must also be provided in the manuscript.

April 2020
