# Supplementary material for: Astrocyte-mediated switch in spike timing-dependent plasticity during hippocampal development
Source: Nat Commun. 2020 Sep 1;11:4388. doi: 10.1038/s41467-020-18024-4 (PMC7463247; doi:10.1038/s41467-020-18024-4)
Supplement: Supplementary file 2 — Peer Review File [file 41467_2020_18024_MOESM2_ESM.docx]

**Reviewer comments, first version:**

Reviewer #1 (Remarks to the Author):

The authors show that a post-pre pairing protocol that induces LTD at P13-18, induces LTP at P35-42, and make the case that this is due to reducing of release probability over this period, due to tonic glial adenosine release, coupled with phasic glial glutamate release to assist in presynaptic potentiation.

One major issue is the exclusive focus on presynaptic locus of t-LTP. They provide some evidence that there is an increase in presynaptic release probability after t-LTP (lowered PPR, lowered failure rate) but appear to perform no experiment whatsoever to address whether there is an post-synaptic component to t-LTP (is there an increase in Q? in mEPSC size? in unsilencing of synapses?). Thus the assertion that this form of LTP is exclusively presynaptically expressed is not justified.

The key claim in this study is that astrocytes cause a developmental switch in spike time dependent plasticity from LTD to LTP. However, none of the astrocytic interventions at P35-42 convert LTP back to LTD, LTP is simply inhibited . Thus, there must be other changes that are needed for this switch to occur not involving astrocytes. In a similar vein, the authors do not check the effect of any of the astrocytic interventions on P13-21 LTD, so even a differential role of astrocytes on plasticity at the two developmental stages is not proven. Overall the study is probably best suited to a specialist neurophysiology journal.


Reviewer #2 (Remarks to the Author):

To the authors;

Prius-Mengual et al. find a switch in CA3-CA1 synaptic plasticity rules associated with anticausal spike-timing, which early in development elicits presynaptic LTD, but later on, owing to astrocyte-mediated adenosine signalling, elicits a novel form of presynaptic LTP that likely depends on calcium influx from postsynaptic L-VGCCs and internal stores, NO signalling, and activation of presynaptic mGluR5 by astrocytic glutamate release. Overall, the paper is well controlled, and has the potential to be of interest, drawing upon themes of plasticity, development, critical periods, and astrocytes.
I do have some concerns, which I believe if addressed, could greatly enhance the impact of the work.

Major Concerns:
1) Concern: The study is somewhat limited in scope. On one hand, it is amongst the few to extend our understanding of plasticity to beyond the developmental time period commonly investigated by saying that the plasticity rules do change in interesting ways with age, which certainly will be important for understanding network-level computations in the adult. However, on the other hand, the scope of this developmental change is not fully explored. The authors only look at a single spike timing interval, which gives an incomplete picture of how STDP rules change with development. I feel it would considerably increase the impact of the paper if the authors explored in greater detail the timing dependency of plasticity (ie construct a full STDP curve), so as to better understand synaptic changes across a range of spike timings. Without such information it is impossible to say whether t-LTD is truly abolished in the more mature rodent, or whether the STDP rules are just broadened or
shifted along the time axis, which again limits the scope of the authors’ findings. It is also difficult to say what exactly are the timing requirements for the t-LTP they describe, leaving the characterization of the novel plasticity they describe incomplete. Clarifying these points are important.

Interestingly, work from Huibert Mansvelder’s lab, and in particular the study Guilherme et al., 2010 (Frontiers in Neuroscience) shows that the STDP curve is indeed broadened at adult human hippocampal synapses (see their Figure 6) such that positive timing intervals yield LTP, short negative timing intervals (<80ms) also yield LTP, and only at long negative intervals (> 80ms) does LTD become apparent. This is of course very different from what was previously known about STDP in rodent hippocampus, which was assessed in developing animals. The general thinking, therefore, has been that human synapses are different from rodent ones. However, the findings of Guilherme et al., 2010 are not inconsistent with what the authors now show in their study, in which short negative timing intervals produce LTP in the more mature rodent. The question is what happens at longer negative timing intervals, would they also see LTD as in the human? If so, it would mean that synaptic learning rules
may be conserved across species. A more through exploration on the timing dependence of plasticity in the more mature rodent would therefore be very valuable to both the plasticity community, and to neuroscientists who are interested in the consequences of network-level learning rules in older animals, in which most in vivo work is conducted.

Suggestion for address: The authors should broaden the scope of their study by assessing the impact of a range of spike timings on plasticity in the more mature rodent (P35-P42). I would follow the example of Guilherme et al., 2010 (Figure 6) in which intervals of -140 to +20 are sampled in one set of experiments, and compare findings with this study. Incidentally, to date I do not believe anyone has constructed an STDP curve of presynaptic LTP (which could be done using concurrently collected PPR data). It would be a very useful data set for those interested in presynaptic plasticity.

2) Concern: Related to my second concern, the study is limited in its exploration of plasticity rules. Just because the authors’ particular STDP protocol shows an interesting developmental switch, does not imply that all STDP protocols will show similar effects. For example, given the reduction in release probability is important for promoting t-LTP over t-LTD, what would happen if short bursts of CA3 input (2 spikes at 100-200Hz; which are quite common in vivo) were anticausally paired with postsynaptic activity instead of a single presynaptic spike? Such presynaptic bursts would surely mobilize more glutamate release, which may, for example better recruit preNMDAR signalling to induce preLTD instead of preLTP. Rather than particular forms of plasticity appearing or disappearing then, developmental maturation, by altering for example by altering the strength of various molecular pathways, may lead to an alteration in the threshold signalling requirements for particular forms of
plasticity. When assessed by a single plasticity protocol this may give the illusion of the disappearance/appearance of plasticity forms.

Suggestion for address: It would greatly strengthen the paper’s conclusions to assess how physiological changes in glutamate release probability impact plasticity rules (ie STDP pairing of presynaptic bursts with postsynaptic spikes). If burst pairing abolishes or reduces preLTP this would provide very strong support the author’s model, in which preLTP may be driven by a decrease in transmitter release probability. This finding may also suggest that preLTD may not be fully abolished, but rather the threshold requirements for its activation has changed with development. The experiment would also greatly extend the authors findings into understanding how physiological changes in glutamate release impact plasticity rules, greatly enhancing the scope of the paper. I would therefore strongly recommend the authors do this experiment, but leave it only as a suggestion. At the very least they should briefly discuss the limitation of their study, and in particular how different
patterns of pre/post activity may have differential developmental-dependent outcomes. preLTD may therefore not be abolished with development, but its rules for inductions may be changed.

3) Concern: The study is conducted at a non-physiological (room) temperature. This is a principle issue when studying presynaptic plasticity, as presynaptic function is particularly sensitive to non-physiological temperatures, as is both NMDAR and NO signalling. For example, activity-dependent, and NO-dependent enhancement of presynaptic has been shown to depend on axonal excitability, but only at sub-physiological temperatures (McNaughton et al, 1994, PNAS). Indeed, the impact of NO on facilitating presynaptic function is temperature dependent and in one report, seen at room temperature but not at physiological temperatures (Williams et al., 1993, Neuron). Moreover, given that temperature is known to alter the distribution of release probabilities within the tissue (e.g Hardingham & Larkman, 1998; Volgushev et al., 2004), and the authors effect seems to be dependent on adenosine-mediated changes in release probability, there is a concern that their findings are limited to
non-physiological functions. With the growing interest of exploring and understanding in vitro phenomenon in vivo, it is important to keep experimental conditions as physiological as possible to ensure lasting impact of the work.

Suggestion for address: The authors should assess whether their preLTP persists at physiological/near physiological temperatures (>30C). In principle this could be addressed if they conduct experiments suggested in Concern #1 at physiological/near physiological temperatures (>30C).

Minor concerns/suggestions:

1) The authors do not show individual data points on their bar graphs, and so it is difficult to gauge the variance and therefore quality of the data. Transparency in data is very important, and individual data can be very useful to modellers. For PPR measures, seeing this variance is useful since basal PPR may very well influence the magnitude and direction of the plasticity. Ideally, to help the reader understand the data the authors should plot individual data points on top of their bar graphs; where paired (ie in Figure 1E) the points should be connected by a line. Alternatively, the authors should provide the value of the points in a supplementary table.

2) Related to the above, given the authors suggest that changes in transmitter release efficacy may underlie the developmental switch of t-LTD to t-LTP have the authors done a correlation between initial PPR and synaptic efficacy following STDP? This would be nice to include.

3) The authors only show PPR data in Figure 1E, but not throughout their manipulations; however, it would be important to see that changes in PPR are following expected changes in synaptic efficacy; this would help strengthen the manuscripts conclusions. Where the authors do have PPR (and even the failure analysis data) for their manipulations they should show it, or at the very least quote in text or provide a supplementary figure/table.

4) The authors do individual t-tests for their statistics. Whilst their can be some statistical justification for doing this (especially if declared a priori), there is still a risk of inflating the family-wise error rate. The authors should really consider using an ANOVA (in the case of normally distributed data, or an equivalent model to assess group statistics) and post hoc tests to assess significance. Looking at the authors data I hardly think this will change the conclusions of the study, especially since all the data tell a parsimonious story.

5) In Figure 2E there looks to be run-up in the baseline, in which case the LTP looks like a simple continuation of this run up. Is this baseline run-up genuine or just unfortunate sampling? Could it be something in the vehicle? If it is the case, it would be important to repeat the experiment without pairing to see what the run-up is over time, and to redo stats against this. Also can the authors please make clear in the methods what vehicles were used and what solvent reagents were dissolved in (ie DMSO and respective concentration).

6) Do the authors have a positive control for the use of GDPBS (Figure 6E,F) and of THL (Fig S2). These drugs show no effect but how do they know its working? Can the authors cite a previous paper using these drugs successfully with the same parameters used in this study? The use of GDPBS is their only evidence that the mGluR5s are presynaptic so it would be good to have some indication that this manipulation is working.

7) The authors show that nimodipine, thapsigargin, and L-NAME into the postsynaptic neuron blocks t-LTP, giving the impression that postsynaptic L-VGCCs, internal stores, and NO synthase are involved; but how can they be sure these drugs are only acting postsynatpically and not impacting closely situated astrocytic/presynaptic terminals via diffusion. These drugs show some lipid solubility. Do the authors have a control or citation showing that these drugs would only act in the postsynaptic compartment if intracellular dialyzed. If not it would be important for the authors to clarify in the text that these drugs may be acting on non-postsynaptic targets; as such the authors should moderate their conclusions that postsynaptic L-VGCC and internal stores are required.

If postsynaptic internal stores are important, how are they being recruited if not by mGluRs. There is evidence that postsynaptic VGCCs can do this (e.g Dittmer et al., 2017 Cell Reports, Johenning et al., 2015 Plos Bio -Fig 6). The authors should cite and very briefly discuss.

8) It would be really nice if the authors could include a figure of their proposed mechanism. This is especially useful for readers not in the immediate speciality, and for those who ‘figure read’ manuscripts, which I am afraid is quite often the case these days.

9) I would suggest avoiding the use ‘fluctuation analysis’ (an unnecessary jargon in general), which really can mean a number of forms of analyses and so it is not very informative and would be distracting to general reader. I would just refer to it as failure rate or response failures (ie in the abstract ‘t-LTP was expressed presynaptically as witness by a decrease in response failures and the paired pulse ratio’).

10) In the methods the authors suggest that ‘during the experiments, changes in Vm (1-3 mV) were corrected by imposing continues current’; is this wise? How do you know the changes were not due to offset/electrical drift?

11) Could the authors include the distance from the soma they placed the stimulating electrodes in the Methods section; useful to know how far up the dendritic tree they are, since this impacts plasticity rules, backprop efficiency, the effect of dialysis, etc, and would be useful to know for interpreting the results.

12) In the methods the authors write ‘Glutamate was included in the external solution,’ which makes it seem that you bath applied it. I think you mean to say ‘Glutamate was dissolved in external solution’

13) The authors refer to astrocytic adenosine-mediated activation of presynaptic A1 receptors as ‘inhibition’ (ie subtitle in Results section). The use of inhibition and a stand-alone word is often associated with GABAergic transmission, so, in my opnion, it would be better to use another word or to at least say ‘inhibition of presynaptic release’ to avoid confusion.

14) I do not think it’s necessary to cite Cajal’s Croonian Lecture in reference to experience-dependent plasticity.

15) The authors miscite ref 24 in the statement ‘….the release of Ca2+ from intracellular stores is also required to induce t-LTD[11] and LTP at hippocampal synapses[24]; ref 24 – Padamsey et al (elife, 2017) did not show this. Also the sentence is misleading because stores are not always required for all forms of plasticity (for example, see recent review from Emptage lab: Padamsey et al., 2018 – The Neuroscientist) the authors should clarify this in text. Similarly, the authors write ‘the requirement of mglurs for presynaptic preLTP has previously defined’, however again mGluRs are not always necessary for all forms of LTP (nor all forms of preLTP); the authors should clarify this in text.

16) The authors cite refs 30-35 in support of ‘there is evidence that calcium influx though L-type calcium channels could participate in NO synthesis and/or its release from postsynaptic neurons. Ref 31-33 are not strong support of this; 32-33 do not show this at all. Reference 30 also claims specific coupling of NO production to NMDAR, and also use non-physiological stimuli to make their point. Reference 34 uses highly non-physiological stimuli to drive NO synthesis via L-VGCC activation (high K+ plus the LVGCC channel activator Bay K 8644). The particularly relevant citations for this statement should be 35 – Pigott et al., who first show L-VGCC dependent potentiation is also NO sensitive at CA3-CA1 synapses, and 24- Padamsey et al. (elife) who first show that physiological activation of L-VGCCs is sufficient to drive NO release from CA1 dendrites, which in turn increases release probability.

17) The authors write in the discussion ‘There is almost no information regarding the possible behavioural influence of presynaptic LTP’. Choi et al 2018 (Science) very beautifully show presynaptic (and postsynaptic) changes within the synaptic engram associated with context-dependent fear conditioning. Whilst it is not causal, it would be important to mention that preLTP is associated with learning and memory in vivo.

18) The authors write they found a similar switch in plasticity using a 15Hz 1s LTD protocol, which was antagonized by 8-CPT. The authors should show this data as supplemental information as it supports their study. Otherwise the author should remove this sentence; without data the reader cannot evaluate the statement. On this note, the authors should cite

19) To support their model, have the authors seen if addition of adenosine agonists can prematurely trigger preLTP in younger animals? This would be good support for their model (but is by no means required).

20) The authors mention in the discussion ‘our findings expand the repertoire of presynaptic LTPs.’ It would be good to give readers references to these different forms and/or to mention them.

Sincerely,

Zahid Padamsey


Reviewer #3 (Remarks to the Author):

Prius-Mengual et al. describes a novel form of NMDAR-independent timing-dependent long-term potentiation (t-LTP) at CA3 to CA1 synapses. The group previously showed that, at CA3-CA1 synapses, tLTD disappears at later stages (Pérez-Rodríguez 2019), and this study follows up on those findings. Using the analysis of failure rates and PPR change, they first demonstrated that this new form of t-LTP is expressed presynaptically. They then showed that its induction requires the activation of presynaptic mGlu5Rs but not NMDARs like for tLTD at earlier stages, and that postsynaptic Ca2+ and retrograde NO signalling from the postsynaptic neuron are required to induce this presynaptic t-LTP. Finally, they demonstrate that this form of presynaptic t-LTP requires astroglial signaling, which developmentally dampen neuronal glutamate release via the release of ATP/adenosine enabling the t-LTD-to-t-LTP switch. In summary, this t-LTP thus depends on mGlu5R, L-type calcium channels, nitric oxide,
and involves adenosine and glutamate release from surrounding astrocytes.

This manuscript is clearly written (although the Discussion is a bit repetitive and too long), it has novel findings, the figures are clear, and the data largely support the findings that are drawn. The science is solid and entirely believable, but the statistics somewhat flawed. We are, however, not sure that this manuscript is of sufficient novelty for publication in Nature Communications. The manuscript would be strengthened considerably if the below points were addressed in full.

MAJOR POINTS:

1) Experiments were carried out at room temperature instead of physiological temperatures. To ensure biological relevance, authors should clarify whether the forms of plasticity induced in their experiments would also be induced if the experiments were carried out closer to physiological temperature.

2) The authors should clarify which forms of plasticity is induced in P31 - P34 mice.

3) Please clarify the mechanism of action for thapsigargin. It inhibits sarco-endoplasmic reticulum Ca2+-ATPase which actively sequesters Ca2+ in stores. Therefore, there is a possibility that intracellular levels of Ca2+ remains unchanged, or even increases, with the application of thapsigargin. If so, then the conclusion that it is Ca2+ release that is required for this form of t-LTP would be challenged.

4) I believe the dnSNARE mouse line works with a tetOff system. If so, please clarify in Methods section whether and/or when the dnSNARE mice were supplied with doxycycline to turn off transgene expression. This is important because if gliotransmission is always blocked then these mice would not be a good comparison with the fluoroactate and a-BAPTA treatment as they might exhibit different forms of plasticity altogether, especially since this form of t-LTP is supposed to be brought about by atrocyte-released adenosine through maturation.

5) Novelty is potentially not high enough. Even though the study is well-designed and the paper well-written, this study probably does not provide enough conceptual advance to make it suitable for publication in Nature Communications. This is because many of the ideas suggested here have already been proposed:
- developmental switch of the sign of plasticity (post vs presynaptic NMDAR for tLTD; Corlew 2007, Larsen Philpot NN 2014)
- involvement of mGluRs in presynaptic plasticity
- astrocyte involvement in the control of synaptic plasticity. Panatier, 2006 showed that astrocytes contribute via D-serine to synaptic metaplasticity in the hippocampus. It was also shown that tLTD in neocortex (Min Nevian 2012) and t-LTP in hippocampus is controlled by astrocytic signalling.
- the developmental regulation of astrocytes: from Foncelle 2018. “In the developing hippocampus a presynaptic tLTD at CA3-CA1 synapses requires D-serine signaling possibly released from astrocytes (Andrade-Talavera et al., 2016). Interestingly, the same STDP pairing protocol induces tLTP at later developmental stages suggesting the possibility that astrocytic coverage of neurons and modulation of STDP by gliotransmission may be developmentally regulated."
- astrocytes can decrease transmitter release by activating presynaptic A1 adenosine receptors (Pascual 2005; Serrano 2006; Todd 2010). Following their activation via mGluR5 during basal synaptic transmission, astrocytes can even increase the efficiency of transmission in CA1 pyramidal cells through activation of presynaptic adenosine A2A receptors (Panatier 2011).

6) The mechanism is not clear. “t-LTP involves enhanced inhibition due to astrocytic adenosine-mediated activation of presynaptic adenosine A1Rs”:
- does 8-CPT have any effect in P13-21 and P22-30 mice?
- would activating A1R by bath applying the A1R agonist CPA to dampen glutamate release probability (which could be verified by measuring a mEPSP frequency decrease) turn tLTD into tLTP in P13-21 mice, and no tLTD to t-LTP in P22-30 mice? This might require the glutamate puffs (as in Figure 4G) too, if astrocytes are not capable yet to release glutamate at these stages.
This experiment would confirm the switch being driven by the dampening of neuronal glutamate release by astrocytic ATP/Adenosine release.

7) The statistical analysis is flawed. Page 8, "A normality and equal variance test was performed", but it is not stated which precise tests were performed, e.g. the F test, a goodness of fit test, or something else. Also, there are multiple comparisons in every single figure (Figure 1-4, Figures S1-S4), yet no post-hoc correction for multiple comparisons is applied according to the Methods section. The appropriate procedure would be to test for homoscedastity, then apply the appropriate ANOVA (or nonparametric equivalent, if not normally distributed). If outcome suggests it at the 0.05 level, pairwise comparisons would be allowed, adjusted for unequal variance if need be, with outcomes corrected post-hoc for multiple comparisons, using e.g. Bonferroni Dunn. Instead, the authors go straight for the pairwise comparisons and never correct post hoc for multiple comparisons, which increases the risk for false positives considerably. Finally, the authors need to state the thresholds
for one, two, and three star significance levels in the Methods.

8) The present study is missing a theoretical framework to tie in the findings with. The findings are presented as just-so stories, e.g. p18 "there is a maturation associated shift from the involvement of NMDARs to mGluRs in hippocampal plasticity". It is never explained why Mother Nature would want it to be this way, or how this benefits brain functioning. Furthermore, pre-post LTP and post-pre LTP are post and presynaptically expressed, but the computational implications of this is never discussed. For example, a change in short-term dynamics upon induction of long-term plasticity will affect temporal coherence, see e.g. Abbott et al Science 1997 275:220-224, or Markram Tsodyks Nature 1996, but the same is not true for insertion of AMPARs, which would reduce signal to noise ratio, see e.g. Otmakhov Malinow Neuron 1993. Basically, expressing LTP pre or postsynaptically is not identical in terms of outcome even if the gain is changed by the same percentage. This should be
discussed in detail in the Discussion. The theoretical framework should be provided to the readership. In this context, I would say that sentences such as "adding to the repertoire of functional modulation and probably facilitating better control of synaptic transmission" (p19) are vague and not helpful to a reader seeking to better understand the implications of the present findings, chiefly because that statement is not specific.

9) Nitric oxide signalling is blocked but never elicited, so there is an asymmetry that does not support the interpretation for both need and sufficiency for NO signalling in this form of plasticity. Experiments with a nitric oxide donor should thus be carried out to warrant the conclusions drawn.

10) Page 16, "This glutamate presumably activates presynaptic mGluRs" (also see p26), why does the Glu have to come from glia? Why can it not come from the synapse itself, where mGluRs act as autoreceptors? The authors have not excluded this quite likely possibility.

11) Page 16, "We tested whether the probability of glutamate release decreases at CA3-CA1 synapses with maturation by measuring miniature EPSP", spontaneous release frequency is a poor measure of evoked release probability, e.g. see papers by Ege Kavalali showing that these forms of release do not rely on the same mechanisms. This should at least be discussed, but ideally addressed experimentally.

MINOR POINTS:

1) For consistency, THL data should be included in Figure S2A. Similarly, it would be clearer for readers if fluoroacetate and 8-CPT data were included in Figure 4B as well.

2) In Discussions, presynaptic LTP is defined as preLTP in the 2nd paragraph. Therefore, in page 19 first line, "...a presynaptic form of preLTP...," should be changed to just preLTP. The same goes for line 3 on the same page, "...mGluRs for presynaptic preLTP..."

3) P15: “In the presence of 8-CPT, a decrease in the number of failures in transmission was observed at P13-21 (15 ± 5% baseline, 10 ± 3%, in the presence of 8-CPT, n = 6) and P22-30 (18 ± 4% baseline, 5 ± 3%, in the presence of 8-CPT, n = 6) and at P35-42 (20 ±4% baseline, 1 ± 1% in the presence of 8-CPT, n = 6), suggesting a presynaptic action of adenosine on A1Rs.”. The authors did not refer to figure in the manuscript. They should at least mention “data not shown”. Also, the statistics should be mentioned here, at least the p value </> 0.0X, as displayed in Fig. 1F legend.

4) P19, line 2: “hippocampal CA3-CA1 synapses, a presynaptic form of preLTP that is independent of NMDA” a “R” is missing in “NMDA”.

5) P19, line 9: the reference “Meredith et al., 2003” appears in the text instead of a superscript numbering.

6) P25, line 22: a bracket “)” is missing after “learning and memory87” and also line 23: after “fear memory formation88”.

7) P25, line 24: “pre t-LTP” could be “presynaptic t-LTP” for more clarity.

8) For all the figures: the labelling referring to the representative trace samples (1; 1’; 1’’; 2; 2’; 2’’) is hard to read and make the graphs busy. For clarity purposes, I would suggest a color code such as black/gray for before/after plasticity induction, and the age/condition labelled on the bottom of the trace.

9) For all the figures: the legends of the time course graphs should have a standard location. Also the (n) on the histograms should be properly and standardly aligned, vertically and horizontally.

10) Abstract, last line: "have revealED a new form of presynaptic LTP"

11) There are generally too many unnecessary parentheses in the text, which break up the flow and make the text hard to read, e.g. p3 "activity (sensory)-dependent plastic changes", p4 "we found that a protocol (a post-before-pre protocol)", p4 "fluctuation (number of failures)", p4 "NMDARs (pre- or postsynaptic) ", p4 "but also glutamate (presumably to activate mGluRs)",

12) Page 3, "Thus, timing-dependent LTP (t-LTP) occurs when a presynaptic spike is followed by a postsynaptic spike, whereas timing-dependent LTD (tLTD) is induced when this order is inverted." --> "Typically, timing-dependent LTP (t-LTP) occurs when a presynaptic spike is followed by a postsynaptic spike, whereas timing-dependent LTD (tLTD) is induced when this order is inverted, although exceptions exist (refs)." where refs should be replaced by suitable citations.

13) p4, unclear what "must" means here, "astrocytes must not only release adenosine", please clarify.

14) p5, "male mice aged postnatal day (P) 13-42" -- why were only male mice used? Please justify. How was sex determined? Please clarify.

15) Comma missing in several places, e.g. p7 "Where appropriate, “Glutamate Puffs” were applied"; p 11 "by measuring changes in the PPR, we found that"; p23 "that when there is a high glutamate release probability, synapses are likely"

16) Page 7, these currents cannot really be correct, can they? It says "5 ms, 80-120 pA" but I think it should say "5 ms, 800-1200 pA" or alternatively "50 ms, 80-120 pA", or else the input resistance of these cells must be around 2 MOhm rather than around 200 MOhm.

17) Page 7, unclear "Glutamate was included in the external solution", you mean you puffed external solution with Glutamate, but it sounds like you perfused Glutamate.

18) Page 9, "We observed failures in synaptic transmission in several experiments", how many experiments out of how many? n = 8? This is not clear.

19) Page 18, I would recommend tying in the present findings with the hippocampus STDP debate, see papers by Jack Mellor (e.g. doi: 10.3389/fnsyn.2010.00011) and Sam Wang (Wittenberg Wang JNeurosci 26:6610). These findings can really explain a lot of the arguments in the literature.

20) Page 21 and elsewhere, as the authors themselves point out: "We previously showed that enhancing inhibition (mediated by adenosine and not by GABAergic receptors)", it is a bit confusing to speak of 'inhibition' in this context, since that typically refers to GABAergic transmission. Perhaps 'suppression' works better?

21) P21, "Our data clearly indicate " should be "Our data suggest "

22) P22 and elsewhere in the Discussion, there is a lot of talk about t-LTD considering that this is a paper that is about t-LTP. The discourse seems unbalanced.

23) The Discussion seems a bit verbose and repetitive, e.g. p26, these results have been stated several times before. Discussion should be shortened, overall.

**Author rebuttal, first version**

*Reviewer #1 (Remarks to the Author):

The authors show that a post-pre pairing protocol that induces LTD at P13-18, induces LTP at P35-42, and make the case that this is due to reducing of release probability over this period, due to tonic glial adenosine release, coupled with phasic glial glutamate release to assist in presynaptic potentiation.One major issue is the exclusive focus on presynaptic locus of t-LTP. They provide some evidence that there is an increase in presynaptic release probability after t-LTP (lowered PPR, lowered failure rate) but appear to perform no experiment whatsoever to address whether there is an post-synaptic component to t-LTP (is there an increase in Q? in mEPSC size? in unsilencing of synapses?). Thus the assertion that this form of LTP is exclusively presynaptically expressed is not justified.*

We thank the reviewer for rising this point. We would like to make clear that we did not make the assertion that “this form of LTP is exclusively presynaptic”. In fact, what we show is the opposite: this form of LTP really needs the “tripartite” synapse; i.e. this form of LTP needs the presynaptic neuron (to release glutamate), the postsynaptic one (we demonstrate that an increase in intracellular calcium levels into the postsynaptic neuron to release NO is necessary for this LTP to be induced). In addition, we demonstrate that this form of LTP needs astrocytes and astrocytic release of adenosine and glutamate.

What we claim that is presynaptic is the “expression” of this new form of LTP. We were based on paired-pulse and number of failures analysis that are indicators of presynaptic changes in the expression of the form of this plasticity (it is known that when these parameters change after the plasticity protocol the mechanism is most probably presynaptic but if they don´t, the mechanism is most probably postsynaptic). As we did observe changes in those parameters, they were indicative of presynaptic changes accounting for the expression of the LTP. We concur with the reviewer in the fact that more analysis can be done focused exclusively in postsynaptic parameters to confirm this presynaptic expression and definitely exclude postsynaptic changes or not. To directly address this point, we have performed new experiments and analysis and now we include the analysis of the “Coefficient of Variation” (CV), that make analysis of presynaptic and postsynaptic parameters directly, and as suggested by the reviewer, we performed experiments recording miniature responses (mEPSP, in the presence of TTX) measuring frequency and amplitude. These two approaches indicated that the expression of this form of LTP is exclusively presynaptic as the experimental points follow approximately a diagonal line in the analysis of the CV and we observed changes in the frequency but not in the amplitude of miniature responses after LTP induction what are directly indicative of not changes in postsynaptic parameters (no changes in the parameter Q).

These results all indicate that while the induction of this new form of LTP needs the presynaptic and postsynaptic elements (neurons) as well as astrocytes (i.e. needs the tripartite synapse), it is expressed as an increase in glutamate release probability only (having only a presynaptic expression), with no postsynaptic components.

*The key claim in this study is that astrocytes cause a developmental switch in spike time dependent plasticity from LTD to LTP. However, none of the astrocytic interventions at P35-42 convert LTP back to LTD, LTP is simply inhibited. Thus, there must be other changes that are needed for this switch to occur not involving astrocytes.*

We thank the reviewer for rising this point. However, we do not see the direct correlation between the facts that astrocytes cause a developmental switch from t-LTD to t-LTP and that astrocytic interventions do not convert back t-LTP to t-LTD. The possibility that astrocytes are causing the developmental switch from t-LTD to t-LTP and that acting on astrocytes does not reverse the process really exists as the possibility that other changes involving astrocytes occur. Additionally this switch takes weeks to happen and the acute action on slices might be not enough to reverse the process (assuming this is possible in nature). Based on our previous and present data, we may explain why reversing the main astrocytic action does not necessarily converts t-LTP into t-LTD in a different way. We found that the most probably explanation is because at P13-21, apart of higher probability of glutamate release than that at P35-42 (this probability of glutamate release can be really increased by blocking adenosine release from astrocytes at P35-42 as shown in our experiments), t-LTD needs the presence and activation of presynaptic NMDARs. It may be well the case that NMDARs are not available or functional at this age (P35-42) as the number of these receptors is known to decrease with development as we have shown recently (Pérez-Rodríguez et al., 2019). Thus, t-LTD disappears most probably due to the decrease or completely disappearance of preNMDARs with maturation that occurs in parallel (or even may be due to the increase of adenosine and activation or presynaptic A_1_R with maturation). Thus, the increase of adenosine produces a decrease in probability of glutamate release that may have two important consequences: one, there is less glutamate to activate preNMDARs that are located extrasynaptically, and second, there are less NMDARs available. At P35-42 changing adenosine levels and increasing glutamate release again do not seem to reverse the loss of preNMDARs necessary for t-LTD, what would make sense for physiological functions. Interestingly, and importantly, this presynaptic change seems to occur because of the effect of increasing A_1_R activation by adenosine that is of astrocytic origin. Thus, the central role of astrocytes creating the conditions for t-LTD or t-LTP is still present, highlighting the importance of astrocytes and their central role controlling the sign of plasticity with development.

*In a similar vein, the authors do not check the effect of any of the astrocytic interventions on P13-21 LTD, so even a differential role of astrocytes on plasticity at the two developmental stages is not proven. Overall the study is probably best suited to a specialist neurophysiology journal.*

In this point, we disagree with the reviewer. It can be seen in the manuscript that for instance, Figure 4 (now Figure 5) clearly showed different interventions at different ages including experiments from dnSNARE mutant mice that do not have astrocytic vesicular release operative. We are not sure whether it is necessary to make a list indicating where in the previous manuscript these approaches were shown: Page 13, paragraph 3, lines 1-2 “Astrocytes are implicated in t-LTD at the synapse studied here (Andrade-Talavera et al., 2016), participating in the closing of the windows of plasticity with maturation (Pérez-Rodríguez et al., 2019). Page 14, last paragraph “As we recently demonstrated that the activation of presynaptic A_1_R is responsible for the loss of t-LTD at P22-30 (Pérez-Rodríguez et al., 2019). Page 15, lines 8-17 for 8CPT and dnSNARE experiments. Figure 4D, E shows 8CPT results at P13-21, P22-30 and P35-42 in WT and dnSNARE mice. GDPBS into astrocytes experiments have been performed at P13-21 and P35-42 as indicated in the manuscript. Furthermore, we indicate in the Discussion section that the results of other approaches performed at other developmental stages (including P13-21) have already been published recently by the authors (Andrade-Talavera et al., 2016, 2016, Pérez-Rodríguez et al., 2019, references 10 and 11 in the previous version of the manuscript, now 11 and 12), what made, we think, the repetition of some of the experiments unnecessary. We think the confusion may come from the fact that we were continuously based on our previous results as we have already made those experiments many times previously (shown in the two publications indicated) that, if we may, we recommend to the reviewer to read in great detail to see that at P13-21 and P22-30 practically all of the astrocytic approaches we show here have already been performed and shown at P13-21 and at P22-30 (fluoroacetate, aBAPTA, aGDPBS, and others). We may concur with the reviewer that one approach, the study of nitric oxide (NO) in astrocytes that is new in this manuscript, has only been performed at P35-42 and no other ages, but this is only one of the experimental approaches. We did this experiment at P13-21 and observed that in the presence of NO synthase inhibitor L-NAME, t-LTD is not affected (as only t-LTD is observed at this developmental stage). These is now included in the text and shown in Supplementary Figure 3. In supplementary figures, we show most of the data obtained from experiments performed at P13-21.

References:

Andrade-Talavera, Y., Duque-Feria, P., Paulsen, O., Rodríguez-Moreno, A. Presynaptic spike timing-dependent long-term depression in the mouse hippocampus. *Cereb. Cortex* **26**, 3637-3654 (2016).

Pérez-Rodríguez, M., Arroyo-Garcia, L. E., Prius-Mengual, J., Andrade-Talavera, Y., Armengol, J. A, Pérez-Villegas, E. M., Duque-Feria, P., Flores, G., Rodríguez-Moreno, A. Adenosine Receptor-Mediated Developmental Loss of Spike Timing-Dependent Depression in the Hippocampus. *Cereb. Cortex* **29**, 3266-3281 (2019).

*Reviewer #2 (Remarks to the Author):*

*Prius-Mengual et al. find a switch in CA3-CA1 synaptic plasticity rules associated with anticausal spike-timing, which early in development elicits presynaptic LTD, but later on, owing to astrocyte-mediated adenosine signalling, elicits a novel form of presynaptic LTP that likely depends on calcium influx from postsynaptic L-VGCCs and internal stores, NO signalling, and activation of presynaptic mGluR5 by astrocytic glutamate release. Overall, the paper is well controlled, and has the potential to be of interest, drawing upon themes of plasticity, development, critical periods, and astrocytes. I do have some concerns, which I believe if addressed, could greatly enhance the impact of the work.*

We thank the Reviewer for the thorough revision and the positive comments on our work.

*Major Concerns:
1) Concern: The study is somewhat limited in scope. On one hand, it is amongst the few to extend our understanding of plasticity to beyond the developmental time period commonly investigated by saying that the plasticity rules do change in interesting ways with age, which certainly will be important for understanding network-level computations in the adult. However, on the other hand, the scope of this developmental change is not fully explored. The authors only look at a single spike timing interval, which gives an incomplete picture of how STDP rules change with development. I feel it would considerably increase the impact of the paper if the authors explored in greater detail the timing dependency of plasticity (ie construct a full STDP curve), so as to better understand synaptic changes across a range of spike timings. Without such information it is impossible to say whether t-LTD is truly abolished in the more mature rodent, or whether the STDP rules are just broadened or
shifted along the time axis, which again limits the scope of the authors’ findings. It is also difficult to say what exactly are the timing requirements for the t-LTP they describe, leaving the characterization of the novel plasticity they describe incomplete. Clarifying these points are important.*

We thank the reviewer for rising this interesting point and we appreciate the positive comment and suggestion. We agree that adding some more information regarding the timing-dependence of plasticity, to construct a full STDP and to determine the exact timing requirements of this new type of preLTP notably increases the scope and the impact of the paper. Accordingly, we have made the suggested experiments and completed the Spike Timing Dependent Windows (STDP). We have found that the switch from presynaptic t-LTD to presynaptic t-LTP occurs at intervals from at least -18 to -35 ms and thus, that the switch occurs at different timing intervals. These new results indicate that the switch from LTD to LTP is not related to a particular and unique timing and that is not the consequence of a shift in timing interval, and confirm that this switch occurs naturally with development. These results are now included in the text in the Results and Discussion Sections and shown in the new Figure 2.

*Interestingly, work from Huibert Mansvelder’s lab, and in particular the study Guilherme et al., 2010 (Frontiers in Neuroscience) shows that the STDP curve is indeed broadened at adult human hippocampal synapses (see their Figure 6) such that positive timing intervals yield LTP, short negative timing intervals (<80ms) also yield LTP, and only at long negative intervals (> 80ms) does LTD become apparent. This is of course very different from what was previously known about STDP in rodent hippocampus, which was assessed in developing animals. The general thinking, therefore, has been that human synapses are different from rodent ones. However, the findings of Guilherme et al., 2010 are not inconsistent with what the authors now show in their study, in which short negative timing intervals produce LTP in the more mature rodent. The question is what happens at longer negative timing intervals, would they also see LTD as in the human? If so, it would mean that synaptic learning rules
may be conserved across species. A more through exploration on the timing dependence of plasticity in the more mature rodent would therefore be very valuable to both the plasticity community, and to neuroscientists who are interested in the consequences of network-level learning rules in older animals, in which most in vivo work is conducted. Suggestion for address: The authors should broaden the scope of their study by assessing the impact of a range of spike timings on plasticity in the more mature rodent (P35-P42). I would follow the example of Guilherme et al., 2010 (Figure 6) in which intervals of -140 to +20 are sampled in one set of experiments, and compare findings with this study. Incidentally, to date I do not believe anyone has constructed an STDP curve of presynaptic LTP (which could be done using concurrently collected PPR data). It would be a very useful data set for those interested in presynaptic plasticity.*

We thank the reviewer for the thoughtful revision of these aspects and for the suggested experiments. As indicated above, we have done the experiments to construct a full STDP window for this novel form of preLTP. We found that at very long negative intervals (more negative than -75 ms) we do not observe t-LTD but we observe t-LTD until -50 ms (from -18 to -50 ms). Between -18 and -50 ms we studied 3 different timing intervals all showing a switch from t-LTD to t-LTP with maturation (-18, -25 and -35 ms, post-pre pairing protocol). We have constructed the STDP curve for this preLTP that we agree with the reviewer that it is the first STDP curve for a presynaptic form of LTP. Interestingly, we found that some of the discrepancies found in the literature when comparing data from humans and rodents may really come from the different ages of used animals. These new results, while not showing LTD in mice at very long negative intervals, show some similarities with what has been observed in human slices previously (for instance in Testa-Siva et al., 2010 as indicated by the reviewer), strongly suggesting that STDP curves are conserved across species in adults (at least at the many of the timing intervals studied). These results are included in the text, commented in the discussion section and shown in the Figure 2.

*2) Concern: Related to my second concern, the study is limited in its exploration of plasticity rules. Just because the authors’ particular STDP protocol shows an interesting developmental switch, does not imply that all STDP protocols will show similar effects. For example, given the reduction in release probability is important for promoting t-LTP over t-LTD, what would happen if short bursts of CA3 input (2 spikes at 100-200Hz; which are quite common in vivo) were anticausally paired with postsynaptic activity instead of a single presynaptic spike? Such presynaptic bursts would surely mobilize more glutamate release, which may, for example better recruit preNMDAR signalling to induce preLTD instead of preLTP. Rather than particular forms of plasticity appearing or disappearing then, developmental maturation, by altering for example by altering the strength of various molecular pathways, may lead to an alteration in the threshold signalling requirements for particular forms of
plasticity. When assessed by a single plasticity protocol this may give the illusion of the disappearance/appearance of plasticity forms.
Suggestion for address: It would greatly strengthen the paper’s conclusions to assess how physiological changes in glutamate release probability impact plasticity rules (ie STDP pairing of presynaptic bursts with postsynaptic spikes). If burst pairing abolishes or reduces preLTP this would provide very strong support the author’s model, in which preLTP may be driven by a decrease in transmitter release probability. This finding may also suggest that preLTD may not be fully abolished, but rather the threshold requirements for its activation has changed with development. The experiment would also greatly extend the authors findings into understanding how physiological changes in glutamate release impact plasticity rules, greatly enhancing the scope of the paper. I would therefore strongly recommend the authors do this experiment, but leave it only as a suggestion. At the very least, they should briefly discuss the limitation of their study, and in particular, how different
patterns of pre/post activity may have differential developmental-dependent outcomes. preLTD may therefore not be abolished with development, but its rules for inductions may be changed.*

We agree with the reviewer that more data related to the plasticity rules would improve the manuscript. Accordingly, we have done more experiments regarding the plasticity rules and have performed the proposed experiment and an additional one that for the sake of symmetry we consider interesting to fully understand the plasticity rules. We have found that short bursts of CA3 input (2 spikes at 100-200 Hz anti-causally paired with a single postsynaptic spike instead of a single presynaptic spike) at P35-42 do not affect the switch from t-LTD to t-LTP that is still present. This may indicate that the amount of glutamate released by this protocol is still not enough to induce t-LTD or it may be that as LTD induction needs (apart of higher probability of glutamate release than at P35-42), the presence and activation of presynaptic NMDARs and it may be the case that NMDARs are not available or functional at this age as the number of these receptors is known to decrease with development (Pérez-Rodríguez et al., 2019). Thus, more than a change in the induction rules for t-LTD with maturation, we think that t-LTD disappears most probably due to the decrease or lack in preNMDARs with maturation that may well be mediated by the increase in adenosine release from astrocytes and pre A_1_R activation that occurs with maturation. We have additionally performed another experiment pairing presynaptic single stimulation with two postsynaptic spikes at 100 Hz and again the switch is present and no affected by this experimental condition. These results suggest that a stronger postsynaptic depolarization has no effect in the switch. Thus, we have found a switch from t-LTD to t-LTP using 3 different protocol of STDP, extending the knowledge of the plasticity rules involved and indicating that different STDP protocols results in the same switch in plasticity.

*3) Concern: The study is conducted at a non-physiological (room) temperature. This is a principle issue when studying presynaptic plasticity, as presynaptic function is particularly sensitive to non-physiological temperatures, as is both NMDAR and NO signalling. For example, activity-dependent, and NO-dependent enhancement of presynaptic has been shown to depend on axonal excitability, but only at sub-physiological temperatures (McNaughton et al, 1994, PNAS). Indeed, the impact of NO on facilitating presynaptic function is temperature dependent and in one report, seen at room temperature but not at physiological temperatures (Williams et al., 1993, Neuron). Moreover, given that temperature is known to alter the distribution of release probabilities within the tissue (e.g Hardingham & Larkman, 1998; Volgushev et al., 2004), and the authors effect seems to be dependent on adenosine-mediated changes in release probability, there is a concern that their findings are limited to
non-physiological functions. With the growing interest of exploring and understanding in vitro phenomenon in vivo, it is important to keep experimental conditions as physiological as possible to ensure lasting impact of the work. Suggestion for address: The authors should assess whether their preLTP persists at physiological/near physiological temperatures (>30C). In principle this could be addressed if they conduct experiments suggested in Concern #1 at physiological/near physiological temperatures (>30C).*

We agree with the reviewer that performing the experiments at a more physiological temperature may help to discard some possible alternative explanation to some of the experiments performed and data presented (related to nitric oxide and adenosine, etc.). This concern was also raised by Reviewer 3. Accordingly, we have now repeated the majority of the experiments (while not properly indicated, some of them were already performed at nearly physiological temperature) we presented in the previous manuscript and now present new results from experiments performed at 30-34ºC. From these new experiments the results obtained are basically the same and conclusions have no changed. The switch from t-LTD to t-LTP is present, the preLTP is equally observed at 30-34ºC, as the requirement of the postsynaptic neuron and astrocytes releasing adenosine and glutamate and NO requirement. The figures now contain all of these new data obtained at this more physiological temperature.

*Minor concerns/suggestions:

1) The authors do not show individual data points on their bar graphs, and so it is difficult to gauge the variance and therefore quality of the data. Transparency in data is very important, and individual data can be very useful to modellers. For PPR measures, seeing this variance is useful since basal PPR may very well influence the magnitude and direction of the plasticity. Ideally, to help the reader understand the data the authors should plot individual data points on top of their bar graphs; where paired (ie in Figure 1E) the points should be connected by a line. Alternatively, the authors should provide the value of the points in a supplementary table.*

We agree with the reviewer. We now show individual data points in the figure histograms and data from the PPR graph connected.

*2) Related to the above, given the authors suggest that changes in transmitter release efficacy may underlie the developmental switch of t-LTD to t-LTP have the authors done a correlation between initial PPR and synaptic efficacy following STDP? This would be nice to include.*

We thank the reviewer for the suggestion. Related to this we include now in the manuscript a comparison of PPR at P13-21 (a post-pre protocol induces t-LTD) and at P35-42 (a post-pre protocol inducing t-LTP) and we found statistically significant differences in PPR, with more PPF at P35-42 than at P13-21. This is now included in the text and in new Supplementary Figure 7.

*3) The authors only show PPR data in Figure 1E, but not throughout their manipulations; however, it would be important to see that changes in PPR are following expected changes in synaptic efficacy; this would help strengthen the manuscripts conclusions. Where the authors do have PPR (and even the failure analysis data) for their manipulations they should show it, or at the very least quote in text or provide a supplementary figure/table.*

We thank the reviewer for the suggestion. We did not include these data to not make the figures more complex. Now we show these data in a table (Table 1) as suggested.

*4) The authors do individual t-tests for their statistics. Whilst their can be some statistical justification for doing this (especially if declared a priori), there is still a risk of inflating the family-wise error rate. The authors should really consider using an ANOVA (in the case of normally distributed data, or an equivalent model to assess group statistics) and post hoc tests to assess significance. Looking at the author’s data I hardly think this will change the conclusions of the study, especially since all the data tell a parsimonious story.*

We thank the reviewer for rising this point that was not clear enough in the previous manuscript. We include now more information of test performed for normality and as suggested, we have redone all the statistical analysis using ANOVA with a post hoc test when multiple comparisons have been made. As main result, the statistical significance of the results has not changed after this re-analysis. The thresholds for significance (one, two or three stars) are now made clearer. This information is now included in the text in the Methods section and in the figure legends.

*5) In Figure 2E there looks to be run-up in the baseline, in which case the LTP looks like a simple continuation of this run up. Is this baseline run-up genuine or just unfortunate sampling? Could it be something in the vehicle? If it is the case, it would be important to repeat the experiment without pairing to see what the run-up is over time, and to redo stats against this. Also can the authors please make clear in the methods what vehicles were used and what solvent reagents were dissolved in (ie DMSO and respective concentration).*

We thank the reviewer for rising this point. This was an unfortunate sampling (at least during the first 5 minutes of baseline). We have a control pathway that was stable for the whole experiment. Now, as we repeated this experiment at a more physiological temperature (30-34ºC), we show new graphs with new data, that do not show any run-up. The results obtained are basically the same that showed in the previous version of the manuscript. These new results are included in the text and shown in new Figure 3. Additionally, we include now in the Material and Methods section the vehicles used.

*6) Do the authors have a positive control for the use of GDPBS (Figure 6E,F) and of THL (Fig S2). These drugs show no effect but how do they know its working? Can the authors cite a previous paper using these drugs successfully with the same parameters used in this study? The use of GDPBS is their only evidence that the mGluR5s are presynaptic so it would be good to have some indication that this manipulation is working.*

We thank the reviewer for rising this point. Yes, we performed control experiments for these drugs. We included this information in the previous manuscript in page 10 for GDPBS but not for THL. We now include this information in the text for GDPBs and THL and make it clearer. These compounds were working in the test experiments as they both block the induction of t-LTD at P13-21 in interleaved non-treated control slices as we showed recently (Andrade-Talavera et al., 2016).

*7) The authors show that nimodipine, thapsigargin, and L-NAME into the postsynaptic neuron blocks t-LTP, giving the impression that postsynaptic L-VGCCs, internal stores, and NO synthase are involved; but how can they be sure these drugs are only acting postsynatpically and not impacting closely situated astrocytic/presynaptic terminals via diffusion. These drugs show some lipid solubility. Do the authors have a control or citation showing that these drugs would only act in the postsynaptic compartment if intracellular dialyzed. If not it would be important for the authors to clarify in the text that these drugs may be acting on non-postsynaptic targets; as such the authors should moderate their conclusions that postsynaptic L-VGCC and internal stores are required.*

We thank the reviewer for rising this point. While it would be possible that these compound cross the cell membranes, to do it and to pass to other cells, this would, in principle, require a relatively long time to occur. Additionally, to have any effect the same concentration or a relatively high concentration of that we put into the pipette has to reach other cells. Furthermore, even if a compound is able to cross a cell membrane during the duration of an experiment, after this, it may be exposed to the extracellular fluid where will be diluted and most probably washed out. For all these reasons, we do not see this very plausible. As an example that this does not occur, astrocytic L-NAME (that do not prevent t-LTP) has no effect on the postsynaptic cell (in which when included prevents t.-LTP) as no effect preventing t-LTP is observed in this experimental condition.

*If postsynaptic internal stores are important, how are they being recruited if not by mGluRs. There is evidence that postsynaptic VGCCs can do this (e.g Dittmer et al., 2017 Cell Reports, Johenning et al., 2015 Plos Bio -Fig 6). The authors should cite and very briefly discuss.*

We thank the reviewer for rising this point and for the suggested references. We have performed an additional experiment to determine whether it could be possible that calcium-induced-calcium release is required for t-LTP. We performed this experiment in the presence of ryanodine and observed that t-LTP induction was indeed prevented; indicating that activation of ryanodine receptors mediating calcium release from intracellular stores is required for t-LTP and therefore, it seems that calcium passing through VGCC produces calcium-induced calcium release. Additionally, we have also cited the manuscripts suggested and briefly discussed them.

*8) It would be really nice if the authors could include a figure of their proposed mechanism. This is especially useful for readers not in the immediate speciality, and for those who ‘figure read’ manuscripts, which I am afraid is quite often the case these days.*

We thank the reviewer for the suggestion. We include now a figure showing the proposed mechanism (Figure 7).

*9) I would suggest avoiding the use ‘fluctuation analysis’ (an unnecessary jargon in general), which really can mean a number of forms of analyses and so it is not very informative and would be distracting to general reader. I would just refer to it as failure rate or response failures (ie in the abstract ‘t-LTP was expressed presynaptically as witness by a decrease in response failures and the paired pulse ratio’).*

As suggested by the reviewer we do not use this expression in the manuscript.

*10) In the methods the authors suggest that ‘during the experiments, changes in Vm (1-3 mV) were corrected by imposing continues current’; is this wise? How do you know the changes were not due to offset/electrical drift?*

After the whole-cell configuration of the patch-clamp technique in current-clamp mode is established, the voltage changes we observed are not due to offset as the electrode is in contact to the cell not in the bath. These small changes seem to be due to real oscillations in Vm of the cells. They could be due to changes in the quality of the patching, but this is not the case as its quality is continuously monitored by measuring series resistance and other parameters and they did not change during the experiments. As the parameters measuring the quality of the patch did not change during the experiments (and in current clamp to compensate for voltage drops across series resistance is relatively easy by using the bridge-balance circuit) the changes should be due to physiological changes of Vm (very small depolarizations, etc.). As changes in Vm during the experiment in current -clamp due to the cell physiology may affect EPSP slope and/or amplitude, not very much but for precision, we think is more correct to perform the experiment at a constant cell Vm.

*11) Could the authors include the distance from the soma they placed the stimulating electrodes in the Methods section; useful to know how far up the dendritic tree they are, since this impacts plasticity rules, backprop efficiency, the effect of dialysis, etc, and would be useful to know for interpreting the results.*

A suggested by the reviewer we include now this information in the Methods section. Distance was 200-300 µm.

*12) In the methods the authors write ‘Glutamate was included in the external solution,’ which makes it seem that you bath applied it. I think you mean to say ‘Glutamate was dissolved in external solution’*

We thank the reviewer for noticing this typo. We indicate now that “Glutamate was dissolved in external solution” what is exactly what we did.

*13) The authors refer to astrocytic adenosine-mediated activation of presynaptic A1 receptors as ‘inhibition’ (ie subtitle in Results section). The use of inhibition and a stand-alone word is often associated with GABAergic transmission, so, in my opnion, it would be better to use another word or to at least say ‘inhibition of presynaptic release’ to avoid confusion.*

We thank the reviewer for the suggestion. To make this clearer we indicate now in the text “inhibition of presynaptic release as suggested”.

*14) I do not think it’s necessary to cite Cajal’s Croonian Lecture in reference to experience-dependent plasticity.*

We agree with the reviewer that it is not strictly necessary, however, as recently a debate is been open related to the first one to historically named “plasticity” we would like to honour the researcher that did this for first time. We changed a bit the sentence to make it clearer and more correct, indicating that Cajal named it “plasticity”.

*15) The authors miscite ref 24 in the statement ‘….the release of Ca2+ from intracellular stores is also required to induce t-LTD[11] and LTP at hippocampal synapses[24]; ref 24 – Padamsey et al (elife, 2017) did not show this. Also the sentence is misleading because stores are not always required for all forms of plasticity (for example, see recent review from Emptage lab: Padamsey et al., 2018 – The Neuroscientist) the authors should clarify this in text. Similarly, the authors write ‘the requirement of mglurs for presynaptic preLTP has previously defined’, however again mGluRs are not always necessary for all forms of LTP (nor all forms of preLTP); the authors should clarify this in text.*

We thank the reviewer for indicating this mistake. We apologize for it. We have now corrected this in the text.

*16) The authors cite refs 30-35 in support of ‘there is evidence that calcium influx though L-type calcium channels could participate in NO synthesis and/or its release from postsynaptic neurons. Ref 31-33 are not strong support of this; 32-33 do not show this at all. Reference 30 also claims specific coupling of NO production to NMDAR, and also use non-physiological stimuli to make their point. Reference 34 uses highly non-physiological stimuli to drive NO synthesis via L-VGCC activation (high K+ plus the LVGCC channel activator Bay K 8644). The particularly relevant citations for this statement should be 35 – Pigott et al., who first show L-VGCC dependent potentiation is also NO sensitive at CA3-CA1 synapses, and 24- Padamsey et al. (elife) who first show that physiological activation of L-VGCCs is sufficient to drive NO release from CA1 dendrites, which in turn increases release probability.*

We thank the reviewer for indicating this. We have now corrected this in the text.

*17) The authors write in the discussion ‘There is almost no information regarding the possible behavioural influence of presynaptic LTP’. Choi et al 2018 (Science) very beautifully show presynaptic (and postsynaptic) changes within the synaptic engram associated with context-dependent fear conditioning. Whilst it is not causal, it would be important to mention that preLTP is associated with learning and memory in vivo.*

We thank the reviewer for informing about this work. As suggested, we have now cited the paper and included this information in the text in the discussion section.

*18) The authors write they found a similar switch in plasticity using a 15Hz 1s LTD protocol, which was antagonized by 8-CPT. The authors should show this data as supplemental information as it supports their study. Otherwise the author should remove this sentence; without data the reader cannot evaluate the statement. On this note, the authors should cite.*

We thank the reviewer for the suggestion. We have removed the sentence for consistency and to refer all the results to Spike timing-dependent plasticity.

*19) To support their model, have the authors seen if addition of adenosine agonists can prematurely trigger preLTP in younger animals? This would be good support for their model (but is by no means required).*

We thank the reviewer for rising this point. This was also raised by Reviewer 3. We performed the experiment in the presence of the A_1_R agonist CPA (50 nM) at P13-21 (when the protocol induce t-LTD) and observed that in the presence of CPA, t-LTD was converted into t-LTP confirming the adenosine dependence of the switch. We additionally performed the experiment at P22-30 (when no LTD is induced by the used protocol) and observed a clear t-LTP in the presence of CPA. This is now included in the text and shown in the new Supplementary Figure 6.

*20) The authors mention in the discussion ‘our findings expand the repertoire of presynaptic LTPs.’ It would be good to give readers references to these different forms and/or to mention them.*

We thank the reviewer for this suggestion. We now cite a recent review regarding this subject (Monday et al., 2018) and include two additional references (McGuinness et al., 2010; Padamsey et al, 2017).

References:

Andrade-Talavera, Y., Duque-Feria, P., Paulsen, O., Rodríguez-Moreno, A. Presynaptic spike timing-dependent long-term depression in the mouse hippocampus. *Cereb. Cortex* **26**, 3637-3654 (2016).

McGuinness, L., Taylor, C., Taylor, R. D., Yau, C., Langenhan, T., Hart, M., Christian, H., Tynan, P., Donnelly, P., Emptage, N. Presynaptic NMDARs in the hippocampus facilitate transmitter release at theta frequency. *Neuron* **68**, 1109-1127 (2010).

Monday, H. R., Younts, T. J., Castillo, P. E. Long-Term Plasticity of Neurotransmitter Release: Emerging Mechanisms and Contributions to Brain Function and Disease. *Annu. Rev. Neurosci.* **41**, 299-322 (2018).

Padamsey, Z., Tong, R., Emptage, N. Glutamate is required for depression but not potentiation of long-term presynaptic function. *eLife* **6**, e29688 (2017).

Pérez-Rodríguez, M., Arroyo-Garcia, L. E., Prius-Mengual, J., Andrade-Talavera, Y., Armengol, J. A, Pérez-Villegas, E. M., Duque-Feria, P., Flores, G., Rodríguez-Moreno, A. Adenosine Receptor-Mediated Developmental Loss of Spike Timing-Dependent Depression in the Hippocampus. *Cereb. Cortex* **29**, 3266-3281 (2019).

Testa Silva, G., Verhoog, M. B., Goriounova, N., Loebel, A., Hjorth, J. J. J., Baayen, J. C., de Kock, C. P. J., Mansvelder, H. D. Human synapses show a wide temporal window for spike timing-dependent plasticity. *Front. Syn. Neurosci****.* 2**, 12 (2010).

*Reviewer #3 (Remarks to the Author):*

*Prius-Mengual et al. describes a novel form of NMDAR-independent timing-dependent long-term potentiation (t-LTP) at CA3 to CA1 synapses. The group previously showed that, at CA3-CA1 synapses, tLTD disappears at later stages (Pérez-Rodríguez 2019), and this study follows up on those findings. Using the analysis of failure rates and PPR change, they first demonstrated that this new form of t-LTP is expressed presynaptically. They then showed that its induction requires the activation of presynaptic mGlu5Rs but not NMDARs like for tLTD at earlier stages, and that postsynaptic Ca2+ and retrograde NO signalling from the postsynaptic neuron are required to induce this presynaptic t-LTP. Finally, they demonstrate that this form of presynaptic t-LTP requires astroglial signaling, which developmentally dampen neuronal glutamate release via the release of ATP/adenosine enabling the t-LTD-to-t-LTP switch. In summary, this t-LTP thus depends on mGlu5R, L-type calcium channels, nitric oxide,
and involves adenosine and glutamate release from surrounding astrocytes.

This manuscript is clearly written (although the Discussion is a bit repetitive and too long), it has novel findings, the figures are clear, and the data largely support the findings that are drawn. The science is solid and entirely believable, but the statistics somewhat flawed. We are, however, not sure that this manuscript is of sufficient novelty for publication in Nature Communications. The manuscript would be strengthened considerably if the below points were addressed in full.*

We thank the reviewer for the thorough revision and the positive comments on our work.

*MAJOR POINTS:

1) Experiments were carried out at room temperature instead of physiological temperatures. To ensure biological relevance, authors should clarify whether the forms of plasticity induced in their experiments would also be induced if the experiments were carried out closer to physiological temperature.*

We agree with the reviewer that performing the experiments at a more physiological temperature may help to discard some possible alternative explanation in some of the experiments presented (related to nitric oxide and adenosine, etc.). This concern was also raised by Reviewer 2. Accordingly, we have now repeated the majority (while not properly indicated, some of them were already performed at nearly physiological temperature) of the experiments we presented in the previous manuscript and now present new results from experiments performed at 30-34ºC. From these new experiments the results obtained are basically the same and conclusions have no changed. The switch from t-LTD to t-LTP is present, the preLTP is equally observed at this more physiological temperature, as the requirement of the postsynaptic neuron and astrocytes releasing adenosine and glutamate and NO requirement. The figures now contain all of these new data obtained at this more physiological temperature.

*2) The authors should clarify which forms of plasticity is induced in P31 - P34 mice.*

We thank the reviewer for rising this point. We agree that this information is of interest and should be included in the manuscript. We did not include these results related to P31-34 for being a “transition period” from no plasticity to LTP in which we observed no plasticity or LTP in approximately 50% of the cases (48% no t-LTP, 52% t-LTP). As from P35 onwards, t-LTP was observed in, practically, 100% of the cases, we decided to study this form of t-LTP from P35. We now explain this in the text.

*3) Please clarify the mechanism of action for thapsigargin. It inhibits sarco-endoplasmic reticulum Ca2+-ATPase which actively sequesters Ca2+ in stores. Therefore, there is a possibility that intracellular levels of Ca2+ remains unchanged, or even increases, with the application of thapsigargin. If so, then the conclusion that it is Ca2+ release that is required for this form of t-LTP would be challenged.*

We thank the reviewer for rising this point. Thapsigargin is known to deplete calcium stores and once depleted, if any signal activates again these stores, they cannot release calcium, so there is no calcium available for this next function or action as it is also known that calcium once is released from these stores does not stay in the cytoplasm because is sequestered by other mechanisms. Therefore, calcium is not available in the cytoplasm nor in these stores when a stimulus arrives to release again from internal stores. For studies related to the mechanism of action of thapsigargin see for instance Sehgal et al., J Biol Chem 2017, 292, 19656-19673 and references therein.

*4) I believe the dnSNARE mouse line works with a tetOff system. If so, please clarify in Methods section whether and/or when the dnSNARE mice were supplied with doxycycline to turn off transgene expression. This is important because if gliotransmission is always blocked then these mice would not be a good comparison with the fluoroactate and a-BAPTA treatment as they might exhibit different forms of plasticity altogether, especially since this form of t-LTP is supposed to be brought about by astrocyte-released adenosine through maturation.*

We thank the reviewer for rising this point. In our experiments, all the pups and mice were not DOX fed, meaning that the transgenes are continuously being expressed and therefore the exocytosis should be blocked in astrocytes. As we see the same effect blocking exocytosis for a long term (dnSNARE) or in the presence of fluoracetate (reversible astrocyte metabolism blocker present for at least one hour) or even BAPTA (blocking astrocyte intracellular Ca^2+^ signaling; also for long-time), this means that the mechanism does not require acute drop of ATP release. We and others have shown in the past that in dnSNARE, the neuronal activity is slightly higher due to the lack of ATP/Ado tonic inhibition. And this effect if visible even after weeks/months of dox removal (Figure 2A, B of Pascual et al. Science 10, 113-116; and Fig B, C of the Sardinha et al. Glia 65, 1944-1969). For clarity, this is now indicated in the text.

*5) Novelty is potentially not high enough. Even though the study is well-designed and the paper well-written, this study probably does not provide enough conceptual advance to make it suitable for publication in Nature Communications. This is because many of the ideas suggested here have already been proposed:*

We understand the reviewer may have doubts regarding the nature of the novelty of the presented results as the work covers many different fields simultaneously drawing upon themes of plasticity, development, critical periods, probability of release, presynaptic receptors physiology and astrocytes. To put all of these aspects of the puzzle together is complex. Also we agree that some ideas and suspicions (but no real experimental data) are normally present in the different fields that may make things even more complex. However, we are completely convinced the results we obtained and the conclusions we reached are completely novel and, furthermore, we think are very relevant for these different mentioned fields. Modestly, we think our work is a good example of how many different pieces of some puzzles may converge in new developments in Neuroscience. Besides, this revised version of our manuscript includes some new and relevant experiments that in our opinion have greatly broadened the scope of the work.

These new experiments and results are:

1. Experiments were performed at nearly physiological temperature to discard alternative interpretation of the data obtained at room temperature and to confirm physiological relevance of the data.

2. Coefficient of Variation and mEPSPs analysis to confirm only presynaptic components of t-LTP expression. CV analysis is congruent with a presynaptic expression of t-LTP. Miniature responses experiments (before and after t-LTP) show no changes in mEPSP amplitude (no changes in postsynaptic parameter Q).

3. We produced for first time a STDP curve (window) for a presynaptic form of LTP. We found the switch we observe is present at negative pairing timing intervals (0, -50 ms) and is not the result of shifting along time axis.

4. We compared STDP window of a preLTP in mice slices with a STDP window obtained from humans slices. Interestingly, important similitudes were found when comparing curve from mice at P35-42 with STDP curve obtained from adult human slices.

5. We performed additional experiments to better understand the plasticity rules and checked the effect of other STDP protocols (a bust 2 stimuli of presynaptic activity at 100 Hz + single postsynaptic spike and single presynaptic stimulation + burst of 2 AP, anti-causally paired). The 3 different protocols used induced preLTP at P35-42.

6. We performed experiments to show the action of a NO donor of preLTP, confirming NO is required for LTP induction.

7. We performed experiments to determine whether the probability of release at CA3-CA1 synapses change with maturation using evoked activity (PPR of evoked responses). We observed a decrease in probability of release with maturation.

8. We performed different control experiments that were all confirmatory of the results, including protocols applied at P35-42 that we repeated at P13-21, interestingly that t-LTD at P13-21 can be converted in t-LTP by A_1_R agonist CPA.

These together with previous results make that our novel and relevant results are:

1. We discovered a novel form of presynaptic LTP.

2. We thoroughly characterized the cellular and molecular mechanism of induction of this new form of presynaptic LTP. Additionally, we demonstrated that the expression of this LTP is completely presynaptic.

3. Interestingly, this form of presynaptic LTP arises with age as a switch from preLTD. No such type of switch from STD preLTD to STD preLTP has been previously described to the best of our knowledge.

4. We advanced in the knowledge of critical windows of plasticity and, importantly, we characterized for the very first time a plasticity window for a presynaptic form of spike timing-dependent form of LTP.

5. We characterized the requirements of spike timing dependent plasticity for this new form of pre-LTP showing that with different protocols the switch is still present and thus is not due to a very particular STDP protocol.

6. We compared this curve/window to one obtained from human slices and found that differently to what was previously described, the window shows similarities between rodent and humans, suggesting some phenomenon as the switch described might be conserved across species. This may solve some of the controversies in this field showing the importance and impact in the direction of plasticity depending of the moment of postnatal development it is studied. Importantly, we suggest that this may be a general phenomenon that happens with different types of plasticity during development.

7. We determined that astrocytes play a central role in plasticity by controlling this switch of plasticity by directly controlling the presynaptic side of the synapse, i.e. controlling the probability of glutamate release of presynaptic neurons, what was done done mainly by controlling the levels of extracellular adenosine and the activation of pre A_1_R that dampens probability of neurotransmitter release.

8. We found that two form of LTP coexist during development (and may be both present in adults at CA3-CA1 synapses) but with different expression loci. The “classical” postsynaptically expressed t-LTP and the novel form we describe here that is expressed presynaptically. The simultaneous expression of two type of LTP may have important consequences for synaptic physiology.

9. We describe an almost unstudied role for NO affecting astrocyte physiology. Only few reports are available regarding NO, plasticity and astrocytes.

We respond to the concrete comments made by the reviewer related to this point:

*- developmental switch of the sign of plasticity (post vs presynaptic NMDAR for tLTD; Corlew 2007, Larsen Philpot NN 2014).*

In Corlew et al 2007, a switch from pre vs postsynaptic role of NMDAR in LTD is shown. This paper does not show a change in the sign of plasticity but in the location of NMDARs type involved in LTD (in this paper no LTP is shown). We think these results are not directly related to what we show in the present work as conceptually the sign of plasticity is not altered in this work. In Larsen et al., 2014, again a change in the sign of plasticity is not exactly shown, what the authors show is just that in some cases a form of t-LTD that is present in young animals disappears with development but they do not show a switch from LTD to LTP as we show here. As a result, to the best of our knowledge what we show here is the first case of a switch in plasticity with development from STD preLTD to STD preLTP.

*- involvement of mGluRs in presynaptic plasticity.*

While the reviewer did not refer to a particular reference here, we indicate in the text (for instance in the Discussion Section, that there exist very few forms of presynaptic forms of LTP that seems to involve presynaptic mGluR, however these forms of LTP were elicited by different protocols and not by Spike timing-dependent plasticity protocols. Thus, to the best of our knowledge, the presynaptic form of t-LTP we show here is the first form of preLTP induced by a spike timing-dependent plasticity protocol, what we consider an important advance in the field of spike timing-dependent plasticity. Additionally, this new form of spike timing-dependent preLTP is induced by post-pre protocols. Also interesting is to consider that this form of preLTP is the product of a switch from LTD happening during development.

*- astrocyte involvement in the control of synaptic plasticity. Panatier, 2006 showed that astrocytes contribute via D-serine to synaptic metaplasticity in the hippocampus. It was also shown that tLTD in neocortex (Min Nevian 2012) and t-LTP in hippocampus is controlled by astrocytic signalling.*

We agree with the reviewer that the forms of plasticity he/she indicates involve astrocytes, however, it is important to consider that Panatier et al 2006 show a role for astrocytes related to D-serine and NMDARs in hypothalamic nucleus and not in the hippocampus as we show here involving adenosine (no D-serine as they show) and mGluR (no NMDARs as they show). In the same way, Min and Nevian show an involvement of astrocytes in LTD in somatosensory cortex and not in the hippocampus and they do not show any role of adenosine as we show here from astrocytes. They do not study any form of LTP. Additionally, in both works they perform experiments at very young animals and are not related to preLTP. It is important to note that our study has been performed in the hippocampus and studying a relatively wide range of ages and studying LTP not LTD. Here we show a role of astrocytes that release adenosine in young adults to control plasticity and participating in the induction of a preLTP. To the best of our knowledge this role of astrocytes in a presynaptic form of preLTP that appears with maturation is new and we think involve an important advance in the plasticity and astrocyte fields. Finally, those works did not study a switch on plasticity and just at a particular developmental moment what is also different from our results. The present story is related to a switch from t-LTD to t-LTP in the hippocampus involving astrocytes releasing adenosine.

*- the developmental regulation of astrocytes: from Foncelle 2018. “In the developing hippocampus a presynaptic tLTD at CA3-CA1 synapses requires D-serine signaling possibly released from astrocytes (Andrade-Talavera et al., 2016). Interestingly, the same STDP pairing protocol induces tLTP at later developmental stages suggesting the possibility that astrocytic coverage of neurons and modulation of STDP by gliotransmission may be developmentally regulated."*

Foncelle 2018 is a review paper. In this review, based, at least in part, on our previous experimental data and ideas it is discussed that astrocytes may be involved in plasticity and have a role with development, what has really already been demonstrated even by our group in the hippocampus (Andrade-Talavera et al., 2016). However, we think, this not affect the whole story we present here as the form of plasticity is different. In that review, the authors cite our work to make the suggestion. The importance and the novelty here is that we demonstrate with experimental data it is true and characterize its mechanism of action.

*- astrocytes can decrease transmitter release by activating presynaptic A1 adenosine receptors (Pascual 2005; Serrano 2006; Todd 2010). Following their activation via mGluR5 during basal synaptic transmission, astrocytes can even increase the efficiency of transmission in CA1 pyramidal cells through activation of presynaptic adenosine A2A receptors (Panatier 2011).*

We thank the reviewer for rising this point. The effect of adenosine activating presynaptic A_1_R and decreasing neurotransmitter release is well known as we stated in the manuscript. We do not discuss that. The novelty of our work is: first, that this known mechanism to dampen glutamate release at presynaptic terminals change during development and is important for plasticity, and second, that it is used by the synapses and centrally by astrocytes, that control the release of adenosine, with maturation, to create the conditions for LTP to occurs what to our knowledge has not been demonstrated. For this, we consider the data we present are novel and important for the plasticity and synaptic transmission fields. This, in our opinion, may have very important consequences in the field as the mechanism may happens for many other forms of plasticity and be a general mechanism the brain uses to switch synaptic plasticity from LTD to LTP with maturation, what can explain for instance why it is so difficult to induce LTD in adults.

In conclusion, and for the many reasons indicated we consider our results are novel and relevant to the field. The idea of all of these components working together in fact make sense to some previous results that isolated were difficult to explain and understand, as we show different mechanisms at different levels converge to make a nice picture of how all of these mechanism are involved in a developmental switch from depression to potentiation, we think, as a good synthesis of many years of work.

*6) The mechanism is not clear. “t-LTP involves enhanced inhibition due to astrocytic adenosine-mediated activation of presynaptic adenosine A1Rs”:*

*- does 8-CPT have any effect in P13-21 and P22-30 mice?*

We thank the reviewer for rising this point and giving us the opportunity to make this clearer. We think the reviewer refers most probably to the effect of 8CPT on plasticity (as he/she may also refer to the effect of 8CPT on EPSP slope). Regarding the effect of 8-CPT on EPSP slope we show these results in Figure 5 in WT and dnSNARE mutant mice at different developmental stages, showing an increase in EPSP slope with development in WT mice in the presence of 8CPT that is not present in dnSNARE mutant mice. Regarding the effect of 8-CPT on plasticity at P13-21 and P22-30, we indicated this in the text citing our previous work (Pérez-Rodríguez et al., 2019). As this may not be completely clear, now we have repeated these experiments at physiological temperature. As reported previously, 8-CPT has no effect on t-LTD at P13-21 (Figure 6 Pérez-Rodríguez et al., 2019) and recovers t-LTD, lost at P22-30 (Fig. S4B, Pérez-Rodríguez et al., 2019). These data are included now in the text and shown in new Figure S5.

*- would activating A1R by bath applying the A1R agonist CPA to dampen glutamate release probability (which could be verified by measuring a mEPSP frequency decrease) turn tLTD into tLTP in P13-21 mice, and no tLTD to t-LTP in P22-30 mice? This might require the glutamate puffs (as in Figure 4G) too, if astrocytes are not capable yet to release glutamate at these stages.This experiment would confirm the switch being driven by the dampening of neuronal glutamate release by astrocytic ATP/Adenosine release.*

We thank the reviewer for rising this point. As indicated in the manuscript we have previously published that CPA on P13-21 converts t-LTD in No t-LTD (i.e. it prevents t-LTD induction) when 20 nM CPA was used (Pérez-Rodríguez et al., 2019). We have now repeated the experiment using higher CPA concentrations (as the experiments on EPSP slope suggest an increase in A_1_R inhibition with maturation) and observed that t-LTD is in fact converted in t-LTP when 50 nM CPA is used. We additionally confirmed that CPA produces a decrease in the frequency of mEPSP (without having any effect on mEPSP amplitude). These results confirm the dampening of neuronal glutamate release by astrocytes ATP/Ado. At P22-30, CPA also converts no plasticity in t-LTP. These data are in the text and shown in Supplementary Figure 6.

*7) The statistical analysis is flawed. Page 8, "A normality and equal variance test was performed", but it is not stated which precise tests were performed, e.g. the F test, a goodness of fit test, or something else. Also, there are multiple comparisons in every single figure (Figure 1-4, Figures S1-S4), yet no post-hoc correction for multiple comparisons is applied according to the Methods section. The appropriate procedure would be to test for homoscedastity, then apply the appropriate ANOVA (or nonparametric equivalent, if not normally distributed). If outcome suggests it at the 0.05 level, pairwise comparisons would be allowed, adjusted for unequal variance if need be, with outcomes corrected post-hoc for multiple comparisons, using e.g. Bonferroni Dunn. Instead, the authors go straight for the pairwise comparisons and never correct post hoc for multiple comparisons, which increases the risk for false positives considerably. Finally, the authors need to state the thresholds
for one, two, and three star significance levels in the Methods.*

We thank the reviewer for rising this point that was not clear enough in the previous manuscript. This has also been raised by Reviewer 3. We include now more information of test performed for normality and as suggested, we have also redone all the statistical analysis using ANOVA with a post hoc test when multiple comparisons have been made. As a main result, the statistical significance of the results has not changed after this reanalysis. The thresholds for significance (one, two or three stars) are now made clearer). This information is now included in the text in the Methods section and in the figure legends.

*8) The present study is missing a theoretical framework to tie in the findings with. The findings are presented as just-so stories, e.g. p18 "there is a maturation associated shift from the involvement of NMDARs to mGluRs in hippocampal plasticity". It is never explained why Mother Nature would want it to be this way, or how this benefits brain functioning. Furthermore, pre-post LTP and post-pre LTP are post and presynaptically expressed, but the computational implications of this is never discussed. For example, a change in short-term dynamics upon induction of long-term plasticity will affect temporal coherence, see e.g. Abbott et al Science 1997 275:220-224, or Markram Tsodyks Nature 1996, but the same is not true for insertion of AMPARs, which would reduce signal to noise ratio, see e.g. Otmakhov Malinow Neuron 1993. Basically, expressing LTP pre or postsynaptically is not identical in terms of outcome even if the gain is changed by the same percentage. This should be
discussed in detail in the Discussion. The theoretical framework should be provided to the readership. In this context, I would say that sentences such as "adding to the repertoire of functional modulation and probably facilitating better control of synaptic transmission" (p19) are vague and not helpful to a reader seeking to better understand the implications of the present findings, chiefly because that statement is not specific.*

We thank the reviewer for rising this point. Regarding the shift from NMDA to mGluR in plasticity after the switch, we have added a paragraph to the discussion section suggesting that NMDAR could be better coupled to intracellular pathways involved in depress glutamate release whereas mGluR might be better coupled to intracellular mechanisms involved in increases of glutamate release. Regarding the existence of pre and postsynaptic LTP coexisting at CA3-CA1 synapses and the physiological meaning of different expression sites; while we included some paragraphs discussing these aspects in the previous version of the manuscript (pages 19 and 25), as suggested by the reviewer we have added now some more points to the discussion. While we consider this as a very interesting point, a much more detailed and extended discussion or speculation about it might be out of the scope of the present manuscript and may unbalance the discussion of other mechanistic aspects. For the particular aspect indicated by the reviewer, there already exist excellent reviews (as Costa et al 2017, which is included in the references list) and we refer to it in the text.

*9) Nitric oxide signalling is blocked but never elicited, so there is an asymmetry that does not support the interpretation for both need and sufficiency for NO signalling in this form of plasticity. Experiments with a nitric oxide donor should thus be carried out to warrant the conclusions drawn.*

We agree with the reviewer. Accordingly, we have performed experiments using a NO donor. When NO signalling was blocked in the postsynaptic cells, t-LTP was prevented and adding NO donor on astrocytes recovered t-LTP. Confirming the involvement of NO in t-LTP induction and indicating his role acting on astrocytes. This information is now included in the text and shown in new Figure 6.

*10) Page 16, "This glutamate presumably activates presynaptic mGluRs" (also see p26), why does the Glu have to come from glia? Why can it not come from the synapse itself, where mGluRs act as autoreceptors? The authors have not excluded this quite likely possibility.*

We thank the reviewer for rising this point. We think that our experiment blocking calcium-dependent release from astrocytes with aBAPTA suggest this. When the presynaptic neuron releases glutamate, evoked by extracellular stimulation, this glutamate seems to be not enough to allow the induction of t-LTP when astrocyte release is blocked. The fact that adding CPA does not recover t-LTP (in conditions that presynaptic release of glutamate is present) strongly suggests something else apart of adenosine from astrocytes is needed for the preLTP induction. As adding glutamate (glutamate puffs) recovers t-LTP when aBAPTA and CPA are present (as it is glutamate release from presynaptic neuron), we understand this suggests an astrocytic origin of this glutamate. Nevertheless, as the reviewer point out, it is also possible that presynaptic glutamate may also be necessary (but not sufficient) and only when both, glutamate from presynaptic neuron and from astrocytes is present, preLTP is induced. We now include this possibility in the text.

*11) Page 16, "We tested whether the probability of glutamate release decreases at CA3-CA1 synapses with maturation by measuring miniature EPSP", spontaneous release frequency is a poor measure of evoked release probability, e.g. see papers by Ege Kavalali showing that these forms of release do not rely on the same mechanisms. This should at least be discussed, but ideally addressed experimentally.*

We agree with the reviewer regarding that evoked and spontaneous release may occur by different mechanisms. Accordingly, additionally to the miniature response analysis, we have performed additional experiments on Evoked PPR at young and P35-42, and found an increase in PPF with maturation, indicating a decrease in release probability. The results obtained studying spontaneous and evoked release show a decrease in glutamate probability release with maturation. These data are included in the text and shown in new Supplementary Figure 7.

*MINOR POINTS:

1) For consistency, THL data should be included in Figure S2A. Similarly, it would be clearer for readers if fluoroacetate and 8-CPT data were included in Figure 4B as well.*

We agree with the reviewer. We have now included these data in the graph.

*2) In Discussions, presynaptic LTP is defined as preLTP in the 2nd paragraph. Therefore, in page 19 first line, "...a presynaptic form of preLTP...," should be changed to just preLTP. The same goes for line 3 on the same page, "...mGluRs for presynaptic preLTP..."*

This has been changed as suggested by the reviewer.

*3) P15: “In the presence of 8-CPT, a decrease in the number of failures in transmission was observed at P13-21 (15 ± 5% baseline, 10 ± 3%, in the presence of 8-CPT, n = 6) and P22-30 (18 ± 4% baseline, 5 ± 3%, in the presence of 8-CPT, n = 6) and at P35-42 (20 ± 4% baseline, 1 ± 1% in the presence of 8-CPT, n = 6), suggesting a presynaptic action of adenosine on A1Rs.”. The authors did not refer to figure in the manuscript. They should at least mention “data not shown”. Also, the statistics should be mentioned here, at least the p value </> 0.0X, as displayed in Fig. 1F legend.*

As the reviewer suggest, we indicate now “data not shown” and show statistics.

*4) P19, line 2: “hippocampal CA3-CA1 synapses, a presynaptic form of preLTP that is independent of NMDA” a “R” is missing in “NMDA”.*

The typo has been corrected.

*5) P19, line 9: the reference “Meredith et al., 2003” appears in the text instead of a superscript numbering.*

The typo has been corrected.

*6) P25, line 22: a bracket “)” is missing after “learning and memory87” and also line 23: after “fear memory formation88”.*

This is now corrected.

*7) P25, line 24: “pre t-LTP” could be “presynaptic t-LTP” for more clarity.*

Changed as suggested.

*8) For all the figures: the labelling referring to the representative trace samples (1; 1’; 1’’; 2; 2’; 2’’) is hard to read and make the graphs busy. For clarity purposes, I would suggest a color code such as black/gray for before/after plasticity induction, and the age/condition labelled on the bottom of the trace.*

We use now colours to differentiate different experimental conditions, we think now the graphs and figures are clearer.

*9) For all the figures: the legends of the time course graphs should have a standard location. Also the (n) on the histograms should be properly and standardly aligned, vertically and horizontally*.

This has been revised as suggested and followed as much as possible.

*10) Abstract, last line: "have revealED a new form of presynaptic LTP"*

This typo is now corrected.

*11) There are generally too many unnecessary parentheses in the text, which break up the flow and make the text hard to read, e.g. p3 "activity (sensory)-dependent plastic changes", p4 "we found that a protocol (a post-before-pre protocol)", p4 "fluctuation (number of failures)", p4 "NMDARs (pre- or postsynaptic) ", p4 "but also glutamate (presumably to activate. imGluRs)"*

We have now eliminated those parentheses as suggested by the reviewer.

*12) Page 3, "Thus, timing-dependent LTP (t-LTP) occurs when a presynaptic spike is followed by a postsynaptic spike, whereas timing-dependent LTD (tLTD) is induced when this order is inverted." --> "Typically, timing-dependent LTP (t-LTP) occurs when a presynaptic spike is followed by a postsynaptic spike, whereas timing-dependent LTD (tLTD) is induced when this order is inverted, although exceptions exist (refs)." where refs should be replaced by suitable citations.*

We agree with the reviewer, this has been changed accordingly.

*13) p4, unclear what "must" means here, "astrocytes must not only release adenosine", please clarify*.

We changed the sentence to make it clearer.

*14) p5, "male mice aged postnatal day (P) 13-42" -- why were only male mice used? Please justify. How was sex determined? Please clarify.*

That was a mistake. We apologize for the typo. We used animals from both sexes. In the new experiments, we have used animals of either sex too. This is now correctly indicated in the text in the Methods Section.

*15) Comma missing in several places, e.g. p7 "Where appropriate, “Glutamate Puffs” were applied"; p 11 "by measuring changes in the PPR, we found that"; p23 "that when there is a high glutamate release probability, synapses are likely"*

This is now corrected.

*16) Page 7, these currents cannot really be correct, can they? It says "5 ms, 80-120 pA" but I think it should say "5 ms, 800-1200 pA" or alternatively "50 ms, 80-120 pA", or else the input resistance of these cells must be around 2 MOhm rather than around 200 MOhm.*

We meant to write 0.1-0.5 pA. This is now corrected in the text.

*17) Page 7, unclear "Glutamate was included in the external solution", you mean you puffed external solution with Glutamate, but it sounds like you perfused Glutamate.*

This was also raised by reviewer 2. This is now corrected in the text to indicate that it was dissolved in the extracellular solution.

*18) Page 9, "We observed failures in synaptic transmission in several experiments", how many experiments out of how many? n = 8? This is not clear.*

Number of experiments (n = 10) is now indicated in the text as suggested.

*19) Page 18, I would recommend tying in the present findings with the hippocampus STDP debate, see papers by Jack Mellor (e.g. doi: 10.3389/fnsyn.2010.00011) and Sam Wang (Wittenberg Wang JNeurosci 26:6610). These findings can really explain a lot of the arguments in the literature.*

As suggested we include now a brief reference to these aspects of STDP debate.

*20) Page 21 and elsewhere, as the authors themselves point out: "We previously showed that enhancing inhibition (mediated by adenosine and not by GABAergic receptors)", it is a bit confusing to speak of 'inhibition' in this context, since that typically refers to GABAergic transmission. Perhaps 'suppression' works better?*

Also raised by Reviewer 2. As suggested we changed it and now indicate “inhibition of presynaptic release” to make it clearer.

*21) P21, "Our data clearly indicate " should be "Our data suggest "*

Changed as suggested.

*22) P22 and elsewhere in the Discussion, there is a lot of talk about t-LTD considering that this is a paper that is about t-LTP. The discourse seems unbalanced.*

We agree with the reviewer that a considerable part of the manuscript is related to the new form of preLTP and for this we removed some aspects of the discussion related to t-LTD, but as this preLTP is the results of a switch from preLTD, we feel it is also necessary to state this in the manuscript when necessary to properly understand the approaches used and the results obtained.

*23) The Discussion seems a bit verbose and repetitive, e.g. p26, these results have been stated several times before. Discussion should be shortened, overall.*

We revised the discussion and shortened it considerably as suggested by the reviewer.

References:

Andrade-Talavera, Y., Duque-Feria, P., Paulsen, O., Rodríguez-Moreno, A. Presynaptic spike timing-dependent long-term depression in the mouse hippocampus. *Cereb. Cortex* **26**, 3637-3654 (2016).

Corlew, R., Brasier, D. J., Feldman, D. E., Philpot, B.D. Presynaptic NMDA Receptors: Newly Appreciated Roles in Cortical Synaptic Function and Plasticity. *Neuroscientist* **14**, 609-625 (2008).

Costa, R. P., Mizusaki, B. E., Sjöström, P. J., van Rossum, M. C. Functional consequences of pre- and postsynaptic expression of synaptic plasticity*. Philos. Trans. Royal Soc. B* **372**, 20160153 (2017).

Foncelle, A., Mendes, A., Jedrzejewska-Szmek, J., Valcheva, S., Berry, H., Blackwell K. T., Venance, L. Modulation of spike-timing dependent plasticity: towards the inclusion of a third factor in computational models. *Front. Comput. Neurosci.* **12**, 49 (2018).

Min, R., Nevian, T. Astrocyte signalling controls spike timing-dependent depression at neocortical synapses. *Nat. Neurosci.* **15**, 746-753.

Larsen, R. S., Smith, I. T., Miriyala, J., Han, J. E., Corlew, R. J., Smith, S. L., Philpot, B. D. Synapse-specific control of experience-dependent plasticity by presynaptic NMDA receptors. *Neuron* **83**, 879-893.

Panatier, A., Theodosis, D. T., Mothet, J. P., Touquet, B., Pollegioni, L., Poulain, D. A., Oliet, S. H. Glia-derived D-serine controls NMDA receptor activity and synaptic memory. *Cell* **125**, 775-784.

Panatier, A., Vallée, J., Haber, M., Murai, K. K., Lacaille, J. C., Robitaille, R. Astrocytes are endogenous regulators of basal transmission at central synapses. *Cell* **146**, 785-798 (2011).

Pascual, O., Casper, K. B., Kubera, C., Zhang, J., Revilla-Sánchez, R., Sul, J. Y., Takano, H., Moss, S. J., McCarthy, K., Haydon, P. G. Astrocytic purinergic signalling coordinates synaptic networks. *Science* **310**, 113-116 (2005).

Pérez-Rodríguez, M., Arroyo-Garcia, L. E., Prius-Mengual, J., Andrade-Talavera, Y., Armengol, J. A, Pérez-Villegas, E. M., Duque-Feria, P., Flores, G., Rodríguez-Moreno, A. Adenosine Receptor-Mediated Developmental Loss of Spike Timing-Dependent Depression in the Hippocampus. *Cereb. Cortex* **29**, 3266-3281 (2019).

Sardinha, V.M., Guerra-Gomes, S., Caetano, I., Tavares, G., Martins, M., Reis, J.S., Correia, J.S., Teixeira-Castro, A., Pinto, L., Sousa, N., Oliveira, J.F. (2017). Astrocytic signaling supports hippocampal-prefrontal theta synchronization and cognitive function. *Glia* **65**, 1944-1960 (2017).

Sehgal, P., Szalai, P., Olesen, C., Praetorius, H. A., Nissen, P., Christensen, S. B., Engedal, N., Møller, J. V. Inhibition of the sarco/endoplasmic reticulum (ER) Ca^2+^-ATPase by thapsigargin analogs induces cell death via ER Ca^2+^ depletion and the unfolded protein response. J *J. Biol. Chem.* **292**, 19656-19673 (2017).

**Reviewer comments, second version:**

Reviewer #1 (Remarks to the Author):

The authors first showed a role for increased astrocyte-derived adenosine in spike timing-dependent plasticity in the hippocampus when they demonstrated that it is responsible for the developmental loss of spike timing-dependent LTD by causing a reduction in release probability (Pérez-Rodríguez, Cerebral Cortex 2019). In this subsequent study the authors show that this high level of astrocyte-derived adenosine in later development is also important for spike timing-dependent LTP at a post-before-pre protocol that produces LTD at earlier developmental stages. This makes sense, as factors which reduce release probability can facilitate presynaptic potentiation, as well as suppress presynaptic depression. As the authors acknowledge, other factors are at work here with regard to the developmental change in spike timing-dependent plasticity, since preventing the effects of astrocyte-derived adenosine in later development does not switch LTP back to LTD, so the title may be
overstating things somewhat. Nevertheless, this is an interesting follow-up study, improved with revision, which adds to our knowledge of what changing astrocyte-derived adenosine during development does. I have not, however, changed my opinion that this does not represent a sufficient conceptual advance for this general science journal.


Reviewer #2 (Remarks to the Author):

To the authors;

I am generally satisfied with the author’s address; the manuscript is much improved. However, I have one remaining concern that should be addressed prior to publication. The authors now do explore a range of timings for P35-42 animals, but it is a pity that they do explore negative timing intervals beyond –50 ms (Figure 2). I do not feel this is an adequate range of negative timings to definitely rule out that tLTD is truly abolished in P35-42 animals since it remains a distinct possibility that tLTD occurs at more negative intervals beyond -50ms (ie the timing rules of tLTD have merely shifted/broadened with development). Indeed, in humans (Testa-Silva et al., 2010) there is an absence of tLTD at pairing intervals smaller than -80ms but tLTD emerges at pairing intervals >-80ms (ie -80 to -140 ms). Had the authors explored timing intervals in this range (-80 to -140ms) in adult animals (P35-P42) they may have seen the same thing. Currently, the authors only show that tLTD is
absent at -50ms, but this by no means rules out that tLTD is not present at more negative intervals. The transition between LTP and LTD in an STDP curve will always have an intermediate region where no net plasticity is observed. I would strongly recommend that the authors explore tLTD at longer intervals beyond -50 ms (ie up to -140 ms) in P35-42 animals to give a complete STDP curve. If not, the authors cannot conclude that ‘this switch happens with maturation and is not due to a timing interval broadening or shifting along the time axis.’ They should mediate this conclusion, making it explicitly clear in the manuscript that that they cannot rule out that tLTD may be present at longer negative timing intervals (>-80ms), as is the case in humans, and therefore that it is in fact possible that there may be broadening or shift along the STDP time axis with development.

As a minor comment, on p11 the autors subtitle ‘The switch from t-LTD to t-LTP occurs across a wide range of spike timings. STDP curve.’ has an error.

Also, the authors write in the discussion ‘presynaptic t-LTP may contribute to circuit computation by changing short-term dynamics and it may shift synapses between low-pass and high-pass filtering modes, thereby changing the computational properties of the synapse.’ The authors may be interested in: ‘A two-compartment model of synaptic computation and plasticity' <https://doi.org/10.1186/s13041-020-00617-1.>

The manuscript as a whole elucidates a novel form of plasticity outside of the developmental age commonly explored; it will be of interest to general neuroscience community.

Sincerely,

Zahid Padamsey


Reviewer #3 (Remarks to the Author):

The authors have made an admirable effort and have addressed all our points in full. This is a very rich and detailed study that is making a solid contribution to the field. This manuscript is close to ready for publication in Nature Communications. We have a few minor additional suggestions:

1. Fig 1h, inset black trace seems blurry compared to red trace.

2. Figs 1e and f, the inset traces above the bars do not align well with the bars.

3. Fig 1g, The CV graph is not clear regarding the locus of expression of LTP. Showing the mean of all data points overlaid on individual data might help to clarify that the average behaviour is indeed on the diagonal, as claimed in the main text.

4. Related to point 3 above: Page 10, "A plot of CV-2 versus the change in the mean evoked EPSP slope (M) before and after t-LTP yielded points along the diagonal line indicating a presynaptic modification of release parameters17", this statement is strictly speaking not true. For presynaptic expression, the average must be above the diagonal, not on it. The average ending up on the diagonal suggests an on-average combined pre and postsynaptic expression, or that the binomial distribution does not describe release statistics at this synapse type (Poisson release stats would put it right on the diagonal, though), or that there are experimental measurement problems, or all of the above at once (Brock et al, FiSN 2020 12:11 DOI: 10.3389/fnsyn.2020.00011; Faber & Korn, Biophysical journal 1991 60:1288-1294). I think this caveat should be clarified to the readers — there is consistency between the CV and the PPR analyses, but also a some disagreement in that the CV analysis hints at
an additional postsynaptic contribution, which may be real or a flaw due to the CV analysis method itself.

5. Page 11, at top: "The switch from t-LTD to t-LTP occurs across a wide range of spike timings. STDP curve." The "STDP curve" bit seems out of place and should either be deleted or included in the rest of the section title.

As per the instructions for reviewers, I hereby opt to formally reveal my identity. Signed: Jesper Sjöström

**Author rebuttal, second version**

*Reviewer #1 (Remarks to the Author):

The authors first showed a role for increased astrocyte-derived adenosine in spike timing-dependent plasticity in the hippocampus when they demonstrated that it is responsible for the developmental loss of spike timing-dependent LTD by causing a reduction in release probability (Pérez-Rodríguez, Cerebral Cortex 2019). In this subsequent study the authors show that this high level of astrocyte-derived adenosine in later development is also important for spike timing-dependent LTP at a post-before-pre protocol that produces LTD at earlier developmental stages. This makes sense, as factors which reduce release probability can facilitate presynaptic potentiation, as well as suppress presynaptic depression. As the authors acknowledge, other factors are at work here with regard to the developmental change in spike timing-dependent plasticity, since preventing the effects of astrocyte-derived adenosine in later development does not switch LTP back to LTD, so the title may be
overstating things somewhat. Nevertheless, this is an interesting follow-up study, improved with revision, which adds to our knowledge of what changing astrocyte-derived adenosine during development does. I have not, however, changed my opinion that this does not represent a sufficient conceptual advance for this general science journal.*

We thank the Reviewer for the positive comments on our work and to consider it is improved with revision and it is an interesting study, which adds to our knowledge of what changing astrocyte-derived adenosine during development does. Regarding the reviewer indication that other factors may exist involved in the observed switch from t-LTD to t-LTP with development, we already agreed with the reviewer that this is a possibility but, as we indicated in the previous revision of the manuscript, these changes may all be due or caused by astrocytic functions during development. As we indicated and based on our previous and present data, reversing the main astrocytic action does not necessarily converts t-LTP into t-LTD most probably because at P13-21, apart of higher probability of glutamate release than that at P35-42 (this probability of glutamate release can be really increased by blocking adenosine release from astrocytes at P35-42 as shown in our experiments), t-LTD needs the presence and activation of presynaptic NMDARs. It seems probable that NMDARs are not available or functional at this age (P35-42) as the number of these receptors is known to decrease with development as we have shown recently (Pérez-Rodríguez et al., 2019). Thus, t-LTD disappears most probably due to the decrease or completely disappearance of preNMDARs with maturation that occurs in parallel (or most probably due to) the increase of adenosine and activation or presynaptic A_1_R with maturation. Thus, the increase of adenosine produces a decrease in probability of glutamate release that may have two important consequences: one, there is less glutamate to activate preNMDARs that are located extrasynaptically, and second, there are less NMDARs available. At P35-42 changing adenosine levels and increasing glutamate release again do not seem to reverse the loss of preNMDARs necessary for t-LTD, what would make sense for physiological functions. Interestingly, and importantly, this presynaptic change seems to occur because of the effect of increasing A_1_R activation by adenosine that is of astrocytic origin. For this reason, as astrocytes seem to be central to all of these functions, we do not see the title is overstating things. Thus, the central role of astrocytes creating the conditions for t-LTD or t-LTP is still present, highlighting the importance of astrocytes and their central role controlling the sign of plasticity with development.

*Reviewer #2 (Remarks to the Author):

To the authors;
I am generally satisfied with the author’s address; the manuscript is much improved. However, I have one remaining concern that should be addressed prior to publication. The authors now do explore a range of timings for P35-42 animals, but it is a pity that they do explore negative timing intervals beyond –50 ms (Figure 2). I do not feel this is an adequate range of negative timings to definitely rule out that tLTD is truly abolished in P35-42 animals since it remains a distinct possibility that tLTD occurs at more negative intervals beyond -50ms (ie the timing rules of tLTD have merely shifted/broadened with development). Indeed, in humans (Testa-Silva et al., 2010) there is an absence of tLTD at pairing intervals smaller than -80ms but tLTD emerges at pairing intervals >-80ms (ie -80 to -140 ms). Had the authors explored timing intervals in this range (-80 to -140ms) in adult animals (P35-P42) they may have seen the same thing. Currently, the authors only show that tLTD is
absent at -50ms, but this by no means rules out that tLTD is not present at more negative intervals. The transition between LTP and LTD in an STDP curve will always have an intermediate region where no net plasticity is observed. I would strongly recommend that the authors explore tLTD at longer intervals beyond -50 ms (ie up to -140 ms) in P35-42 animals to give a complete STDP curve. If not, the authors cannot conclude that ‘this switch happens with maturation and is not due to a timing interval broadening or shifting along the time axis.’ They should mediate this conclusion, making it explicitly clear in the manuscript that that they cannot rule out that tLTD may be present at longer negative timing intervals (>-80ms), as is the case in humans, and therefore that it is in fact possible that there may be broadening or shift along the STDP time axis with development.*

We thank the reviewer for the positive comments and the fact he is generally satisfied with the revision and find the manuscript much improved.

We agree with the reviewer in that more negative intervals may be studied to now about the existence of t-LTD and the switch to t-LTP at these more negative intervals. Accordingly, we have performed experiments at P35-42 at -75 and -100 ms (where we did not previously performed the experiment because no t-LTD was observed at P13-21). Additionally, we have performed experiments at P13-21 and P35-42 at -125 and -150 ms interval to match the intervals used in humans in the indicated paper (Testa-Silva et al., 2010). We have found that at -75 and -100 ms (P35-42) nor t-LTD nor t-LTP was found (no plasticity at these negative intervals as found at P13-21). At -125 and -150 ms no plasticity was found at P13-21 or at P35-42. These results indicate that this form of t-LTD is not present at more negative intervals than -50 ms. These new data are now included in the text and in the Figure 2.

*As a minor comment, on p11 the autors subtitle ‘The switch from t-LTD to t-LTP occurs across a wide range of spike timings. STDP curve.’ has an error.*

We agree with the reviewer. We have removed “STDP curve” to make the phrase correct.

*Also, the authors write in the discussion ‘presynaptic t-LTP may contribute to circuit computation by changing short-term dynamics and it may shift synapses between low-pass and high-pass filtering modes, thereby changing the computational properties of the synapse.’ The authors may be interested in: ‘A two-compartment model of synaptic computation and plasticity'* [*https://doi.org/10.1186/s13041-020-00617-1.*](https://doi.org/10.1186/s13041-020-00617-1.)

We thank the reviewer for informing about this work. We have now included it in the text in the discussion section and in the references list.

*The manuscript as a whole elucidates a novel form of plasticity outside of the developmental age commonly explored; it will be of interest to general neuroscience community.*

We thank the Reviewer for the thorough revision and the positive comments on our work.

*Reviewer #3 (Remarks to the Author):

The authors have made an admirable effort and have addressed all our points in full. This is a very rich and detailed study that is making a solid contribution to the field. This manuscript is close to ready for publication in Nature Communications.*

We thank the reviewer for the positive comments on our work.

*We have a few minor additional suggestions:

1. Fig 1h, inset black trace seems blurry compared to red trace.*

We thank the reviewer for noticing this. We have used a new sample of the same trace in Figure 1.

*2. Figs 1e and f, the inset traces above the bars do not align well with the bars.*

We thank the reviewer for noticing this. We have now aligned the traces with the bars. *3. Fig 1g, The CV graph is not clear regarding the locus of expression of LTP. Showing the mean of all data points overlaid on individual data might help to clarify that the average behaviour is indeed on the diagonal, as claimed in the main text.*

We thank the reviewer for the suggestion. We observed an error in the previous CV plot (one column data was not plotted correctly, we apologize for this) that we have corrected now. As suggested by the reviewer, we now include the mean of all data. As it can be seen, the average is above the diagonal. We have changed this in the text accordingly.

*4. Related to point 3 above: Page 10, "A plot of CV-2 versus the change in the mean evoked EPSP slope (M) before and after t-LTP yielded points along the diagonal line indicating a presynaptic modification of release parameters17", this statement is strictly speaking not true. For presynaptic expression, the average must be above the diagonal, not on it. The average ending up on the diagonal suggests an on-average combined pre and postsynaptic expression, or that the binomial distribution does not describe release statistics at this synapse type (Poisson release stats would put it right on the diagonal, though), or that there are experimental measurement problems, or all of the above at once (Brock et al, FiSN 2020 12:11 DOI: 10.3389/fnsyn.2020.00011; Faber & Korn, Biophysical journal 1991 60:1288-1294). I think this caveat should be clarified to the readers — there is consistency between the CV and the PPR analyses, but also a some disagreement in that the CV analysis hints at
an additional postsynaptic contribution, which may be real or a flaw due to the CV analysis method itself.*

We thank the reviewer for rising this point. As indicated above, we have now included the mean of all data. As it can be seen, the average is above the diagonal consistent, in principle, with a presynaptic locus of expression. As the reviewer indicates there are some uncertainties and limitations related to this type of analysis very well explained and indicated in Brock et al., 2020 that may, in some cases, make interpretation difficult (whether a binomial distributions of a Poisson is followed by data, the limited number of data in experiments, etc.). As in our data, while some variability exist, the average is above the diagonal, and fluctuations were evident in our recordings, we think the results are consistent with a presynaptic locus for t-LTP (as indicated by the reviewer) as this presynaptic locus was also suggested/confirmed by the other analysis used (failures, PPFR, minis). We have included two additional references related to this type of analysis (Korn and Faber, 1991; Brock et al., 2020) and a line in the discussion section related to this.

*5. Page 11, at top: "The switch from t-LTD to t-LTP occurs across a wide range of spike timings. STDP curve." The "STDP curve" bit seems out of place and should either be deleted or included in the rest of the section title.*

We agree with the reviewer. We have now removed “STDP curve” to make the sentence correct.

**Reviewer comments, third version:**

Reviewer #2 (Remarks to the Author):

I am satisfied with the address. The extended STDP curve very nicely supports the authors' findings that t-LTD is converted to t-LTP with development. This study provides an excellent fundamental and mechanistic basis on which to explore plasticity rules throughout development.

Sincerely,

Zahid Padamsey

We thank the reviewer for the positive comments

**Author rebuttal, third version:**

Reviewer #2 (Remarks to the Author):

I am satisfied with the address. The extended STDP curve very nicely supports the authors' findings that t-LTD is converted to t-LTP with development. This study provides an excellent fundamental and mechanistic basis on which to explore plasticity rules throughout development.

Sincerely,

Zahid Padamsey

We thank the reviewer for the positive comments
